# Supplementary material for: Whole genome sequencing identifies bacterial factors affecting transmission of multidrug-resistant tuberculosis in a high-prevalence setting
Source: Sci Rep. 2019 Apr 3;9:5602. doi: 10.1038/s41598-019-41967-8 (PMC6447560; doi:10.1038/s41598-019-41967-8)
Supplement: Supplementary file 1 — Supplementary Materials [file 41598_2019_41967_MOESM1_ESM.docx]

**Whole genome sequencing identifies bacterial factors affecting transmission of multidrug-resistant tuberculosis in a high-prevalence setting**

Avika Dixit^1,2^, Luca Freschi^2^, Roger Vargas^2^, Roger Calderon^3^, James Sacchettini^4^, Francis Drobniewski^5^, Jerome T. Galea^6^, Carmen Contreras^3^, Rosa Yataco^3^, Zibiao Zhang^2,7^, Leonid Lecca^2,3^, Sergios-Orestis Kolokotronis^8^, Barun Mathema^9^, Maha R. Farhat^2,10^

^1^Boston Children’s Hospital, Boston MA, ^2^Harvard Medical School, Boston MA, ^3^Socios En Salud, Lima, Peru, ^4^Texas A&M University, College Station, TX, ^5^Imperial College, London, UK, ^6^University of South Florida, Tampa FL, ^7^Brigham and Women’s Hospital, Boston MA, ^8^SUNY Downstate Medical Center, Brooklyn NY, ^9^Mailman School of Public Health, Columbia University, New York, NY, ^10^Massachussetts General Hospital, Boston, MA

**Corresponding Author:**

Avika Dixit

300 Longwood Ave, Mailstop 3103

Boston MA 02115, USA

Email: [avika.dixit@childrens.harvard.edu](mailto:avika.dixit@childrens.harvard.edu)

Telephone: 617-919-2900

**Supplementary Materials**

**Supplementary Methods**

Whole genome sequencing and variant calling:

DNA extraction was performed as follows from LJ culture: a big loop of colonies was lysed with lysozyme and proteinase K to obtain DNA using CTAB/chloroform extraction and ethanol precipitation. DNA was sheared into ~250bp fragments using a Covaris E220 Focused-ultrasonicator (Covaris), prepared using the TruSeq Whole-Genome Sequencing DNA sample preparation kit (Illumina) and sequenced on a HiSeq platform (Illumina) generating 125bp paired-end reads. Reads were trimmed using PRINSEQ setting average phred score threshold to 20^1^. Raw read data was confirmed to belong to MTB complex using Kraken^2^. Isolates with <90% mapping were excluded. Reads were aligned to H37Rv (GenBank NC000962.3) reference genome using BWA-MEM^3^. Duplicate reads were removed using PICARD^4^. We excluded any isolates with coverage <95% of known drug resistance regions (*katG*, *inhA* and its promoter, *rpoB*, *embABC* and *embB* promoter, *ethA*, *gyrAB*, rrs, rpsL, *gid*, *pncA*, *rpsA*, *eis* promoter) at 10x or higher. Variants were called using Pilon that uses local assembly to increase indel (insertions and deletions) call accuracy^5,6^. If allele frequency was <75%, the reference allele is implied by Pilon. Low confidence coordinates were filtered from all strains if >95% of strains did not have coverage of at least 10x at that site. Isolate lineage was confirmed using high confidence variant calls as previously described^7^.

In order to validate our findings on the implication of genetic variation in these regions, we applied the same WGS variant calling pipeline described above to 375 isolates from a large TB outbreak in London^8^.

Phylogenetic analysis:

A rapid bootstrap analysis (n = 1000) with search for best scoring maximum likelihood (ML) tree was performed using the generalized time reversible model (GTR)^9^ with the Γ distribution used to model among-site rate heterogeneity^10^. The most distant strain (M44) was designated as an outgroup. The most distant strain was identified by first generating a neighbor-joining tree^11^ that included H37Rv strain as an outgroup. The H37Rv reference genome sequence was subsequently excluded from the ML phylogenetic analysis in RAxML. A 50% majority-rule consensus tree was generated using the consensus() function implemented in the R package ape^12^. Trees generated from the two alignments were compared using the R package treespace^13^. A strict clock assumption did not hold by the likelihood ratio test implemented in MEGA7^14^ and the temporal signal from sampling dates was tested using on root-to-tip regression as implemented in TempEST^15^ and was not found to be the case (R^2^ = 3.35 x10^-2^).

Phylogenetic trees were visualized with phenotypic attributes using FigTree 1.4.3^16^ and edited using Adobe Illustrator 2018. To determine association between phenotype and genotype using phylogeny, the R package treeWAS was used^17^. We reported any SNP found to be significant by one or more of the phylogenetic convergence scores.

Confirming PE/PPE and indel variants:

Variants in PE/PPE regions and indels between closely related strains i.e. having ≤5 SNPs were confirmed via visualization using Integrated Genome Viewer^18,19^. We loaded the bam file output of bwa as well as a wig changes output file from Pilon. We visually determined if the reads provide adequate support for a SNP in the PE/PPE region or indel by assessing the number and quality of the reads calling the variant at that region and the degree of surrounding variation. Notably our visual inspection confirmed the Pilon variant calls in 55% of sites. The excluded 45% were consistently related to differentially low coverage in either of the strains. In future studies, this can be minimized by either excluding regions with low coverage in even a single strain from the alignment, or planning for higher sequencing coverage. This is the case in closely related isolates, a common scenario in WGS/transmission studies, where small differences are amplified. We also noted that there is often a mix of variant and reference alleles at each site. We applied the Pilon recommended threshold of >75% to indicate a variant allele and <75% to indicate reference allele at each site, as also described above under ‘Whole genome sequencing and variant calling’, but future efforts can explore lowering this threshold and determining if this reduced the probably of differential calls between pairs of close isolates.

CNV analysis: To identify if any of the SNPs identified in PE/PPE regions could be due to copy number variants (CNVs) leading to false positive, we undertook additional analyses as follows: We generated CNVs for each of the 61 isolates included in the study using the R package CNOGpro 1.1^20^. We used the bootstrap method with a window length of 100 bp to determine the CNV of genes and intergenic regions, respectively, and we cross-checked the visually confirmed variants identified in PE/PPE regions with these CNVs for each isolate using a custom script in R^21^. None of the variants in PE/PPE regions were found to be occurring in regions with CNVs.

Other data analysis:

To determine if transmission of the cluster occurred in one particular geographic direction, pairwise genetic and geographic distances between sequenced isolates were compared using a Mantel test^22^.

Propensity-to-Propagate analysis:

To study host-related factors that may be associated with transmission, the ‘propensity to propagate’ (PTP) method was used as previously described^23^. For this analysis, we included data on patient’s age, gender, disease classification as pulmonary or extrapulmonary, sputum smear positivity and alcohol and drug use. For drug use, any patient that reported using either marijuana or cocaine more than five times in past five years was categorized as a user. Place of birth, travel and data on if the patient was a healthcare worker was not available and these variables were accordingly not used to calculate PTP.

SNP simulations

In order to assess the accuracy of our SNP calling methodology for the genes *Rv0095c*, *Rv3327* and *esxV*, we evaluated our SNP calling pipeline on a set of simulated Illumina data sets. We collected a set of 54 complete genomes from NCBI. We then mapped *Rv0095c*, *Rv3327* and *esxV* to the corresponding coding sequence (CDS) region of each complete genome by (1) creating a window of 5 gene lengths (bp) to the left and to the right of each gene. By sliding this window across the complete genome, we scored the number of CDS regions that matched (in length) within each window. (2) For the highest scoring windows across the genome, we pairwise aligned and scored each CDS region to the gene and retained the highest scoring CDS region. (3) Some CDS regions mapped poorly in step (2) or did not exactly match the gene length; these mappings were dropped from further analysis. *Rv0095c* mapped 28 times of 54 simulations, *Rv3327* mapped 18 times of 54 simulations and *esxV* was successfully mapped for all 54 complete genomes.

Next, we took each SNP found in these genes and introduced it into the corresponding genomic region (with respect to the gene start/end coordinates) for each successful mapping. This yielded two versions of the complete genome, one without the SNPs (unaltered) and one with (altered). We called ‘naturally’ occurring SNPs between the complete genome and H37Rv with nucmer^24^. We then simulated Illumina reads off of the altered complete genome at similar quality to the data used in our study using ART^25^. We mapped the simulated Illumina read data to H37Rv and called SNPs with our SNP calling pipeline (Megapipe)^6^. After normalizing this new SNP set (from simulated read data) with the other SNP set (from the unaltered complete genome) we were able to determine (a) whether we could correctly call the SNPs we introduced into the altered complete genome with our SNP calling pipeline and (b) whether we introduced any spurious SNPs into the gene by mapping short-read data to H37Rv.

Thus, for each of three genes, we define a true positive to be a SNP that we introduced into the complete genome and could call with simulated data and a false positive to be a SNP that appeared in a gene that we did not introduce and that was not part of the ‘natural’ variation between the complete genome and H37Rv. The number of simulations for each gene corresponds to the number of successful mappings for that gene. For *Rv0095c*, we found we could call the introduced SNP 26/28 times. For *Rv3327*, we called the SNP 14/18 times and for *esxV,* we called the SNP 3/54 times. We did not find any false positive SNP calls in any of these genes across any simulations. Thus, while we may be underestimating the extent of the true variation in these genes, it is unlikely that any of these SNP calls arise from spurious calls.

PacBio Assemblies

We aimed to determine whether the SNPs associated with repetitive regions (Rv0095c A85V, Rv3327 .54W and esxV L23S) we called in our pool of clinical isolates were naturally occurring, that is whether these SNPs have been identified in other clinical isolates. Unlike Illumina short-read sequencing data, PacBio reads are much longer and have randomly distributed error profiles. This makes PacBio reads ideal for constructing microbial genomes and identifying variants in repetitive regions.

PacBio sequencing was carried out on five isolates at > 200x coverage. Briefly, we used Canu^26^ to *de novo* assemble the raw PacBio subreads. Then we used Circlator^27^ to close the assembly. PacBio’s implementation of Minimap2^28^ was used to map the raw PacBio reads to the closed genome from Circlator. We used Samtools^28^ to index the assembly fasta files and polished the assembly three times with Quiver^29^. The polished genome was further polished with Illumina reads. We implemented our standard short read mapping pipeline (MegaPipe)^6^ using the polished genome in place of H37Rv. The last step of this pipeline uses Pilon^5^ to correct the polished genome from the sorted BAM file. Four of five of our samples assembled into a single contig, the fifth assembled into 24 contigs and was dropped from our analysis.

We ran nucmer^24^ between each of the polished assemblies using H37Rv as the reference sequence to call SNPs. We identified all three SNPs in at least 1 clinical isolate, with esxV L23S occurring in four isolates, demonstrating that these SNPs do in fact occur in Mtb clinical isolates and are unlikely to have resulted from false positive SNP calls in the isolate pool for our primary study.

**References (Supplementary Methods)**

1. Schmieder, R. & Edwards, R. Quality control and preprocessing of metagenomic datasets. *Bioinforma. Oxf. Engl.* **27**, 863–864 (2011).

2. Wood, D. E. & Salzberg, S. L. Kraken: ultrafast metagenomic sequence classification using exact alignments. *Genome Biol.* **15**, R46 (2014).

3. Li, H. & Durbin, R. Fast and accurate short read alignment with Burrows-Wheeler transform. *Bioinforma. Oxf. Engl.* **25**, 1754–1760 (2009).

4. Picard Tools - By Broad Institute. Available at: http://broadinstitute.github.io/picard/. (Accessed: 27th April 2018)

5. Walker, B. J. *et al.* Pilon: An Integrated Tool for Comprehensive Microbial Variant Detection and Genome Assembly Improvement. *PLoS ONE* **9**, (2014).

6. A wrapper pipe for variant calling and genome assembly for M.tuberculosis: github.com/farhat-lab/megapipe. (Farhat Laboratory, 2018).

7. Coll, F. *et al.* A robust SNP barcode for typing *Mycobacterium tuberculosis* complex strains. *Nat. Commun.* **5**, 4812 (2014).

8. Casali, N. *et al.* Whole Genome Sequence Analysis of a Large Isoniazid-Resistant Tuberculosis Outbreak in London: A Retrospective Observational Study. *PLOS Med.* **13**, e1002137 (2016).

9. Lanave, C., Preparata, G., Saccone, C. & Serio, G. A new method for calculating evolutionary substitution rates. *J. Mol. Evol.* **20**, 86–93 (1984).

10. Yang, Z. Maximum likelihood phylogenetic estimation from DNA sequences with variable rates over sites: Approximate methods. *J. Mol. Evol.* **39**, 306–314 (1994).

11. Saitou, N. & Nei, M. The neighbor-joining method: a new method for reconstructing phylogenetic trees. *Mol. Biol. Evol.* **4**, 406–425 (1987).

12. Paradis, E., Claude, J. & Strimmer, K. APE: analyses of phylogenetics and evolution in R language. *Bioinformatics* **20**, 289–290 (2004).

13. Jombart, T., Kendall, M., Almagro-Garcia, J. & Colijn, C. *treespace: Statistical Exploration of Landscapes of Phylogenetic Trees.* (2018).

14. Kumar, S., Stecher, G. & Tamura, K. MEGA7: Molecular Evolutionary Genetics Analysis Version 7.0 for Bigger Datasets. *Mol. Biol. Evol.* **33**, 1870–1874 (2016).

15. Rambaut, A., Lam, T. T., Max Carvalho, L. & Pybus, O. G. Exploring the temporal structure of heterochronous sequences using TempEst (formerly Path-O-Gen). *Virus Evol.* **2**, (2016).

16. Rambaut, A. *FigTree*. (2006).

17. Collins, C. & Didelot, X. A Phylogenetic Method To Perform Genome-Wide Association Studies In Microbes That Accounts For Population Structure And Recombination. *bioRxiv* 140798 (2017). doi:10.1101/140798

18. Robinson, J. T. *et al.* Integrative genomics viewer. *Nat. Biotechnol.* **29**, 24 (2011).

19. Thorvaldsdóttir, H., Robinson, J. T. & Mesirov, J. P. Integrative Genomics Viewer (IGV): high-performance genomics data visualization and exploration. *Brief. Bioinform.* **14**, 178–192 (2013).

20. Brynildsrud, O., Snipen, L.-G. & Bohlin, J. CNOGpro: detection and quantification of CNVs in prokaryotic whole-genome sequencing data. *Bioinforma. Oxf. Engl.* **31**, 1708–1715 (2015).

21. Code used for analysis of Peru cluster: github.com/farhat-lab/peru_cluster. (Farhat Laboratory, 2019).

22. Guillot, G. & Rousset, F. Dismantling the Mantel tests. *Methods Ecol. Evol.* **4**, 336–344 (2013).

23. Nebenzahl-Guimaraes, H., Borgdorff, M. W., Murray, M. B. & van Soolingen, D. A novel approach - the propensity to propagate (PTP) method for controlling for host factors in studying the transmission of Mycobacterium tuberculosis. *PloS One* **9**, e97816 (2014).

24. Kurtz, S. *et al.* Versatile and open software for comparing large genomes. *Genome Biol.* **5**, R12 (2004).

25. Huang, W., Li, L., Myers, J. R. & Marth, G. T. ART: a next-generation sequencing read simulator. *Bioinforma. Oxf. Engl.* **28**, 593–594 (2012).

26. Koren, S. *et al.* Canu: scalable and accurate long-read assembly via adaptive k-mer weighting and repeat separation. *Genome Res.* **27**, 722–736 (2017).

27. Hunt, M. *et al.* Circlator: automated circularization of genome assemblies using long sequencing reads. *Genome Biol.* **16**, 294 (2015).

28. Li, H. Minimap2: pairwise alignment for nucleotide sequences. *Bioinforma. Oxf. Engl.* **34**, 3094–3100 (2018).

29. Chin, C.-S. *et al.* Nonhybrid, finished microbial genome assemblies from long-read SMRT sequencing data. *Nat. Methods* **10**, 563–569 (2013).

**Supplementary Figures and Tables**


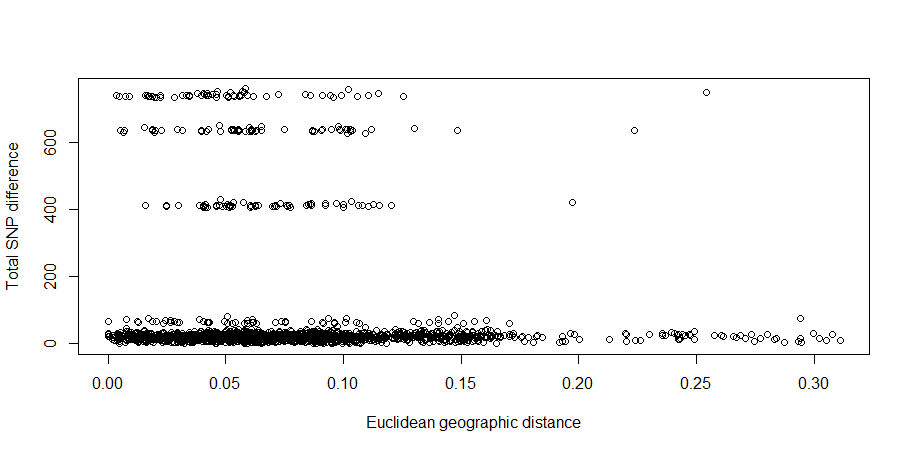

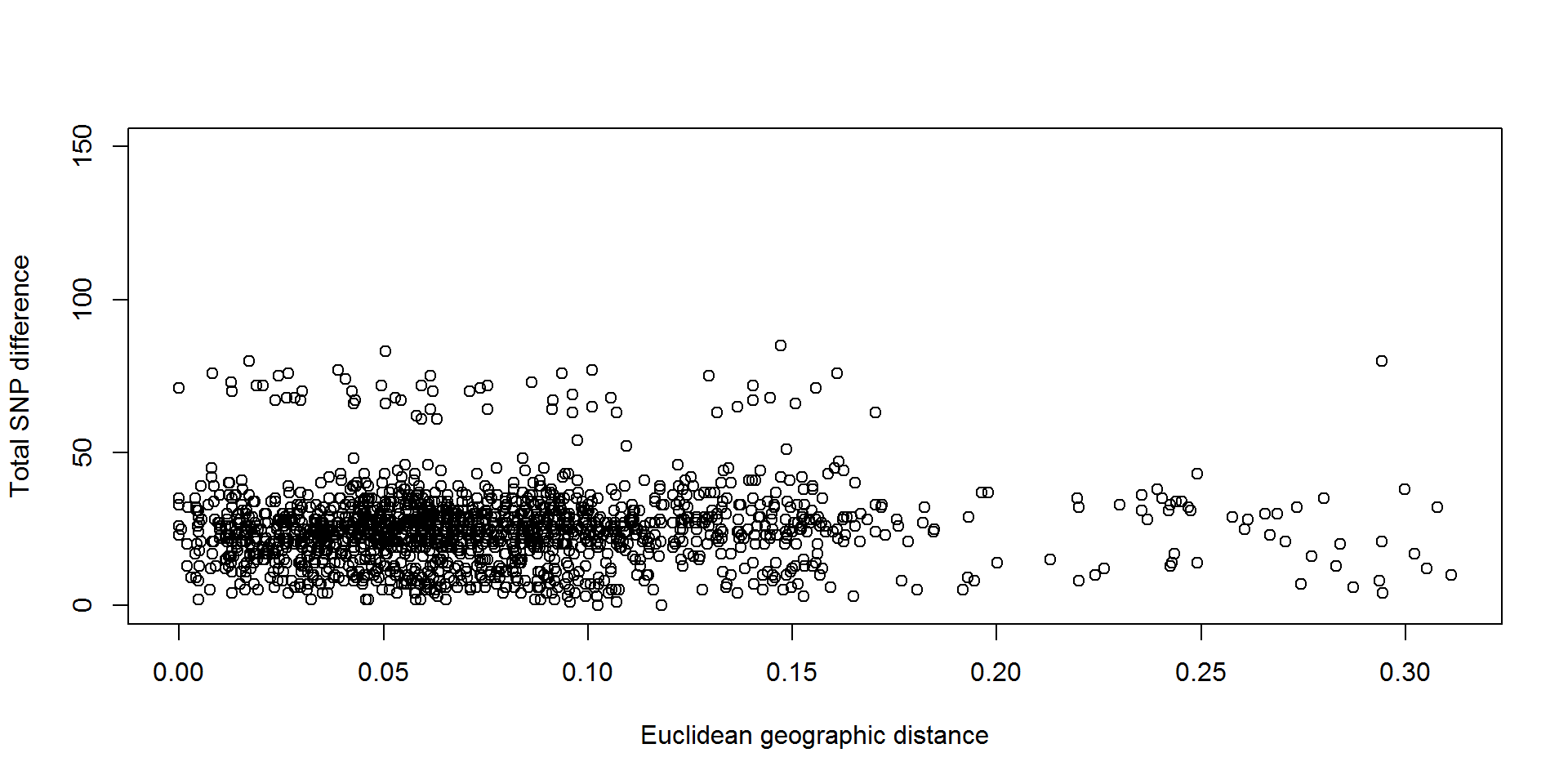


Supplementary Figure 1: Comparison of Single Nucleotide Polymorphism (SNP) distance and geographic distance. Each point represents distance between a pair of isolates. Three isolates with > 400 SNP distance from all other isolates are not shown in the bottom panel.


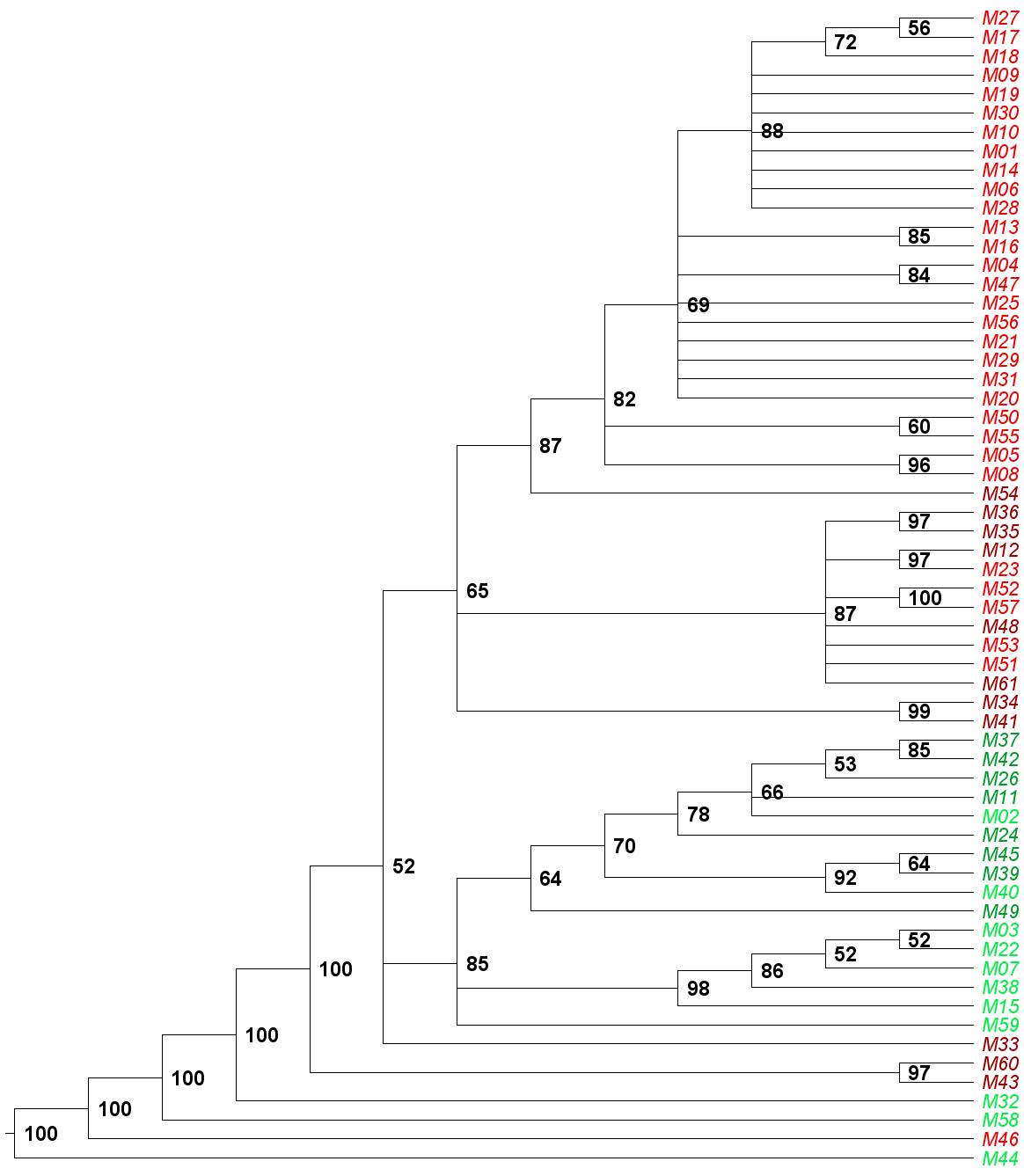


Supplementary Figure 2: Consensus phylogenetic tree. Numbers at nodes are bootstrap values. Nodes with bootstrap values < 50 have been collapsed. Color of tip represents drug susceptibility - Green: pan-susceptible, Dark Red: Resistant only to Isoniazid or Rifampicin, Dark Green: Resistant to a drug other than Isoniazid or Rifampicin, Red: multi-drug resistant.


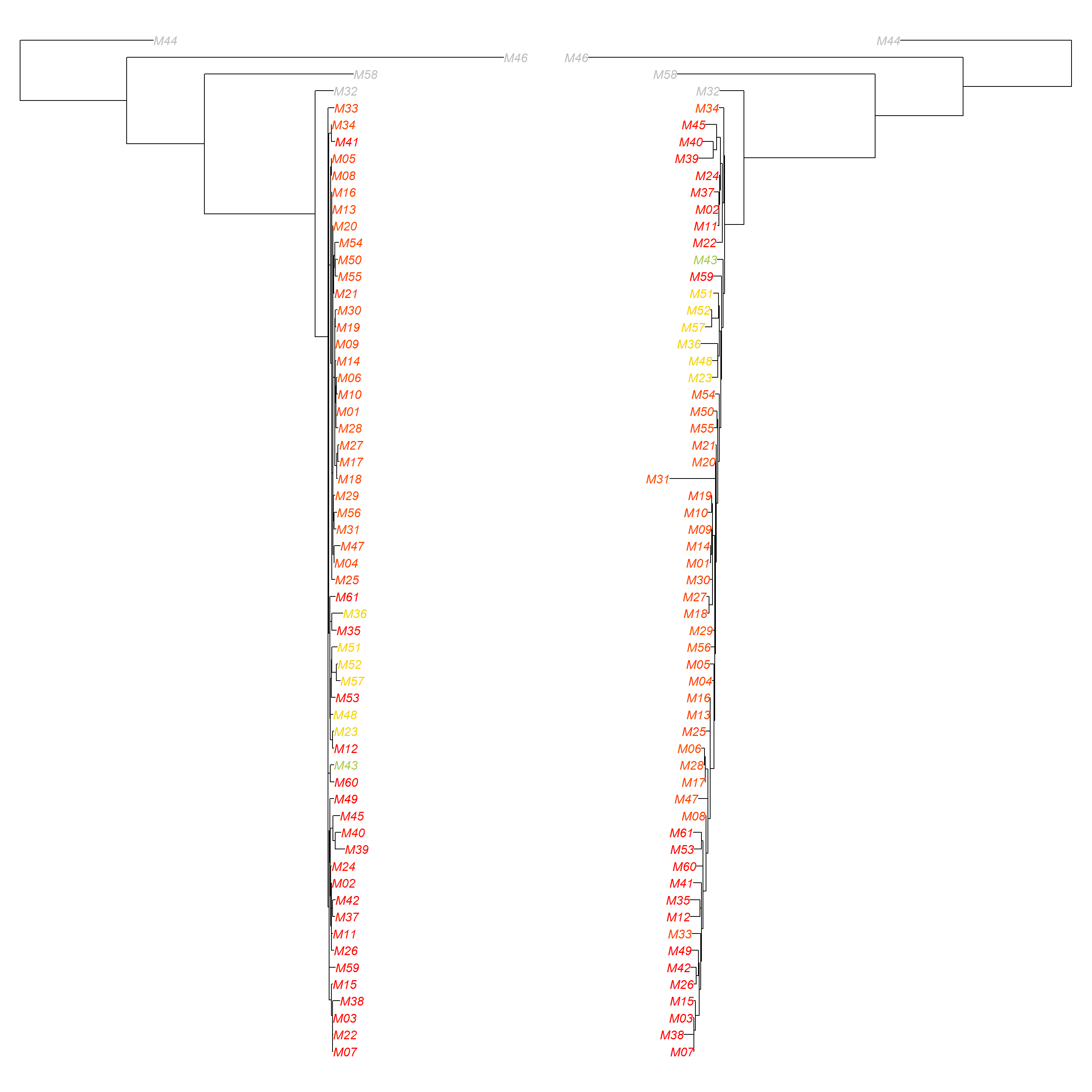


Supplementary Figure 3: Comparison of phylogenetic trees created with RaxML using SNPs only (left) and SNPs and indels (right). If a strain is colored the same in both trees, it indicates similar depth of ancestral branching.

**
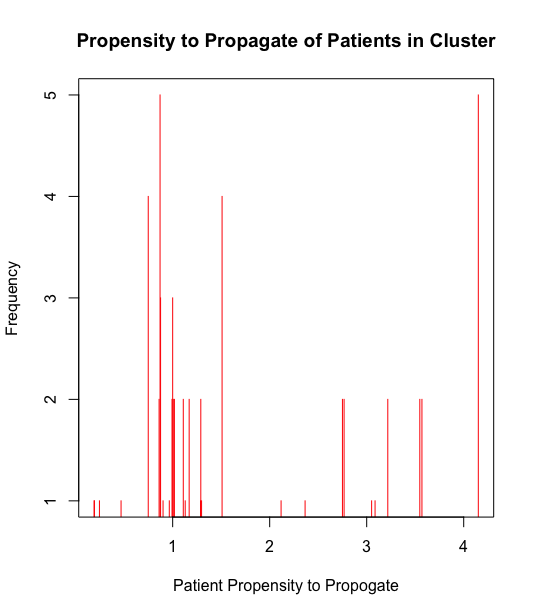
**

Supplementary Figure 4: Propensity to Propagate of 61 isolates based on patient demographic factors.

**
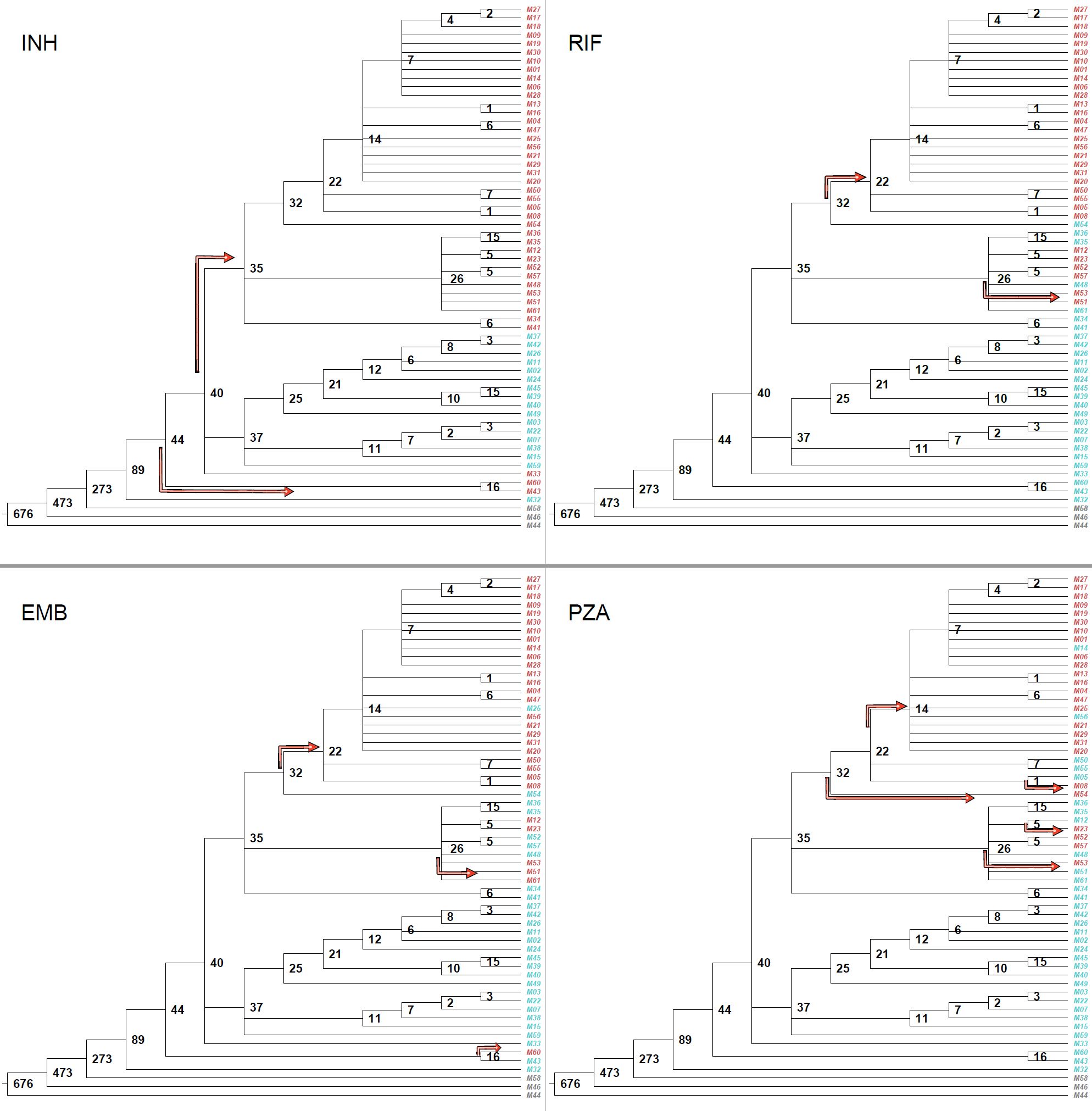
**

Supplementary Figure 5: Consensus phylogenetic trees with drug susceptibility overlay for A) Isoniazid (INH) B) Rifampicin (RIF) C) Pyrazinamide (PZA) D) Ethambutol (EMB). Red: Resistant, Blue: Susceptible, Grey: Strains not belonging to LAM-4.3.3. Lineage. Numbers at nodes are posterior means of node ages (years ago). Nodes with bootstrap values < 50 have been collapsed. Arrows indicate branches where drug resistance arose.

**Supplementary Table 1:** Frequency of observed polymorphisms in genes associated with drug resistance (silent mutations are not shown)**.**

| **SNP** | **Number with polymorphism (N = 61)** | **Estimated acquisition (years)*** | **Strains containing polymorphism**** |
| --- | --- | --- | --- |
| *INH* |  |  |  |
| SNP_CN_2155168_C944G_S315T_katG | 42 | 40, 37, 35 |  |
| SNP_P_1673432_T8C_promoter.fabG1.inhA | 2 | 20 | M05, M08 |
| SNP_CN_2518919_G805A_G269S_kasA | 58 | <273 |  |
| SNP_I_472705_T76C_inter.ndhA.Rv0393 | 59 | <473 |  |
| *RIF* |  |  |  |
| SNP_CN_761032_A1226G_Q409R_rpoB | 1 | 26 | M53 |
| SNP_CN_761093_G1287C_Q429H_rpoB | 2 | 26 | M12, M23 |
| SNP_CN_761109_G1303T_D435Y_rpoB | 2 | 26 | M12, M23 |
| SNP_CN_761110_A1304T_D435V_rpoB | 25 | 32 |  |
| SNP_CN_761139_C1333A_H445N_rpoB | 1 | 26 | M61 |
| SNP_CN_761155_C1349T_S450L_rpoB | 4 | 26 | M51, M52, M53, M57 |
| SNP_CN_761998_T2192C_L731P_rpoB | 1 | 26 | M51 |
| SNP_CN_762724_G2918A_G973D_rpoB | 1 | 26 | M53 |
| SNP_CN_764916_T1547C_L516P_rpoC | 2 | 26 | M52, M57 |
| *PZA* |  |  |  |
| SNP_CN_2289105_G137T_A46E_pncA | 1 | 26 | M53 |
| SNP_CN_2289213_T29C_Q10R_pncA | 24 | 32 |  |
| SNP_CN_2289231_A11G_L4S_pncA | 2 | 26 | M52, M57 |
| *EMB* |  |  |  |
| SNP_CN_4242182_G2320T_A774S_embC | 40 | 89, 44, 23, 14, 2.6, 4.8 |  |
| SNP_CN_4246893_A380G_D127G_embB | 1 | 89 | M32 |
| SNP_CN_4247431_G918A_M306I_embB | 2 | 26, 4 | M12, M61 |
| SNP_CN_4247469_A956C_Y319S_embB | 23 | 22, 14, 7 |  |
| SNP_CN_4247717_C1204G_L402V_embB | 1 | 7 | M50 |
| SNP_CN_4247730_G1217C_G406A_embB | 1 | 26 | M51 |
| SNP_CN_4248003_A1490G_Q497R_embB | 1 | 26 | M53 |
| DEL_P_4243205_d28T_promoter.embA.embB | 1 | 26 | M53 |
| *STR* |  |  |  |
| SNP_CN_781687_A128G_K43R_rpsL | 1 | 7 | M55 |
| SNP_P_781395_T165C_promoter.rpsL | 61 |  |  |
| SNP_I_1471659_C187T_inter.murA.rrs | 61 |  |  |
| SNP_I_1473637_A21G_inter.rrs.rrl | 1 | 26 | M51 |
| SNP_N_1472944_G1099A_rrs | 2 | 19.6 | M05, M08 |
| INS_CF_4407884_i319G_107G_gid | 5 | 24, 15, 6.5, 3 | M02, M11, M37, M45, M49 |
| SNP_CN_4407934_A269G_L90P_gid | 2 | 44 | M43, M60 |
| SNP_CN_4407952_G251A_P84L_gid | 20 | 15 |  |
| SNP_CN_4407973_A230C_V77G_gid | 9 | 32, 4.8 |  |
| SNP_CN_4408156_A47C_L16R_gid | 48 | 43, 40, 32, 24, 22.6, 15, 11, 8, 6, 4.8, 3, |  |
| *ETH* |  |  |  |
| INS_CF_4326802_i672G_225A_ethA | 1 | 1.8 | M27 |
| SNP_CN_4326248_A1226G_M409T_ethA | 1 | 26 | M53 |
| SNP_CN_4326842_T632C_Y211C_ethA | 2 | 19.6 | M05, M08 |
| *Fluoroquinolones* |  |  |  |
| SNP_CN_7362_G61C_E21Q_gyrA | 61 |  |  |
| SNP_CN_7585_G284C_S95T_gyrA | 60 | <473 |  |
| SNP_CN_8040_G739A_G247S_gyrA | 58 | <273 |  |
| SNP_CN_9304_G2003A_G668D_gyrA | 60 | <473 |  |
| *Capreomycin* |  |  |  |
| SNP_CN_1918634_G695A_G232D_tlyA | 7 | 6.85 |  |
| *Cycloserine* |  |  |  |
| SNP_CN_3336825_T1093C_T365A_ddl | 56 | 14, 8, 3, 0.87 |  |
| SNP_I_3336587_T209A_inter.Rv2980.ddl | 39 | 89, 35, 26, 25, 20, 16, 15, 8, 7, 6 |  |

*For estimation of acquisition times, only 57 internal strains were considered.

**Strain name is listed only if a polymorphism was seen in less than or equal to five strains.

**Supplementary Table 2:** TreeWAS results

| **Drug** | **SNP** | **Terminal score**  **(*P*-value)** | **Simultaneous score**  **(*P*-value)** | **Subsequent score**  **(*P*-value)** |
| --- | --- | --- | --- | --- |
| Isoniazid | SNP_CN_2155168_C944G_S315T_katG | 0.97 (<0.001) | 3 (<0.001) | 119 (<0.001) |
| Rifampicin | SNP_CN_761110_A1304T_D435V_rpoB | 0.84 (<0.001) | 3 (<0.001) | NS |
| Pyrazinamide | SNP_CN_104915_T301C_T101A_Rv0095c | NS | 2 (<0.001) | NS |
| Ethambutol | SNP_I_3778221_G347A_inter.spoU.PE-PGRS51 | 0.77 (<0.001) | NS | NS |

**Supplementary Table 3:** MIRU Pattern of 148 strains

| MIRU02 | Mtub04 | ETRC | ETRD | MIRU40 | MIRU10 | MIRU16 | Mtub21 | MIRU20 | Qub11b | ETRA | Mtub29 | Mtub30 | ETRB | MIRU23 | MIRU24 | MIRU26 | MIRU27 | Mtub34 | ETRE | Mtub39 | Qub26 | Qub4156 | MIRU39 |
| --- | --- | --- | --- | --- | --- | --- | --- | --- | --- | --- | --- | --- | --- | --- | --- | --- | --- | --- | --- | --- | --- | --- | --- |
| 1 | 3 | 4 | 2 | 2 | 4 | 3 | 4 | 2 | 2 | 2 | 4 | 1 | 2 | 6 | 1 | 5 | 3 | 3 | 2 | 2 | 5 | 2 | 2 |

**Supplementary Data**

**Supplementary Data 1:** Accession number of strains used for identification of fitness related SNPs

00R0025

00R0086

00R0178

00R0223

00R0308

00R0312

00R0435

00R0453

00R1156

00R1399

00R1547

00R1549

00R1566

01R0153

01R0166

01R0185

01R0238

01R0239

01R0244

01R0265

01R0272

01R0276

01R0290

01R0420

01R0451

01R0647

01R0685

01R0697

01R0737

01R0774

01R0878

01R0880

01R0897

01R0899

01R0902

01R0903

01R0904

01R0909

01R1018

01r1038

01R1241

01R1305

01R1321

01R1339

01R1341

01R1386

01R1387

01R1466

01R1468

01R1499

01R1505

01R1599

0209397

0209688

0209856

0209928

0210028

0210075

0210098

02R0016

02R0099

02R0119

02R0236

02R0237

02R0241

02R0272

02R0286

02R0325

02R0328

02R0360

02R0407

02R0417

02R0754

02R0759

02R0793

02R0812

02R0861

02R0890

02R0911

02R0948

02R0951

02R0971

02R0984

02R1076

02R1101

02R1106

02R1114

02R1137

02R1140

02R1142

02R1203

02R1210

02R1244

02R1262

02R1275

02R1288

02R1444

02R1457

02R1479

02R1485

02R1527

02R1543

02R1544

02R1589

02R1630

02R1641

02R1645

02R1681

02R1685

02R1687

02R1709

02R1723

02R1726

02R1728

02R1742

02R1753

02R1789

02R1793

02R1825

02R1854

02R1871

02R1915

02R1940

02R1941

02R1942

02R1945

02R1952

0310017

0310019

0310150

03R0058

03R0061

03R0068

03R0070

03R0110

03R0154

03R0177

03R0194

03R0221

03R0293

03R0319

03R0324

03R0327

03R0411

03R0419

03R0655

03R0736

03R0749

03R0768

03R0780

03R0783

03R0795

03R0797

03R0821

03R0871

03R0878

03R0908

03R0920

03R0929

03R0951

03R0979

03R0986

03R1082

03R1084

03R1176

03R1337

03R1338

03R1373

03R1404

03R1451

03R1465

03R1504

04R0266

04R0273

04R0292

04R0438

1478813216721T177115lib4769nextseqn0035151bp

1478813216721T177215lib4770nextseqn0035151bp

1478813216721T177315lib4771nextseqn0035151bp

1478813216721T177415lib4772nextseqn0035151bp

1478813216721T177515lib4773nextseqn0035151bp

1478813216721T177615lib4774nextseqn0035151bp

1478813216721T177715lib4775nextseqn0035151bp

1478813216721T177815lib4776nextseqn0035151bp

1478813216721T177915lib4984nextseqn0036151bp

1478813216721T178815lib4993nextseqn0036151bp

1478813216721T178915lib4994nextseqn0036151bp

1478813216721T179015lib4995nextseqn0035151bp

1478813216721T179115lib4996nextseqn0035151bp

1478813216721T179215lib4997nextseqn0035151bp

1478813216721T179315lib4998nextseqn0035151bp

1478813216721T179415lib4999nextseqn0036151bp

1478813216721T179515lib5000nextseqn0036151bp

1478813216721T179615lib5001nextseqn0036151bp

1478813216721T179715lib5002nextseqn0036151bp

1478813216721T179815lib5003nextseqn0036151bp

1478813216721T179915lib5004nextseqn0035151bp

1478821006466T180815lib5013nextseqn0036151bp

1478821006466T180915lib5014nextseqn0036151bp

1478821006466T181015lib5015nextseqn0036151bp

1478821006466T181115lib5016nextseqn0036151bp

1478821006466T181215lib5017nextseqn0036151bp

1478821006466T181315lib5018nextseqn0036151bp

1478821006466T181415lib5019nextseqn0036151bp

1479144119992T178115lib4986nextseqn0035151bp

1479144119992T178215lib4987nextseqn0035151bp

1479144119992T178315lib4988nextseqn0035151bp

1479144119992T178415lib4989nextseqn0036151bp

1479144119992T178515lib4990nextseqn0036151bp

1479144119992T178615lib4991nextseqn0036151bp

1479144813357T180115lib5006nextseqn0035151bp

1479144813357T180215lib5007nextseqn0035151bp

1479144813357T180315lib5008nextseqn0036151bp

1479144813357T180415lib5009nextseqn0035151bp

1479144813357T180515lib5010nextseqn0035151bp

1479144813357T180615lib5011nextseqn0035151bp

1479144813357T181615lib5021nextseqn0035151bp

1479144813357T181715lib5022nextseqn0035151bp

1479144813357T181815lib5023nextseqn0035151bp

1479144813357T181915lib5024nextseqn0036151bp

1479144813357T182015lib5025nextseqn0035151bp

1479146369267T182115lib5026nextseqn0035151bp

1479146369267T182215lib5027nextseqn0035151bp

1479146369267T182315lib5028nextseqn0035151bp

1479146369267T182415lib5029nextseqn0036151bp

1479146369267T182515lib5030nextseqn0036151bp

1479146369267T182615lib5031nextseqn0036151bp

1479146369267T182715lib5032nextseqn0036151bp

1479146369267T182815lib5033nextseqn0036151bp

1479146369267T182915lib5034nextseqn0036151bp

1479146369267T183015lib5035nextseqn0036151bp

1479146369267T183115lib5036nextseqn0036151bp

1479146369267T183215lib5037nextseqn0035151bp

1479146369267T183315lib5038nextseqn0035151bp

1479146369267T183415lib5039nextseqn0035151bp

1479146369267T183515lib5040nextseqn0036151bp

1479146369267T183615lib5041nextseqn0035151bp

1479146369267T183715lib5042nextseqn0035151bp

1479146369267T183815lib5043nextseqn0035151bp

1479146369267T183915lib5044nextseqn0036151bp

1479146369267T184015lib5045nextseqn0036151bp

1479146369267T184115lib5046nextseqn0035151bp

1479146369267T184215lib5047nextseqn0035151bp

1479146369267T184315lib5048nextseqn0036151bp

1479146369267T184415lib5049nextseqn0036151bp

1479146369267T184515lib5050nextseqn0036151bp

1479146369267T184615lib5051nextseqn0036151bp

1479146369267T184715lib5052nextseqn0035151bp

1479146369267T184815lib5053nextseqn0035151bp

1479146369267T184915lib5054nextseqn0035151bp

1479146369267T185015lib5055nextseqn0035151bp

1479146369267T185115lib5056nextseqn0035151bp

1479146369267T185215lib5057nextseqn0035151bp

1479146369267T185315lib5058nextseqn0035151bp

1479146369267T185415lib5059nextseqn0035151bp

1479146369267T185515lib5060nextseqn0035151bp

1479146369267T185615lib5061nextseqn0035151bp

1479146369267T185715lib5063nextseqn0036151bp

1479146369267T185815lib5062nextseqn0035151bp

1479146369267T888215lib5603nextseqn0048151bp

1479146369267T888315lib5604nextseqn0048151bp

1479146369267T888415lib5605nextseqn0048151bp

1479146369267T888515lib5606nextseqn0048151bp

1479146369267T888615lib5607nextseqn0048151bp

1479146369267T888715lib5608nextseqn0048151bp

1479146369267T888815lib5609nextseqn0048151bp

1479146369267T888915lib5610nextseqn0048151bp

1479146369267T889015lib5611nextseqn0048151bp

1479146369267T889115lib5612nextseqn0048151bp

1479146369267T889215lib5613nextseqn0048151bp

1479146369267T889315lib5614nextseqn0048151bp

1479149361993T889415lib5615nextseqn0048151bp

1479149361993T893015lib5699nextseqn0050151bp

1479149361993T893115lib5700nextseqn0050151bp

1479149361993T893215lib5701nextseqn0050151bp

1479149361993T893315lib5702nextseqn0050151bp

1479149361993T893415lib5703nextseqn0050151bp

1479149361993T893515lib5704nextseqn0050151bp

1479149361993T893615lib5719nextseqn0051151bp

1479149361993T893715lib5720nextseqn0051151bp

1479149361993T893815lib5721nextseqn0051151bp

1479149361993T893915lib5722nextseqn0051151bp

1479149361993T894015lib5723nextseqn0051151bp

1479149361993T894115lib5724nextseqn0051151bp

1479149361993T894215lib5725nextseqn0051151bp

1479149361993T894315lib5726nextseqn0051151bp

1479149361993T894415lib5727nextseqn0051151bp

1479149361993T894515lib5728nextseqn0051151bp

1479149361993T894615lib5729nextseqn0051151bp

1479149361993T894715lib5730nextseqn0051151bp

1479149361993T894815lib5731nextseqn0051151bp

1479149361993T895015lib5732nextseqn0051151bp

1479149361993T895115lib5733nextseqn0051151bp

1479149361993T895215lib5734nextseqn0051151bp

1479149361993T895315lib5735nextseqn0051151bp

1479149361993T895415lib5736nextseqn0051151bp

1479150601544T895515lib5737nextseqn0051151bp

1479150601544T895615lib5738nextseqn0051151bp

1479150601544T895715lib5739nextseqn0051151bp

1479150601544T895815lib5740nextseqn0051151bp

1479150601544T895915lib5741nextseqn0051151bp

1479150601544T896015lib5742nextseqn0051151bp

1479150601544T896215lib5744nextseqn0051151bp

1479150601544T896315lib5745nextseqn0051151bp

1479150601544T896415lib5746nextseqn0051151bp

1479150601544T896515lib5747nextseqn0051151bp

1479150601544T896615lib5748nextseqn0051151bp

1479150601544T896715lib5749nextseqn0051151bp

1479150601544T896815lib5750nextseqn0051151bp

1479150601544T896915lib5751nextseqn0051151bp

1479150601544T897115lib5752nextseqn0051151bp

1479150601544T897215lib5753nextseqn0051151bp

1479150601544T897315lib5754nextseqn0051151bp

1479150601544T897415lib5755nextseqn0051151bp

1479150601544T897515lib5756nextseqn0051151bp

1479150601544T897615lib5757nextseqn0051151bp

1479150601544T897715lib5758nextseqn0051151bp

1479150601544T897915lib5759nextseqn0051151bp

1479150601544T898015lib5760nextseqn0051151bp

1479150601544T898115lib5761nextseqn0051151bp

1479150601544T898315lib5762nextseqn0051151bp

1479151773189T1375914lib3391nextseqn0004151bp

1479151773189T1376014lib3392nextseqn0012151bp

1479151773189T1376214lib3393nextseqn0004151bp

1479151773189T1376314lib3394nextseqn0012151bp

1479151773189T1376414lib3395nextseqn0004151bp

1479151773189T1376514lib3396nextseqn0004151bp

1479151773189T1376614lib3397nextseqn0012151bp

1479151773189T1376714lib3398nextseqn0004151bp

1479151773189T1376814lib3399nextseqn0004151bp

1479151773189T1376914lib3400nextseqn0004151bp

1479151773189T1377014lib3401nextseqn0004151bp

1479151773189T1377114lib3402nextseqn0004151bp

1479151773189T1377214lib3403nextseqn0004151bp

1479151773189T1377414lib3405nextseqn0012151bp

1479151773189T1377614lib3407nextseqn0004151bp

1479151773189T1377714lib3408nextseqn0004151bp

1479151773189T1377814lib3409nextseqn0004151bp

1479151773189T1377914lib3410nextseqn0004151bp

1479151773189T1378114lib3412nextseqn0004151bp

1479151773189T1378214lib3413nextseqn0004151bp

1479151773189T1378314lib3414nextseqn0004151bp

1479151773189T1378414lib3415nextseqn0004151bp

1479151773189T1378514lib3416nextseqn0004151bp

1479151773189T1378614lib3417nextseqn0004151bp

1479151773189T1378814lib3419nextseqn0004151bp

1479151773189T1378914lib3420nextseqn0004151bp

1479151773189T1379014lib3421nextseqn0012151bp

1479151773189T1379114lib3422nextseqn0004151bp

1479151773189T1379214lib3423nextseqn0004151bp

1479151773189T1379314lib3424nextseqn0012151bp

1479151773189T898415lib5763nextseqn0051151bp

1479151773189T898515lib5764nextseqn0051151bp

1479151773189T898615lib5765nextseqn0051151bp

1479151773189T898715lib5766nextseqn0051151bp

1479151773189T898815lib5767nextseqn0051151bp

1479151773189T898915lib5768nextseqn0051151bp

1479151773189T899015lib5769nextseqn0051151bp

1479151773189T899115lib5770nextseqn0051151bp

1479151773189T899215lib5771nextseqn0051151bp

1479153938227T1379514lib3426nextseqn0004151bp

1479153938227T1379614lib3427nextseqn0004151bp

1479153938227T1379714lib3428nextseqn0004151bp

1479153938227T1379814lib3429nextseqn0004151bp

1479153938227T1379914lib3430nextseqn0004151bp

1479153938227T1380014lib3431nextseqn0004151bp

1479153938227T1380214lib3433nextseqn0004151bp

1479153938227T1380314lib3434nextseqn0004151bp

1479153938227T1380414lib3435nextseqn0004151bp

1479153938227T1380514lib3436nextseqn0004151bp

1479153938227T1380614lib3971miseqr0139301bp

1479153938227T1380614lib3971miseqr0140301bp

1479153938227T1380714lib3437nextseqn0004151bp

1479153938227T1380814lib3438nextseqn0004151bp

1479153938227T1380914lib3439nextseqn0005151bp

1479153938227T1416615lib6732nextseqn0066151bp

1479153938227T1416915lib6736nextseqn0069151bp

1479153938227T1417015lib6737nextseqn0069151bp

1479153938227T1417115lib6743nextseqn0069151bp

1479153938227T1417515lib6734nextseqn0069151bp

1479153938227T1418315lib6740nextseqn0069151bp

1479153938227T1418415lib6742nextseqn0069151bp

1479153938227T1418515lib6741nextseqn0069151bp

1479153938227T1418915lib6735nextseqn0070151bp

1479153938227T1419115lib6738nextseqn0069151bp

1479153938227T1419315lib6739nextseqn0069151bp

1479153938227T1419414lib3440nextseqn0004151bp

1479153938227T1419514lib3441nextseqn0004151bp

1479153938227T1419614lib3442nextseqn0004151bp

1479153938227T1419615lib6733nextseqn0070151bp

1479153938227T1419714lib3443nextseqn0004151bp

1479153938227T1419814lib3444nextseqn0004151bp

1479153938227T1420014lib3445nextseqn0004151bp

1479153938227T1420214lib3447nextseqn0004151bp

1479153938227T1420414lib3449nextseqn0004151bp

1479153938227T1420514lib3450nextseqn0004151bp

1479153938227T1420614lib3972miseqr0139301bp

1479153938227T1420614lib3972miseqr0140301bp

1479153938227T1421014lib3451nextseqn0004151bp

1479153938227T1421114lib3452nextseqn0004151bp

1479153938227T1421214lib3453nextseqn0004151bp

1479159825672T1421414lib3628nextseqn0009151bp

1479159825672T1421514lib3629nextseqn0009151bp

1479159825672T1507814lib3454nextseqn0004151bp

1479159825672T1508314lib3973miseqr0139301bp

1479159825672T1508314lib3973miseqr0140301bp

1479159825672T1509014lib3456nextseqn0004151bp

1479159825672T1509314lib3974miseqr0139301bp

1479159825672T1509414lib3457nextseqn0004151bp

1479159825672T1509614lib3975miseqr0139301bp

1479159825672T1510814lib5080nextseqn0035151bp

1479159825672T1510915lib4765nextseqn0028151bp

1479159825672T1511014lib3643nextseqn0009151bp

1479159825672T1511415lib4567nextseqn0024151bp

1479159825672T1511514lib3657nextseqn0012151bp

1479159825672T1511614lib3645nextseqn0009151bp

1479159825672T1511714lib3658nextseqn0009151bp

1479159825672T1511814lib3970miseqr0139301bp

1479159825672T1512014lib3655nextseqn0009151bp

1479159825672T1512115lib4568nextseqn0024151bp

1479159825672T1512214lib3460nextseqn0005151bp

1479159825672T1548115lib6745nextseqn0069151bp

1479165139537T1548515lib6744nextseqn0066151bp

1479165139537T1548615lib6750nextseqn0066151bp

1479165139537T1549715lib6751nextseqn0070151bp

1479165139537T1550115lib6752nextseqn0070151bp

1479165139537T1550215lib6755nextseqn0069151bp

1479165139537T1551315lib6753nextseqn0069151bp

1479165139537T1552615lib6747nextseqn0066151bp

1479165139537T1552715lib6748nextseqn0066151bp

1479165139537T1552815lib6749nextseqn0066151bp

1479165139537T1552915lib6746nextseqn0066151bp

1479165139537T1686215lib6790nextseqn0069151bp

1479165139537T1686315lib6791nextseqn0066151bp

1479165139537T1686415lib6792nextseqn0066151bp

1479165139537T1686515lib6793nextseqn0066151bp

1479165139537T1686615lib6794nextseqn0069151bp

1479165139537T1686715lib6795nextseqn0070151bp

1479165139537T1686815lib6796nextseqn0066151bp

1479165139537T1686915lib6797nextseqn0069151bp

1479165139537T1687015lib6798nextseqn0070151bp

1479165139537T1687115lib6799nextseqn0066151bp

1479165139537T1687215lib6800nextseqn0070151bp

1479165139537T1687315lib6801nextseqn0070151bp

1479165139537T1687415lib6802nextseqn0069151bp

1479165139537T1687515lib6803nextseqn0069151bp

1479165139537T1687615lib6804nextseqn0069151bp

1479166472634T1687715lib6805nextseqn0070151bp

1479166472634T1687815lib6806nextseqn0066151bp

1479166472634T1687915lib6807nextseqn0066151bp

1479166472634T1688015lib6808nextseqn0066151bp

1479166472634T1688115lib6809nextseqn0066151bp

1479166472634T1688215lib6810nextseqn0066151bp

1479166472634T1688315lib6811nextseqn0070151bp

1479166472634T1688415lib6812nextseqn0066151bp

1479166472634T1688515lib6813nextseqn0066151bp

1479166472634T1688615lib6814nextseqn0066151bp

1479166472634T1688715lib6815nextseqn0066151bp

1479166472634T1688815lib6816nextseqn0066151bp

1479166472634T1688915lib6817nextseqn0069151bp

1479166472634T1689215lib6820nextseqn0069151bp

1479166472634T1689315lib6821nextseqn0069151bp

1479166472634T1689415lib6822nextseqn0069151bp

1479166472634T1689515lib6900nextseqn0070151bp

1479166472634T1689615lib6901nextseqn0070151bp

1479166472634T1689715lib6902nextseqn0070151bp

1479166472634T1689815lib6903nextseqn0070151bp

1479166472634T1689915lib6904nextseqn0070151bp

1479166472634T1690015lib6905nextseqn0070151bp

1481299601832TSRR1186316

1481299601832TSRR1186993

1481299601832TSRR1187001

1481299601832TSRR1187028

1481299601832TSRR1187037

1481299601832TSRR1187076

1481299601832TSRR1187082

1481299601832TSRR1187085

1481299601832TSRR1187086

1481299601832TSRR1187087

1481299601832TSRR1187088

1481299601832TSRR1187089

1481299601832TSRR1187181

1481299601832TSRR1187183

1481299601832TSRR1187184

1481299601832TSRR1187186

1481299601832TSRR1187192

1481299601832TSRR1187195

1481299601832TSRR1187196

1481299601832TSRR1187251

1481299601832TSRR1187252

1481299601832TSRR1187295

1481299601832TSRR1187297

1481299601832TSRR1187380

1481299601832TSRR1187393

1481299601832TSRR1187423

1481299601832TSRR1187442

1481299601832TSRR1187576

1481299601832TSRR1187598

1481299601832TSRR1187618

1481299601832TSRR1187619

1481299601832TSRR1187620

1481299601832TSRR1187627

1481299601832TSRR1187628

1481299601832TSRR1187629

1481299601832TSRR1187630

1481299601832TSRR1187631

1481299601832TSRR1187633

1481299601832TSRR1187945

1481299601832TSRR1187946

1481299601832TSRR1187983

1481299601832TSRR1188082

1481299601832TSRR1188083

1481299601832TSRR1188084

1481299601832TSRR1188085

1481299601832TSRR1188086

1481299601832TSRR1188087

1481299601832TSRR1188119

1481299601832TSRR1188121

1481299601832TSRR1188127

1481299601832TSRR1188130

1481299601832TSRR1188131

1481299601832TSRR1188133

1481299601832TSRR1188137

1481299601832TSRR1188138

1481299601832TSRR1188143

1481299601832TSRR1188170

1481299601832TSRR1188174

1481299601832TSRR1188175

1481299601832TSRR1188181

1481303126978TSRR1187088

1481303126978TSRR1187089

1481303126978TSRR1187181

1481303126978TSRR1187183

1481303126978TSRR1187184

1481303126978TSRR1187186

1481303126978TSRR1187192

1481303126978TSRR1187195

1481303126978TSRR1187196

1481303126978TSRR1187251

1481303126978TSRR1187252

1481303126978TSRR1187295

1481303126978TSRR1187297

1481303126978TSRR1187380

1481303126978TSRR1187393

1481303126978TSRR1187423

1481303126978TSRR1187442

1481303126978TSRR1187576

1481303126978TSRR1187598

1481303126978TSRR1187618

1481303126978TSRR1187619

1481303126978TSRR1187620

1481303126978TSRR1187627

1481303126978TSRR1187628

1481303126978TSRR1187629

1481303126978TSRR1187630

1481303126978TSRR1187631

1481303126978TSRR1187633

1481303126978TSRR1187945

1481303126978TSRR1187946

1481303126978TSRR1187983

1481303126978TSRR1188082

1481303126978TSRR1188083

1481303126978TSRR1188084

1481303126978TSRR1188085

1481303126978TSRR1188086

1481303126978TSRR1188087

1481303126978TSRR1188119

1481303126978TSRR1188121

1481303126978TSRR1188127

1481303126978TSRR1188130

1481303126978TSRR1188131

1481303126978TSRR1188133

1481303126978TSRR1188137

1481303126978TSRR1188138

1481303126978TSRR1188143

1481303126978TSRR1188170

1481303126978TSRR1188174

1481303126978TSRR1188175

1481303126978TSRR1188181

1481303126978TSRR1188183

1481303126978TSRR1188184

1481303126978TSRR1188188

1481303126978TSRR1188220

1481303126978TSRR1188259

1481303126978TSRR1188284

1481303126978TSRR1188339

1481303126978TSRR1188341

1481303126978TSRR1188343

1481303126978TSRR1188359

1481303126978TSRR1188439

1481303126978TSRR1188459

1481303126978TSRR1188479

1481303126978TSRR1188486

1481303126978TSRR1188487

1481303126978TSRR1188492

1481303126978TSRR1190434

1481303126978TSRR1190442

1481303126978TSRR1190470

1481303126978TSRR1190473

1481303126978TSRR1190474

1481303126978TSRR1190475

1481303126978TSRR1190477

1481303126978TSRR1190478

1481303126978TSRR1190480

1481303126978TSRR1191277

1481303126978TSRR1191298

1481303126978TSRR1191488

1481303126978TSRR1191489

1481303126978TSRR1191546

1481303126978TSRR1191547

1481303126978TSRR1191666

1481303126978TSRR1191727

1481303126978TSRR1200251

1481303126978TSRR1367198

1481303126978TSRR1367199

1481303126978TSRR1367200

1481303126978TSRR1640242

1481303126978TSRR1640246

1481303126978TSRR1640250

1481303126978TSRR1640253

1481303126978TSRR1640254

1481303126978TSRR1640266

1481303126978TSRR1640273

1481303126978TSRR1640287

1481303126978TSRR1640289

1481303126978TSRR1640291

1481303126978TSRR1640293

1481303126978TSRR1640294

1481303126978TSRR1640295

1481303126978TSRR1640316

1481303126978TSRR1640321

1481303126978TSRR1640326

1481303126978TSRR1640327

1481303126978TSRR1640334

1481303126978TSRR1640335

1481303126978TSRR1640336

1481303126978TSRR1640338

1481303126978TSRR1640340

1481303126978TSRR1640342

1481303126978TSRR1640343

1481303126978TSRR1640345

1481303126978TSRR1640346

1481303126978TSRR1640347

1481303126978TSRR1640348

1481303126978TSRR1640349

1481303126978TSRR1640539

1481303126978TSRR1640540

1481303126978TSRR1640541

1481303126978TSRR1640542

1481303126978TSRR1640543

1481303126978TSRR1640547

1481303126978TSRR1640549

1481303126978TSRR1640551

1481303126978TSRR1640562

1481303126978TSRR1640563

1481303126978TSRR1640564

1481303126978TSRR1640565

1481303126978TSRR1640566

1481303126978TSRR1640567

1481303126978TSRR1640569

1481303126978TSRR1640570

1481303126978TSRR1640571

1481303126978TSRR1640574

1481303126978TSRR1640696

1481303126978TSRR1640697

1481303126978TSRR1640701

1481303126978TSRR1640706

1481303126978TSRR1640707

1481303126978TSRR1640708

1481303126978TSRR1640710

1481303126978TSRR1640711

1481303126978TSRR1640712

1481303126978TSRR1640714

1481303126978TSRR1640749

1493677705795T464616lib9483nextseqn0128151bp

1493677705795T464716lib9484nextseqn0128151bp

1493677705795T467116lib9486nextseqn0128151bp

1493678103259T464616lib9483nextseqn0128151bp

1493678103259T464716lib9484nextseqn0128151bp

1493678103259T464816lib9485nextseqn0128151bp

1493678103259T464916lib9470nextseqn0128151bp

1493678103259T465016lib9471nextseqn0128151bp

1493678103259T465116lib9472nextseqn0128151bp

1493678103259T465216lib9473nextseqn0128151bp

1493678103259T465316lib9474nextseqn0128151bp

1493678103259T465416lib9475nextseqn0128151bp

1493678103259T465516lib9879nextseqn0135151bp

1493678103259T465616lib9880nextseqn0135151bp

1493678103259T465716lib9881nextseqn0135151bp

1493678103259T465816lib9882nextseqn0135151bp

1493678103259T465916lib9883nextseqn0135151bp

1493678103259T466016lib9884nextseqn0135151bp

1493678103259T466116lib9885nextseqn0136151bp

1493678103259T466216lib9886nextseqn0136151bp

1493678103259T466316lib9887nextseqn0136151bp

1493678103259T466416lib9888nextseqn0136151bp

1493678103259T466516lib9889nextseqn0136151bp

1493678103259T466716lib9476nextseqn0128151bp

1493678103259T466816lib9477nextseqn0128151bp

1493678103259T466916lib9478nextseqn0128151bp

1493678103259T467016lib9479nextseqn0128151bp

1493678103259T467116lib9486nextseqn0128151bp

1493679460066T467216lib9487nextseqn0128151bp

1493679460066T467316lib9488nextseqn0128151bp

1493679460066T556016lib8231nextseqn0107151bp

1493679460066T556316lib8234nextseqn0106151bp

1493679460066T556816lib8217nextseqn0106151bp

1493679460066T557116lib8372nextseqn0106151bp

1493679460066T557316lib9892nextseqn0136151bp

1493679460066T557416lib9893nextseqn0136151bp

1493679460066T557516lib9894nextseqn0136151bp

1493679460066T557716lib9896nextseqn0135151bp

1493679460066T557816lib9897nextseqn0135151bp

1493679460066T557916lib9898nextseqn0135151bp

1493679460066T558016lib9899nextseqn0135151bp

1493679460066T558116lib9900nextseqn0135151bp

1493679460066T558316lib9902nextseqn0135151bp

1493679460066T558516lib9904nextseqn0135151bp

1493679460066T558616lib9905nextseqn0135151bp

1493679460066T558816lib9480nextseqn0128151bp

1493679460066T558916lib9481nextseqn0128151bp

1493679460066T559016lib8222nextseqn0106151bp

1493679460066T559616lib8225nextseqn0106151bp

1493679460066T559716lib9482nextseqn0128151bp

1493679460066T559816lib8238nextseqn0107151bp

1493679460066T559916lib8239nextseqn0106151bp

1493680648850T560016lib8240nextseqn0106151bp

1493680648850T560116lib8241nextseqn0106151bp

1493680648850T560216lib8242nextseqn0107151bp

1493680648850T560816lib8228nextseqn0106151bp

1493680648850T561716lib8269nextseqn0106151bp

1493680648850T561816lib8270nextseqn0107151bp

1493680648850T561916lib8229nextseqn0107151bp

1493680648850TF01015537lib9098nextseqn0116151bp

1493680648850TF01015642lib9099nextseqn0116151bp

1493680648850TF01015643lib9100nextseqn0116151bp

1493680648850TF01015645lib9101nextseqn0116151bp

1493680648850TF01015705lib9102nextseqn0116151bp

1493680648850TF01015707lib9103nextseqn0116151bp

1493680648850TF01015709lib9104nextseqn0116151bp

1493680648850TF01015796lib9105nextseqn0116151bp

1493680648850TF01015796lib9105nextseqn0119151bp

1493680648850TF01015828lib9106nextseqn0116151bp

1493680648850TF01015828lib9106nextseqn0119151bp

1493680648850TF01015933lib9107nextseqn0116151bp

1493680648850TF01015933lib9107nextseqn0128151bp

1493680648850TF01015964lib9108nextseqn0116151bp

1493680648850TF01015964lib9108nextseqn0119151bp

1493680648850TF01015966lib9109nextseqn0116151bp

1493680648850TF01015966lib9109nextseqn0119151bp

1493743704306TF01015967lib9110nextseqn0116151bp

1493743704306TF01015967lib9110nextseqn0128151bp

1493743704306TF01015968lib9111nextseqn0116151bp

1493743704306TF01015968lib9111nextseqn0119151bp

1493743704306TF01015970lib9112nextseqn0116151bp

1493743704306TF01015970lib9112nextseqn0119151bp

1493743704306TF01015970lib9112nextseqn0128151bp

1493743704306TF01016015lib9113nextseqn0116151bp

1493743704306TF01016016lib9114nextseqn0119151bp

1493743704306TF01016084lib9115nextseqn0119151bp

1493743704306TF01016087lib9116nextseqn0119151bp

1493743704306TF01016087lib9116nextseqn0128151bp

1493743704306TF01016089lib9117nextseqn0116151bp

1493743704306TF01016094lib9118nextseqn0119151bp

1493743704306TF01016220lib9119nextseqn0119151bp

1493743704306TF01016276lib9120nextseqn0119151bp

1493743704306TF01016284lib9121nextseqn0119151bp

1493743704306TF01016666lib9124nextseqn0116151bp

1493743704306TF01670004lib9125nextseqn0116151bp

1493743704306TF01670013lib9126nextseqn0116151bp

1493743704306TF01670015lib9127nextseqn0116151bp

1493743704306TF01670021lib9128nextseqn0116151bp

1495492153992TF01670021lib9128nextseqn0119151bp

1495492153992TF01670022lib9129nextseqn0116151bp

1495492153992TF01670023lib9130nextseqn0116151bp

1495492153992TF01670024lib9131nextseqn0116151bp

1495492153992TF01670024lib9131nextseqn0119151bp

1495492153992TF01670025lib9132nextseqn0116151bp

1495492153992TF01670027lib9133nextseqn0116151bp

1495492153992TF01670028lib9134nextseqn0116151bp

1495492153992TF01670030lib9135nextseqn0119151bp

1495492153992TF01670031lib9136nextseqn0119151bp

1495492153992TF01670032lib9137nextseqn0119151bp

1495492153992TF01670033lib9138nextseqn0119151bp

1495492153992TF01670034lib9139nextseqn0116151bp

1495492153992TF01670035lib9140nextseqn0116151bp

1495492153992TF01670036lib9141nextseqn0116151bp

1495492153992TF01670036lib9141nextseqn0119151bp

1495492153992TF01670038lib9142nextseqn0116151bp

1495492153992TF01670038lib9142nextseqn0119151bp

1495492153992TF01670038lib9142nextseqn0128151bp

1495492153992TF01670039lib9143nextseqn0116151bp

1495492153992TF01670040lib9144nextseqn0119151bp

1495492153992TF01670041lib9145nextseqn0128151bp

1495492153992TF01670042lib9146nextseqn0128151bp

1495492153992TF01670043lib9147nextseqn0128151bp

1495492153992TF01670046lib9148nextseqn0128151bp

1495493001541TF01670047lib9149nextseqn0116151bp

1702

1719

1726

1727

1728

1738

1739

1741

1742

1744

1745

1753

1756

1759

1760

1761

1762

1763

1767

1768

1770

1774

1778

1780

1782

1784

1785

1786

1788

1789

1791

1793

1795

1796

1803

1807

1808

1809

1810

1815

1821

1824

1828

1830

1831

1832

1833

1834

1836

1837

1841

1851

1852

1853

1854

98R454

9910364

99R1043

99R1083

99R141

99R545

99R576

99R719

99R862

99R887

99R890

AFB0600004110

AFB0700001150

AFB0700001273

AFB0700001685

AFB0700002740

AFB0700003121

AFB0800000210

AFB0800000295

AFB0800000324

AFB0800000532

AFB0800001872

AFB0900001175

ERR015614

ERR015615

ERR023729

ERR023731

ERR023732

ERR023737

ERR023738

ERR023739

ERR023740

ERR023743

ERR023745

ERR023746

ERR023747

ERR023748

ERR023752

ERR023754

ERR023757

ERR023758

ERR023759

ERR023760

ERR023761

ERR023762

ERR023763

ERR023764

ERR024340

ERR024341

ERR024342

ERR024343

ERR024344

ERR024345

ERR024346

ERR024347

ERR025414

ERR025415

ERR025416

ERR025417

ERR025418

ERR025419

ERR025420

ERR025421

ERR025422

ERR025423

ERR025424

ERR025426

ERR025427

ERR025428

ERR025429

ERR025430

ERR025431

ERR025432

ERR025433

ERR025434

ERR025435

ERR025436

ERR025438

ERR025439

ERR025440

ERR025441

ERR025442

ERR025443

ERR025444

ERR025445

ERR025446

ERR025447

ERR025448

ERR025450

ERR025451

ERR025452

ERR025453

ERR025454

ERR025455

ERR025456

ERR025457

ERR025458

ERR025459

ERR025460

ERR025833

ERR025834

ERR025835

ERR025836

ERR025837

ERR025838

ERR025839

ERR025840

ERR025842

ERR025843

ERR025844

ERR025846

ERR025847

ERR025848

ERR026472

ERR026473

ERR026474

ERR026475

ERR026476

ERR026477

ERR026478

ERR026479

ERR026480

ERR026481

ERR026482

ERR028607

ERR028608

ERR028609

ERR028610

ERR028611

ERR028612

ERR028613

ERR028615

ERR028616

ERR028617

ERR028619

ERR028620

ERR028621

ERR029201

ERR029202

ERR029203

ERR029205

ERR029206

ERR029207

ERR029209

ERR029210

ERR029211

ERR036186

ERR036187

ERR036188

ERR036189

ERR036190

ERR036192

ERR036193

ERR036195

ERR036196

ERR036197

ERR036199

ERR036201

ERR036202

ERR036203

ERR036204

ERR036205

ERR036206

ERR036208

ERR036209

ERR036210

ERR036212

ERR036213

ERR036214

ERR036216

ERR036217

ERR036218

ERR036219

ERR036220

ERR036221

ERR036222

ERR036223

ERR036226

ERR036227

ERR036228

ERR036229

ERR036230

ERR036231

ERR036232

ERR036234

ERR036235

ERR036236

ERR036238

ERR036239

ERR036240

ERR036241

ERR036243

ERR036244

ERR036245

ERR036246

ERR036247

ERR036248

ERR036249

ERR037467

ERR037468

ERR037470

ERR037471

ERR037472

ERR037473

ERR037474

ERR037475

ERR037476

ERR037477

ERR037478

ERR037479

ERR037480

ERR037481

ERR037482

ERR037483

ERR037484

ERR037485

ERR037486

ERR037487

ERR037488

ERR037489

ERR037490

ERR037491

ERR037493

ERR037495

ERR037496

ERR037497

ERR037498

ERR037499

ERR037500

ERR037502

ERR037503

ERR037505

ERR037507

ERR037508

ERR037509

ERR037510

ERR037512

ERR037514

ERR037515

ERR037516

ERR037517

ERR037518

ERR037519

ERR037520

ERR037521

ERR037522

ERR037523

ERR037524

ERR037525

ERR037526

ERR037527

ERR037528

ERR037530

ERR037531

ERR037532

ERR037533

ERR037534

ERR037536

ERR037537

ERR037538

ERR037539

ERR037540

ERR037541

ERR037542

ERR037543

ERR037544

ERR037545

ERR037546

ERR037548

ERR037549

ERR037551

ERR037553

ERR037554

ERR037555

ERR038254

ERR038255

ERR038256

ERR038257

ERR038258

ERR038259

ERR038260

ERR038261

ERR038262

ERR038263

ERR038264

ERR038265

ERR038266

ERR038269

ERR038270

ERR038271

ERR038272

ERR038273

ERR038274

ERR038275

ERR038276

ERR038277

ERR038278

ERR038279

ERR038280

ERR038281

ERR038282

ERR038283

ERR038284

ERR038285

ERR038286

ERR038288

ERR038290

ERR038291

ERR038292

ERR038293

ERR038294

ERR038295

ERR038296

ERR038297

ERR038298

ERR038299

ERR038300

ERR038736

ERR038738

ERR038739

ERR038740

ERR038741

ERR038742

ERR038743

ERR038744

ERR038746

ERR038747

ERR038748

ERR038749

ERR038750

ERR038751

ERR038752

ERR038753

ERR038754

ERR038755

ERR039323

ERR039324

ERR039325

ERR039326

ERR039327

ERR039328

ERR039329

ERR039330

ERR039331

ERR039332

ERR039333

ERR039334

ERR039335

ERR039336

ERR039337

ERR039338

ERR039339

ERR039340

ERR039341

ERR039342

ERR039343

ERR039344

ERR039345

ERR039346

ERR040086

ERR040087

ERR040088

ERR040089

ERR040090

ERR040091

ERR040093

ERR040094

ERR040096

ERR040097

ERR040098

ERR040099

ERR040100

ERR040101

ERR040102

ERR040103

ERR040104

ERR040105

ERR040106

ERR040107

ERR040108

ERR040109

ERR040112

ERR040113

ERR040114

ERR040115

ERR040116

ERR040117

ERR040118

ERR040119

ERR040120

ERR040121

ERR040122

ERR040123

ERR040124

ERR040125

ERR040126

ERR040127

ERR040128

ERR040129

ERR040130

ERR040131

ERR040132

ERR040133

ERR040134

ERR040135

ERR040137

ERR040138

ERR040139

ERR040140

ERR040141

ERR040142

ERR046729

ERR046730

ERR046732

ERR046733

ERR046734

ERR046735

ERR046736

ERR046737

ERR046738

ERR046739

ERR046741

ERR046743

ERR046744

ERR046745

ERR046746

ERR046747

ERR046748

ERR046749

ERR046751

ERR046752

ERR046753

ERR046754

ERR046755

ERR046756

ERR046758

ERR046759

ERR046760

ERR046761

ERR046762

ERR046763

ERR046764

ERR046765

ERR046766

ERR046767

ERR046768

ERR046769

ERR046770

ERR046771

ERR046772

ERR046773

ERR046775

ERR046776

ERR046777

ERR046778

ERR046779

ERR046780

ERR046781

ERR046782

ERR046783

ERR046784

ERR046785

ERR046786

ERR046787

ERR046788

ERR046789

ERR046790

ERR046791

ERR046792

ERR046793

ERR046794

ERR046795

ERR046796

ERR046797

ERR046798

ERR046799

ERR046800

ERR046819

ERR046820

ERR046821

ERR046822

ERR046823

ERR046824

ERR046825

ERR046831

ERR046832

ERR046833

ERR046834

ERR046836

ERR046837

ERR046838

ERR046839

ERR046840

ERR046841

ERR046842

ERR046843

ERR046844

ERR046845

ERR046846

ERR046847

ERR046848

ERR046849

ERR046850

ERR046851

ERR046852

ERR046853

ERR046854

ERR046855

ERR046856

ERR046857

ERR046858

ERR046859

ERR046860

ERR046861

ERR046862

ERR046863

ERR046864

ERR046865

ERR046866

ERR046867

ERR046868

ERR046869

ERR046870

ERR046871

ERR046872

ERR046873

ERR046874

ERR046875

ERR046876

ERR046877

ERR046878

ERR046879

ERR046880

ERR046882

ERR046883

ERR046884

ERR046885

ERR046887

ERR046888

ERR046889

ERR046890

ERR046891

ERR046892

ERR046893

ERR046894

ERR046895

ERR046897

ERR046898

ERR046900

ERR046901

ERR046903

ERR046904

ERR046905

ERR046906

ERR046907

ERR046908

ERR046910

ERR046911

ERR046912

ERR046913

ERR046914

ERR046915

ERR046916

ERR046917

ERR046918

ERR046919

ERR046920

ERR046921

ERR046922

ERR046923

ERR046924

ERR046925

ERR046926

ERR046927

ERR046928

ERR046929

ERR046930

ERR046932

ERR046933

ERR046934

ERR046935

ERR046936

ERR046937

ERR046938

ERR046939

ERR046940

ERR046941

ERR046942

ERR046943

ERR046945

ERR046946

ERR046947

ERR046948

ERR046949

ERR046950

ERR046951

ERR046952

ERR046953

ERR046954

ERR046957

ERR046958

ERR046959

ERR046960

ERR046961

ERR046962

ERR046963

ERR046964

ERR046965

ERR046966

ERR046967

ERR046968

ERR046969

ERR046970

ERR046971

ERR046972

ERR046974

ERR046975

ERR046980

ERR046981

ERR046982

ERR046983

ERR046984

ERR046986

ERR046988

ERR046989

ERR046990

ERR046991

ERR046992

ERR046993

ERR046994

ERR046995

ERR046996

ERR046997

ERR046998

ERR046999

ERR047000

ERR047001

ERR047002

ERR047003

ERR047004

ERR047005

ERR047006

ERR047007

ERR047008

ERR047009

ERR047010

ERR047011

ERR047012

ERR047013

ERR047014

ERR047016

ERR067576

ERR067577

ERR067578

ERR067579

ERR067580

ERR067581

ERR067582

ERR067583

ERR067584

ERR067585

ERR067586

ERR067587

ERR067588

ERR067589

ERR067590

ERR067591

ERR067592

ERR067593

ERR067594

ERR067595

ERR067596

ERR067597

ERR067598

ERR067599

ERR067600

ERR067601

ERR067602

ERR067604

ERR067605

ERR067606

ERR067607

ERR067608

ERR067609

ERR067610

ERR067611

ERR067612

ERR067613

ERR067614

ERR067615

ERR067616

ERR067617

ERR067618

ERR067619

ERR067621

ERR067622

ERR067623

ERR067624

ERR067625

ERR067626

ERR067627

ERR067628

ERR067629

ERR067630

ERR067631

ERR067632

ERR067634

ERR067637

ERR067638

ERR067639

ERR067640

ERR067641

ERR067643

ERR067644

ERR067648

ERR067649

ERR067650

ERR067651

ERR067652

ERR067653

ERR067654

ERR067655

ERR067656

ERR067657

ERR067658

ERR067659

ERR067660

ERR067661

ERR067662

ERR067670

ERR067671

ERR067672

ERR067673

ERR067674

ERR067675

ERR067676

ERR067677

ERR067678

ERR067679

ERR067680

ERR067682

ERR067683

ERR067684

ERR067685

ERR067686

ERR067687

ERR067688

ERR067689

ERR067690

ERR067691

ERR067692

ERR067693

ERR067694

ERR067696

ERR067697

ERR067700

ERR067702

ERR067703

ERR067704

ERR067705

ERR067706

ERR067708

ERR067709

ERR067710

ERR067711

ERR067713

ERR067714

ERR067715

ERR067716

ERR067717

ERR067718

ERR067719

ERR067720

ERR067721

ERR067722

ERR067724

ERR067725

ERR067726

ERR067727

ERR067729

ERR067730

ERR067731

ERR067733

ERR067734

ERR067735

ERR067736

ERR067737

ERR067739

ERR067740

ERR067741

ERR067742

ERR067743

ERR067744

ERR067745

ERR067746

ERR067747

ERR067748

ERR067749

ERR067750

ERR067751

ERR067752

ERR067753

ERR067755

ERR067756

ERR067757

ERR067759

ERR067760

ERR067761

ERR067762

ERR067763

ERR067765

ERR067766

ERR067767

ERR072019

ERR072020

ERR072021

ERR072022

ERR072023

ERR072024

ERR072025

ERR072026

ERR072027

ERR072028

ERR072029

ERR072030

ERR072031

ERR072032

ERR072034

ERR072035

ERR072036

ERR072037

ERR072038

ERR072039

ERR072040

ERR072041

ERR072042

ERR072044

ERR072045

ERR072046

ERR072047

ERR072048

ERR072050

ERR072051

ERR072065

ERR072072

ERR072077

ERR072080

ERR072087

ERR072088

ERR072089

ERR072090

ERR072094

ERR072095

ERR072096

ERR108420

ERR108423

ERR108425

ERR108426

ERR108427

ERR108428

ERR108429

ERR108430

ERR108432

ERR108433

ERR108434

ERR108435

ERR108436

ERR108437

ERR108439

ERR108440

ERR108441

ERR108442

ERR108443

ERR108444

ERR108445

ERR108446

ERR108448

ERR108449

ERR108451

ERR108452

ERR108453

ERR108454

ERR108455

ERR108456

ERR108457

ERR108458

ERR108459

ERR108460

ERR108461

ERR108463

ERR108464

ERR108465

ERR108466

ERR108467

ERR108468

ERR108469

ERR108470

ERR108472

ERR108473

ERR108474

ERR108475

ERR108477

ERR108479

ERR108480

ERR108481

ERR108482

ERR108483

ERR108485

ERR108486

ERR108487

ERR108488

ERR108489

ERR108490

ERR108491

ERR108492

ERR108493

ERR108494

ERR108495

ERR108497

ERR108498

ERR108499

ERR108500

ERR108501

ERR108502

ERR108503

ERR108504

ERR108505

ERR108506

ERR108507

ERR108508

ERR108509

ERR108511

ERR108513

ERR108514

ERR108515

ERR117449

ERR117450

ERR117451

ERR117453

ERR117454

ERR117455

ERR117456

ERR117457

ERR117458

ERR117459

ERR117460

ERR117462

ERR117463

ERR117465

ERR117466

ERR117467

ERR117468

ERR117469

ERR117470

ERR124634

ERR124635

ERR124637

ERR124638

ERR124639

ERR124640

ERR124641

ERR124643

ERR124644

ERR124645

ERR124646

ERR124647

ERR124648

ERR124649

ERR124650

ERR125598

ERR125599

ERR125600

ERR125601

ERR125602

ERR125603

ERR125604

ERR125605

ERR125606

ERR125607

ERR125608

ERR125609

ERR125610

ERR125611

ERR125612

ERR125613

ERR125614

ERR125615

ERR125616

ERR125617

ERR125618

ERR125619

ERR125620

ERR125621

ERR125622

ERR125623

ERR125624

ERR125625

ERR125626

ERR125627

ERR125628

ERR126598

ERR126599

ERR126601

ERR126602

ERR126604

ERR126605

ERR126606

ERR126607

ERR126609

ERR126610

ERR126612

ERR126613

ERR126615

ERR126616

ERR126617

ERR126619

ERR126620

ERR126621

ERR126622

ERR126623

ERR126624

ERR126625

ERR126626

ERR126627

ERR126628

ERR126629

ERR126630

ERR126631

ERR126632

ERR126633

ERR126634

ERR126635

ERR126636

ERR126637

ERR126638

ERR126640

ERR126642

ERR126643

ERR126644

ERR133798

ERR133799

ERR133800

ERR133801

ERR133802

ERR133803

ERR133804

ERR133805

ERR133806

ERR133807

ERR133809

ERR133810

ERR133811

ERR133812

ERR133813

ERR133814

ERR133815

ERR133817

ERR133818

ERR133819

ERR133820

ERR133822

ERR133823

ERR133824

ERR133826

ERR133827

ERR133828

ERR133829

ERR133830

ERR133832

ERR133833

ERR133835

ERR133836

ERR133837

ERR133838

ERR133839

ERR133840

ERR133841

ERR133843

ERR133844

ERR133845

ERR133846

ERR133847

ERR133848

ERR133849

ERR133850

ERR133851

ERR133852

ERR133853

ERR133854

ERR133855

ERR133856

ERR133857

ERR133858

ERR133859

ERR133860

ERR133861

ERR133862

ERR133864

ERR133865

ERR133866

ERR133867

ERR133868

ERR133871

ERR133872

ERR133873

ERR133874

ERR133875

ERR133876

ERR133877

ERR133879

ERR133880

ERR133881

ERR133882

ERR133883

ERR133884

ERR133885

ERR133886

ERR133888

ERR133889

ERR133890

ERR133891

ERR133892

ERR133893

ERR133895

ERR133896

ERR133897

ERR133898

ERR133899

ERR133900

ERR133901

ERR133902

ERR133903

ERR133904

ERR133905

ERR133906

ERR133907

ERR133908

ERR133909

ERR133910

ERR133911

ERR133913

ERR133914

ERR133915

ERR133916

ERR133917

ERR133918

ERR133919

ERR133920

ERR133921

ERR133922

ERR133924

ERR133925

ERR133926

ERR133929

ERR133930

ERR133931

ERR133932

ERR133933

ERR133934

ERR133935

ERR133938

ERR133939

ERR133941

ERR133942

ERR133943

ERR133944

ERR133945

ERR133947

ERR133948

ERR133949

ERR133951

ERR133952

ERR133954

ERR133955

ERR133956

ERR133957

ERR133958

ERR133959

ERR133960

ERR133961

ERR133962

ERR133963

ERR133964

ERR133965

ERR133966

ERR133967

ERR133968

ERR133969

ERR133970

ERR133971

ERR133973

ERR133974

ERR133975

ERR133976

ERR133977

ERR133978

ERR133979

ERR133980

ERR133981

ERR133982

ERR133983

ERR133984

ERR133985

ERR133987

ERR133988

ERR133989

ERR137192

ERR137193

ERR137195

ERR137196

ERR137197

ERR137198

ERR137199

ERR137200

ERR137201

ERR137203

ERR137204

ERR137206

ERR137207

ERR137208

ERR137209

ERR137210

ERR137211

ERR137213

ERR137214

ERR137215

ERR137216

ERR137217

ERR137218

ERR137219

ERR137220

ERR137221

ERR137222

ERR137223

ERR137225

ERR137227

ERR137228

ERR137229

ERR137230

ERR137231

ERR137232

ERR137234

ERR137235

ERR137236

ERR137237

ERR137238

ERR137240

ERR137241

ERR137242

ERR137243

ERR137244

ERR137245

ERR137246

ERR137247

ERR137248

ERR137249

ERR137250

ERR137251

ERR137252

ERR137253

ERR137254

ERR137255

ERR137257

ERR137259

ERR137260

ERR137261

ERR137262

ERR137263

ERR137264

ERR137265

ERR137267

ERR137268

ERR137269

ERR137272

ERR137273

ERR137274

ERR137275

ERR137277

ERR137279

ERR137280

ERR137281

ERR137282

ERR137283

ERR137284

ERR137285

ERR144543

ERR144544

ERR144545

ERR144546

ERR144547

ERR144548

ERR144549

ERR144550

ERR144551

ERR144552

ERR144553

ERR144555

ERR144558

ERR144561

ERR144562

ERR144563

ERR144564

ERR144565

ERR144566

ERR144569

ERR144570

ERR144571

ERR144572

ERR144573

ERR144574

ERR144575

ERR144576

ERR144577

ERR144578

ERR144579

ERR144580

ERR144581

ERR144582

ERR144584

ERR144585

ERR144587

ERR144588

ERR144590

ERR144591

ERR144592

ERR144593

ERR144594

ERR144595

ERR144596

ERR144597

ERR144598

ERR144599

ERR144600

ERR144601

ERR144602

ERR144603

ERR144604

ERR144606

ERR144607

ERR144608

ERR144609

ERR144610

ERR144613

ERR144614

ERR144615

ERR144616

ERR144619

ERR144622

ERR144624

ERR144625

ERR144626

ERR144628

ERR144630

ERR144631

ERR144632

ERR144633

ERR144634

ERR144635

ERR144636

ERR158569

ERR158570

ERR158572

ERR158573

ERR158574

ERR158575

ERR158576

ERR158577

ERR158578

ERR158579

ERR158580

ERR158581

ERR158582

ERR158584

ERR158585

ERR158586

ERR158587

ERR158588

ERR158589

ERR158590

ERR158591

ERR158592

ERR158593

ERR158594

ERR158595

ERR158596

ERR158597

ERR158598

ERR158599

ERR158601

ERR158602

ERR158603

ERR158604

ERR158607

ERR158608

ERR158609

ERR158611

ERR158612

ERR158613

ERR158614

ERR158615

ERR158616

ERR161012

ERR161013

ERR161015

ERR161016

ERR161017

ERR161018

ERR161019

ERR161020

ERR161021

ERR161023

ERR161025

ERR161028

ERR161029

ERR161030

ERR161031

ERR161032

ERR161035

ERR161036

ERR161038

ERR161040

ERR161041

ERR161042

ERR161043

ERR161044

ERR161046

ERR161048

ERR161051

ERR161052

ERR161053

ERR161054

ERR161055

ERR161056

ERR161057

ERR161058

ERR161060

ERR161061

ERR161062

ERR161063

ERR161064

ERR161065

ERR161066

ERR161067

ERR161068

ERR161069

ERR161070

ERR161072

ERR161073

ERR161074

ERR161075

ERR161076

ERR161079

ERR161080

ERR161081

ERR161083

ERR161086

ERR161087

ERR161088

ERR161089

ERR161090

ERR161091

ERR161092

ERR161093

ERR161094

ERR161095

ERR161096

ERR161097

ERR161098

ERR161100

ERR161101

ERR161102

ERR161103

ERR161104

ERR161105

ERR161106

ERR161107

ERR161108

ERR161109

ERR161110

ERR161111

ERR161112

ERR161113

ERR161114

ERR161115

ERR161116

ERR161117

ERR161118

ERR161119

ERR161120

ERR161121

ERR161124

ERR161126

ERR161127

ERR161128

ERR161129

ERR161130

ERR161131

ERR161132

ERR161133

ERR161134

ERR161135

ERR161136

ERR161137

ERR161138

ERR161139

ERR161140

ERR161141

ERR161142

ERR161143

ERR161144

ERR161145

ERR161146

ERR161147

ERR161148

ERR161151

ERR161152

ERR161153

ERR161154

ERR161155

ERR161156

ERR161157

ERR161158

ERR161159

ERR161160

ERR161161

ERR161162

ERR161164

ERR161165

ERR161166

ERR161167

ERR161168

ERR161169

ERR161171

ERR161172

ERR161174

ERR161175

ERR161176

ERR161177

ERR161178

ERR161179

ERR161181

ERR161182

ERR161183

ERR161185

ERR161186

ERR161187

ERR161188

ERR161189

ERR161190

ERR161191

ERR161192

ERR161193

ERR161195

ERR161196

ERR161197

ERR161198

ERR161199

ERR161202

ERR161203

ERR163928

ERR163929

ERR163930

ERR163931

ERR163932

ERR163933

ERR163934

ERR163935

ERR163936

ERR163937

ERR163939

ERR163941

ERR163942

ERR163944

ERR163945

ERR163946

ERR163947

ERR163949

ERR163950

ERR163951

ERR163953

ERR163954

ERR163955

ERR163956

ERR163957

ERR163958

ERR163959

ERR163960

ERR163961

ERR163962

ERR163963

ERR163964

ERR163966

ERR163967

ERR163968

ERR163969

ERR163972

ERR163973

ERR163974

ERR163975

ERR163976

ERR163977

ERR163978

ERR163979

ERR163980

ERR163981

ERR163982

ERR163983

ERR163984

ERR163985

ERR163989

ERR163990

ERR163991

ERR163992

ERR163993

ERR163994

ERR163995

ERR163997

ERR163998

ERR163999

ERR164000

ERR164001

ERR164002

ERR164003

ERR164005

ERR164006

ERR164007

ERR164008

ERR164009

ERR164010

ERR164011

ERR164012

ERR164013

ERR164014

ERR164015

ERR164016

ERR164017

ERR164018

ERR164019

ERR164020

ERR164022

ERR164023

ERR176446

ERR176447

ERR176448

ERR176449

ERR176451

ERR176452

ERR176453

ERR176454

ERR176455

ERR176456

ERR176457

ERR176458

ERR176459

ERR176461

ERR176462

ERR176463

ERR176465

ERR176466

ERR176467

ERR176468

ERR176469

ERR176470

ERR176471

ERR176472

ERR176473

ERR176474

ERR176476

ERR176477

ERR176478

ERR176479

ERR176480

ERR176481

ERR176483

ERR176484

ERR176485

ERR176486

ERR176487

ERR176488

ERR176490

ERR176491

ERR176492

ERR176493

ERR176494

ERR176495

ERR176496

ERR176497

ERR176498

ERR176499

ERR176500

ERR176501

ERR176502

ERR176503

ERR176504

ERR176505

ERR176506

ERR176507

ERR176508

ERR176509

ERR176510

ERR176511

ERR176512

ERR176513

ERR176514

ERR176515

ERR176516

ERR176519

ERR176520

ERR176521

ERR176522

ERR176523

ERR176525

ERR176526

ERR176527

ERR176528

ERR176529

ERR176530

ERR176531

ERR176532

ERR176533

ERR176534

ERR176535

ERR176536

ERR176537

ERR176538

ERR176539

ERR176540

ERR176541

ERR176542

ERR176543

ERR176545

ERR176546

ERR176548

ERR176550

ERR176551

ERR176552

ERR176553

ERR176554

ERR176555

ERR176558

ERR176559

ERR176561

ERR176562

ERR176563

ERR176564

ERR176566

ERR176567

ERR176569

ERR176570

ERR176571

ERR176572

ERR176573

ERR176574

ERR176575

ERR176576

ERR176577

ERR176578

ERR176579

ERR176581

ERR176582

ERR176583

ERR176584

ERR176585

ERR176587

ERR176588

ERR176589

ERR176590

ERR176591

ERR176592

ERR176593

ERR176594

ERR176595

ERR176596

ERR176597

ERR176598

ERR176599

ERR176601

ERR176602

ERR176603

ERR176604

ERR176605

ERR176606

ERR176607

ERR176608

ERR176609

ERR176610

ERR176612

ERR176613

ERR176614

ERR176615

ERR176617

ERR176618

ERR176621

ERR176622

ERR176623

ERR176624

ERR176625

ERR176626

ERR176627

ERR176628

ERR176629

ERR176630

ERR176632

ERR176633

ERR176634

ERR176635

ERR176636

ERR176637

ERR176638

ERR176639

ERR176640

ERR176641

ERR176642

ERR176644

ERR176645

ERR176646

ERR176647

ERR176648

ERR176649

ERR176653

ERR176654

ERR176655

ERR176656

ERR176657

ERR176659

ERR176660

ERR176661

ERR176662

ERR176663

ERR176664

ERR176665

ERR176666

ERR176667

ERR176668

ERR176669

ERR176670

ERR176671

ERR176673

ERR176676

ERR176677

ERR176678

ERR176679

ERR176680

ERR176681

ERR176682

ERR176683

ERR176684

ERR176685

ERR176686

ERR176687

ERR176688

ERR176689

ERR176690

ERR176691

ERR176692

ERR176693

ERR176694

ERR176695

ERR176696

ERR176697

ERR176698

ERR176699

ERR176700

ERR176701

ERR176702

ERR176703

ERR176704

ERR176705

ERR176706

ERR176710

ERR176711

ERR176712

ERR176713

ERR176714

ERR176715

ERR176716

ERR176717

ERR176719

ERR176720

ERR176721

ERR176722

ERR176723

ERR176724

ERR176725

ERR176726

ERR176727

ERR176728

ERR176729

ERR176730

ERR176731

ERR176732

ERR176733

ERR176734

ERR176735

ERR176736

ERR176737

ERR176738

ERR176742

ERR176743

ERR176744

ERR176745

ERR176746

ERR176747

ERR176748

ERR176750

ERR176752

ERR176753

ERR176754

ERR176755

ERR176757

ERR176758

ERR176759

ERR176760

ERR176761

ERR176762

ERR176763

ERR176764

ERR176765

ERR176767

ERR176768

ERR176769

ERR176770

ERR176772

ERR176773

ERR176774

ERR176775

ERR176776

ERR176777

ERR176782

ERR176783

ERR176784

ERR176785

ERR176786

ERR176787

ERR176788

ERR176789

ERR176790

ERR176791

ERR176792

ERR176794

ERR176795

ERR176796

ERR176797

ERR176798

ERR176799

ERR176800

ERR176801

ERR176802

ERR176803

ERR176804

ERR176805

ERR176806

ERR176808

ERR176809

ERR176810

ERR176812

ERR176814

ERR176815

ERR176816

ERR176817

ERR176818

ERR176820

ERR176821

ERR176822

ERR176823

ERR176824

ERR176825

ERR176826

ERR176827

ERR176829

ERR181314

ERR181315

ERR181316

ERR181435

ERR181440

ERR181674

ERR181675

ERR181676

ERR181677

ERR181678

ERR181679

ERR181680

ERR181681

ERR181682

ERR181683

ERR181684

ERR181685

ERR181687

ERR181688

ERR181689

ERR181690

ERR181691

ERR181692

ERR181693

ERR181694

ERR181696

ERR181697

ERR181698

ERR181699

ERR181700

ERR181701

ERR181702

ERR181703

ERR181704

ERR181706

ERR181707

ERR181710

ERR181711

ERR181713

ERR181714

ERR181715

ERR181716

ERR181717

ERR181718

ERR181719

ERR181720

ERR181721

ERR181722

ERR181723

ERR181724

ERR181725

ERR181726

ERR181727

ERR181728

ERR181729

ERR181730

ERR181731

ERR181732

ERR181734

ERR181735

ERR181736

ERR181737

ERR181738

ERR181739

ERR181740

ERR181741

ERR181742

ERR181743

ERR181745

ERR181746

ERR181747

ERR181748

ERR181751

ERR181752

ERR181753

ERR181754

ERR181755

ERR181756

ERR181757

ERR181758

ERR181759

ERR181760

ERR181762

ERR181763

ERR181764

ERR181765

ERR181766

ERR181767

ERR181768

ERR181769

ERR181772

ERR181773

ERR181774

ERR181775

ERR181776

ERR181777

ERR181778

ERR181779

ERR181780

ERR181781

ERR181783

ERR181786

ERR181787

ERR181788

ERR181789

ERR181790

ERR181791

ERR181792

ERR181793

ERR181794

ERR181796

ERR181797

ERR181798

ERR181799

ERR181800

ERR181801

ERR181802

ERR181803

ERR181805

ERR181806

ERR181807

ERR181808

ERR181809

ERR181810

ERR181812

ERR181814

ERR181816

ERR181817

ERR181818

ERR181819

ERR181820

ERR181821

ERR181822

ERR181823

ERR181825

ERR181826

ERR181829

ERR181830

ERR181831

ERR181832

ERR181833

ERR181834

ERR181835

ERR181836

ERR181837

ERR181838

ERR181839

ERR181840

ERR181841

ERR181842

ERR181843

ERR181844

ERR181845

ERR181846

ERR181847

ERR181848

ERR181850

ERR181851

ERR181852

ERR181853

ERR181854

ERR181856

ERR181858

ERR181859

ERR181860

ERR181861

ERR181862

ERR181865

ERR181866

ERR181867

ERR181868

ERR181869

ERR181870

ERR181871

ERR181872

ERR181873

ERR181874

ERR181875

ERR181876

ERR181877

ERR181878

ERR181879

ERR181880

ERR181883

ERR181884

ERR181885

ERR181886

ERR181888

ERR181889

ERR181891

ERR181892

ERR181893

ERR181894

ERR181895

ERR181896

ERR181897

ERR181898

ERR181899

ERR181900

ERR181901

ERR181902

ERR181903

ERR181904

ERR181905

ERR181906

ERR181907

ERR181908

ERR181909

ERR181910

ERR181911

ERR181912

ERR181913

ERR181914

ERR181915

ERR181916

ERR181917

ERR181918

ERR181919

ERR181920

ERR181921

ERR181922

ERR181923

ERR181924

ERR181925

ERR181926

ERR181927

ERR181928

ERR181929

ERR181930

ERR181931

ERR181932

ERR181933

ERR181934

ERR181935

ERR181936

ERR181938

ERR181939

ERR181941

ERR181942

ERR181943

ERR181944

ERR181946

ERR181947

ERR181948

ERR181949

ERR181950

ERR181951

ERR181952

ERR181953

ERR181954

ERR181955

ERR181956

ERR181957

ERR181958

ERR181960

ERR181961

ERR181962

ERR181963

ERR181964

ERR181965

ERR181966

ERR181967

ERR181968

ERR181969

ERR181970

ERR181971

ERR181972

ERR181973

ERR181974

ERR181975

ERR181978

ERR181979

ERR181980

ERR181981

ERR181982

ERR181983

ERR181984

ERR181985

ERR181986

ERR181987

ERR181988

ERR181989

ERR181990

ERR181991

ERR181992

ERR181993

ERR181994

ERR181995

ERR181996

ERR181997

ERR181998

ERR181999

ERR182000

ERR182001

ERR182002

ERR182003

ERR182004

ERR182005

ERR182006

ERR182007

ERR182008

ERR182009

ERR182010

ERR182011

ERR182012

ERR182013

ERR182014

ERR182015

ERR182016

ERR182017

ERR182018

ERR182019

ERR182020

ERR182021

ERR182022

ERR182023

ERR182024

ERR182025

ERR182027

ERR182028

ERR182029

ERR182030

ERR182031

ERR182032

ERR182033

ERR182034

ERR182035

ERR182036

ERR182037

ERR182038

ERR182039

ERR182040

ERR182041

ERR182042

ERR182043

ERR182044

ERR182045

ERR182046

ERR182047

ERR182048

ERR182049

ERR182050

ERR182051

ERR182052

ERR182053

ERR182054

ERR182055

ERR182056

ERR182057

ERR190328

ERR190329

ERR190330

ERR190331

ERR190333

ERR190334

ERR190335

ERR190336

ERR190337

ERR190338

ERR190341

ERR190344

ERR190346

ERR190347

ERR190348

ERR190349

ERR190350

ERR190351

ERR190352

ERR190353

ERR190354

ERR190355

ERR190356

ERR190357

ERR190358

ERR190359

ERR190360

ERR190366

ERR190368

ERR190369

ERR190370

ERR190372

ERR190373

ERR190374

ERR190375

ERR190376

ERR190377

ERR190378

ERR190380

ERR190381

ERR190382

ERR190383

ERR190384

ERR190385

ERR190386

ERR190387

ERR190389

ERR190390

ERR190391

ERR190392

ERR190393

ERR190394

ERR190395

ERR190396

ERR190397

ERR190398

ERR190399

ERR190400

ERR190401

ERR190402

ERR190403

ERR190404

ERR190405

ERR190407

ERR190408

ERR190409

ERR190410

ERR211990

ERR211991

ERR211992

ERR211993

ERR211994

ERR211995

ERR211996

ERR211997

ERR211998

ERR211999

ERR212000

ERR212006

ERR212007

ERR212008

ERR212009

ERR212010

ERR212011

ERR212012

ERR212013

ERR212014

ERR212016

ERR212017

ERR212018

ERR212019

ERR212020

ERR212021

ERR212023

ERR212024

ERR212025

ERR212026

ERR212027

ERR212029

ERR212030

ERR212031

ERR212032

ERR212036

ERR212037

ERR212038

ERR212039

ERR212040

ERR212041

ERR212043

ERR212044

ERR212045

ERR212046

ERR212047

ERR212048

ERR212049

ERR212050

ERR212051

ERR212052

ERR212053

ERR212054

ERR212055

ERR212056

ERR212057

ERR212058

ERR212060

ERR212061

ERR212062

ERR212063

ERR212064

ERR212065

ERR212066

ERR212067

ERR212068

ERR212070

ERR212071

ERR212072

ERR212073

ERR212074

ERR212075

ERR212076

ERR212077

ERR212078

ERR212079

ERR212080

ERR212081

ERR212083

ERR212085

ERR212086

ERR212087

ERR212088

ERR212089

ERR212090

ERR212091

ERR212092

ERR212093

ERR212094

ERR212095

ERR212096

ERR212097

ERR212099

ERR212102

ERR212103

ERR212104

ERR212105

ERR212106

ERR212108

ERR212110

ERR212111

ERR212113

ERR212115

ERR212116

ERR212117

ERR212118

ERR212119

ERR212120

ERR212122

ERR212124

ERR212126

ERR212127

ERR212128

ERR212129

ERR212130

ERR212132

ERR212133

ERR212134

ERR212137

ERR212139

ERR212140

ERR212141

ERR212142

ERR212143

ERR212144

ERR212145

ERR212146

ERR212147

ERR212148

ERR212149

ERR212150

ERR212151

ERR212152

ERR212153

ERR212154

ERR212155

ERR212156

ERR212157

ERR212158

ERR212159

ERR212160

ERR212163

ERR212164

ERR212166

ERR212167

ERR212168

ERR212169

ERR212170

ERR212171

ERR212172

ERR212174

ERR212175

ERR212176

ERR212177

ERR212179

ERR212180

ERR212181

ERR216899

ERR216900

ERR216903

ERR216905

ERR216906

ERR216907

ERR216908

ERR216909

ERR216911

ERR216912

ERR216913

ERR216914

ERR216915

ERR216916

ERR216917

ERR216919

ERR216920

ERR216921

ERR216922

ERR216923

ERR216924

ERR216925

ERR216926

ERR216927

ERR216931

ERR216933

ERR216935

ERR216936

ERR216937

ERR216939

ERR216940

ERR216941

ERR216942

ERR216943

ERR216944

ERR216945

ERR216946

ERR216948

ERR216952

ERR216954

ERR216958

ERR216961

ERR216962

ERR216964

ERR216966

ERR216968

ERR216973

ERR216979

ERR216980

ERR216981

ERR216982

ERR216984

ERR216988

ERR216992

ERR221524

ERR221525

ERR221526

ERR221527

ERR221528

ERR221529

ERR221530

ERR221531

ERR221532

ERR221533

ERR221534

ERR221535

ERR221537

ERR221540

ERR221541

ERR221542

ERR221543

ERR221544

ERR221545

ERR221546

ERR221547

ERR221548

ERR221549

ERR221551

ERR221552

ERR221553

ERR221554

ERR221555

ERR221556

ERR221557

ERR221558

ERR221559

ERR221560

ERR221561

ERR221562

ERR221563

ERR221564

ERR221565

ERR221566

ERR221568

ERR221569

ERR221570

ERR221571

ERR221572

ERR221573

ERR221574

ERR221575

ERR221576

ERR221577

ERR221578

ERR221579

ERR221580

ERR221581

ERR221582

ERR221584

ERR221585

ERR221586

ERR221587

ERR221588

ERR221589

ERR221590

ERR221591

ERR221592

ERR221594

ERR221595

ERR221596

ERR221597

ERR221598

ERR221599

ERR221600

ERR221601

ERR221602

ERR221605

ERR221606

ERR221607

ERR221609

ERR221610

ERR221612

ERR221613

ERR221614

ERR221615

ERR221616

ERR221617

ERR221618

ERR221619

ERR227975

ERR227976

ERR227977

ERR227978

ERR227979

ERR227980

ERR227981

ERR227982

ERR227983

ERR227984

ERR227985

ERR227987

ERR227990

ERR227991

ERR227992

ERR227994

ERR227995

ERR227997

ERR227998

ERR227999

ERR228000

ERR228001

ERR228002

ERR228003

ERR228004

ERR228005

ERR228006

ERR228007

ERR228008

ERR228009

ERR228010

ERR228011

ERR228012

ERR228013

ERR228014

ERR228015

ERR228016

ERR228017

ERR228018

ERR228019

ERR228020

ERR228021

ERR228022

ERR228023

ERR228024

ERR228025

ERR228026

ERR228027

ERR228028

ERR228029

ERR228030

ERR228031

ERR228032

ERR228034

ERR228035

ERR228036

ERR228037

ERR228038

ERR228039

ERR228040

ERR228042

ERR228043

ERR228044

ERR228045

ERR228046

ERR228047

ERR228048

ERR228049

ERR228050

ERR228051

ERR228052

ERR228055

ERR228056

ERR228057

ERR228058

ERR228059

ERR228060

ERR228061

ERR228062

ERR228063

ERR228064

ERR228065

ERR228066

ERR228067

ERR228068

ERR228069

ERR229915

ERR229916

ERR229917

ERR229918

ERR229919

ERR229920

ERR229921

ERR229922

ERR229923

ERR229924

ERR229926

ERR229927

ERR229928

ERR229929

ERR229931

ERR229932

ERR229933

ERR229934

ERR229935

ERR229936

ERR229937

ERR229939

ERR229940

ERR229941

ERR229942

ERR229943

ERR229944

ERR229945

ERR229947

ERR229948

ERR229949

ERR229952

ERR229953

ERR229954

ERR229955

ERR229956

ERR229958

ERR229959

ERR229960

ERR229961

ERR229962

ERR229963

ERR229964

ERR229967

ERR229968

ERR229969

ERR229970

ERR229971

ERR229972

ERR229973

ERR229975

ERR229976

ERR229977

ERR229978

ERR229979

ERR229980

ERR229981

ERR229982

ERR229983

ERR229984

ERR229985

ERR229986

ERR229987

ERR229989

ERR229990

ERR229991

ERR229992

ERR229993

ERR229994

ERR229995

ERR229996

ERR229997

ERR229998

ERR229999

ERR230000

ERR230001

ERR230002

ERR230003

ERR230004

ERR230005

ERR230006

ERR230007

ERR230008

ERR230009

ERR230010

ERR234097

ERR234098

ERR234099

ERR234100

ERR234102

ERR234106

ERR234108

ERR234112

ERR234113

ERR234114

ERR234115

ERR234116

ERR234117

ERR234118

ERR234119

ERR234123

ERR234124

ERR234125

ERR234126

ERR234128

ERR234129

ERR234130

ERR234131

ERR234132

ERR234133

ERR234134

ERR234135

ERR234136

ERR234137

ERR234138

ERR234141

ERR234146

ERR234147

ERR234149

ERR234154

ERR234155

ERR234156

ERR234157

ERR234160

ERR234161

ERR234162

ERR234163

ERR234164

ERR234165

ERR234166

ERR234167

ERR234168

ERR234170

ERR234171

ERR234172

ERR234173

ERR234174

ERR234175

ERR234176

ERR234177

ERR234181

ERR234182

ERR234185

ERR234186

ERR234187

ERR234190

ERR234191

ERR234192

ERR234193

ERR234194

ERR234195

ERR234196

ERR234197

ERR234198

ERR234199

ERR234200

ERR234201

ERR234202

ERR234203

ERR234204

ERR234206

ERR234207

ERR234208

ERR234209

ERR234210

ERR234211

ERR234213

ERR234216

ERR234224

ERR234233

ERR234234

ERR234235

ERR234237

ERR234241

ERR234242

ERR234243

ERR234244

ERR234245

ERR234246

ERR234247

ERR234248

ERR234250

ERR234251

ERR234252

ERR234253

ERR234254

ERR234255

ERR234256

ERR234257

ERR234258

ERR234259

ERR234260

ERR234261

ERR234262

ERR234263

ERR234264

ERR234265

ERR234267

ERR234268

ERR234269

ERR234270

ERR234271

ERR234272

ERR234273

ERR234556

ERR234557

ERR234558

ERR234559

ERR234560

ERR234561

ERR234562

ERR234563

ERR234565

ERR234568

ERR234569

ERR234573

ERR234574

ERR234575

ERR234576

ERR234577

ERR234578

ERR234579

ERR234582

ERR234584

ERR234585

ERR234586

ERR234587

ERR234588

ERR234589

ERR234590

ERR234591

ERR234592

ERR234593

ERR234594

ERR234595

ERR234596

ERR234597

ERR234598

ERR234600

ERR234601

ERR234602

ERR234603

ERR234606

ERR234607

ERR234608

ERR234609

ERR234610

ERR234612

ERR234614

ERR234615

ERR234616

ERR234617

ERR234618

ERR234619

ERR234620

ERR234621

ERR234622

ERR234623

ERR234624

ERR234627

ERR234628

ERR234629

ERR234630

ERR234632

ERR234633

ERR234634

ERR234636

ERR234637

ERR234638

ERR234640

ERR234641

ERR234642

ERR234643

ERR234644

ERR234645

ERR234646

ERR234647

ERR234648

ERR234649

ERR234650

ERR234651

ERR234652

ERR234653

ERR234654

ERR234655

ERR234656

ERR234657

ERR234658

ERR234660

ERR234661

ERR234662

ERR234663

ERR234664

ERR234665

ERR234668

ERR234671

ERR234672

ERR234674

ERR234683

ERR234684

ERR234687

ERR234688

ERR234689

ERR234690

ERR234691

ERR234692

ERR234693

ERR234694

ERR234696

ERR234697

ERR234698

ERR234699

ERR245646

ERR245647

ERR245648

ERR245649

ERR245650

ERR245651

ERR245652

ERR245653

ERR245654

ERR245655

ERR245656

ERR245657

ERR245658

ERR245659

ERR245660

ERR245661

ERR245663

ERR245664

ERR245665

ERR245666

ERR245667

ERR245668

ERR245669

ERR245671

ERR245672

ERR245673

ERR245674

ERR245676

ERR245677

ERR245678

ERR245679

ERR245680

ERR245681

ERR245682

ERR245683

ERR245684

ERR245685

ERR245686

ERR245687

ERR245688

ERR245689

ERR245690

ERR245692

ERR245693

ERR245694

ERR245695

ERR245696

ERR245697

ERR245698

ERR245699

ERR245700

ERR245701

ERR245702

ERR245703

ERR245704

ERR245705

ERR245706

ERR245707

ERR245708

ERR245709

ERR245710

ERR245711

ERR245713

ERR245714

ERR245715

ERR245717

ERR245718

ERR245719

ERR245720

ERR245721

ERR245722

ERR245723

ERR245724

ERR245725

ERR245726

ERR245727

ERR245728

ERR245729

ERR245730

ERR245731

ERR245732

ERR245733

ERR245735

ERR245736

ERR245737

ERR245738

ERR245739

ERR245741

ERR245742

ERR245743

ERR245744

ERR245745

ERR245746

ERR245748

ERR245749

ERR245750

ERR245751

ERR245752

ERR245753

ERR245755

ERR245756

ERR245757

ERR245759

ERR245760

ERR245761

ERR245763

ERR245765

ERR245767

ERR245768

ERR245770

ERR245773

ERR245774

ERR245776

ERR245777

ERR245778

ERR245779

ERR245781

ERR245782

ERR245783

ERR245784

ERR245786

ERR245787

ERR245788

ERR245789

ERR245791

ERR245792

ERR245796

ERR245797

ERR245798

ERR245799

ERR245800

ERR245802

ERR245803

ERR245804

ERR245805

ERR245806

ERR245807

ERR245808

ERR245811

ERR245812

ERR245813

ERR245815

ERR245817

ERR245818

ERR245819

ERR245820

ERR245821

ERR245822

ERR245823

ERR245825

ERR245826

ERR245828

ERR245829

ERR245830

ERR245831

ERR245832

ERR245833

ERR245834

ERR245835

ERR245836

ERR245837

ERR245838

ERR245839

ERR245840

ERR245841

ERR245842

ERR245843

ERR245844

ERR245845

ERR245847

ERR245848

ERR245849

ERR257891

ERR257892

ERR257893

ERR257894

ERR257895

ERR257896

ERR257897

ERR257898

ERR257899

ERR257900

ERR257901

ERR257902

ERR257903

ERR257904

ERR257905

ERR257906

ERR257907

ERR257908

ERR257909

ERR257910

ERR257911

ERR257912

ERR257913

ERR257914

ERR257915

ERR257916

ERR257917

ERR257918

ERR257919

ERR257920

ERR257922

ERR257923

ERR257924

ERR257925

ERR257926

ERR257927

ERR257928

ERR257929

ERR257930

ERR257931

ERR257932

ERR257933

ERR257934

ERR257935

ERR275181

ERR275182

ERR275183

ERR275184

ERR275190

ERR275191

ERR275192

ERR275193

ERR275194

ERR275195

ERR275196

ERR275198

ERR275199

ERR275200

ERR275201

ERR275202

ERR275203

ERR275204

ERR275205

ERR275206

ERR275207

ERR275208

ERR275210

ERR275211

ERR275212

ERR275213

ERR275214

ERR275215

ERR275216

ERR275217

ERR275219

ERR275220

ERR275222

ERR275223

ERR275224

ERR275225

ERR275226

ERR275227

ERR275229

ERR275230

ERR275231

ERR369586

ERR369587

ERR369588

ERR369589

ERR369590

ERR369591

ERR369592

ERR369593

ERR369594

ERR369595

ERR369596

ERR369597

ERR369598

ERR369599

ERR369600

ERR369601

ERR369602

ERR369603

ERR369604

ERR369605

ERR369606

ERR369607

ERR369608

ERR369609

ERR369610

ERR369611

ERR369612

ERR369613

ERR369614

ERR369615

ERR369616

ERR369617

ERR369618

ERR369619

ERR369620

ERR369621

ERR369622

ERR369623

ERR369624

ERR369625

ERR369626

ERR369627

ERR369628

ERR369629

ERR369630

ERR369631

ERR369632

ERR369633

ERR369634

ERR369635

ERR369636

ERR369637

ERR369638

ERR369639

ERR369640

ERR369641

ERR369642

ERR369643

ERR369644

ERR369645

ERR369646

ERR369647

ERR369648

ERR369649

ERR369650

ERR369651

ERR369652

ERR369653

ERR369654

ERR369655

ERR369656

ERR369657

ERR369658

ERR369659

ERR369660

ERR369661

ERR369662

ERR369663

ERR369664

ERR369665

ERR369666

ERR369667

ERR369668

ERR369669

ERR369670

ERR369671

ERR369672

ERR369673

ERR369674

ERR369675

ERR369676

ERR369677

ERR369678

ERR369679

ERR369680

ERR369681

ERR369682

ERR369683

ERR369684

ERR369685

ERR369686

ERR369687

ERR369688

ERR369689

ERR369690

ERR369691

ERR369692

ERR369693

ERR369694

ERR369695

ERR369696

ERR369697

ERR369698

ERR369699

ERR369700

ERR369701

ERR369702

ERR369703

ERR369704

ERR369705

ERR369706

ERR369707

ERR369708

ERR369709

ERR369710

ERR369711

ERR369712

ERR369713

ERR369714

ERR369715

ERR369716

ERR369717

ERR369718

ERR369719

ERR369720

ERR369721

ERR369722

ERR369723

ERR369724

ERR369725

ERR369726

ERR369727

ERR369728

ERR369729

ERR369730

ERR369731

ERR369732

ERR369733

ERR369734

ERR369735

ERR369736

ERR369737

ERR369738

ERR369739

ERR369740

ERR369741

ERR369742

ERR369743

ERR369744

ERR369745

ERR369746

ERR369747

ERR369748

ERR369749

ERR369750

ERR369751

ERR369752

ERR369753

ERR369754

ERR369755

ERR369756

ERR369757

ERR369758

ERR369759

ERR400307

ERR400308

ERR400309

ERR400311

ERR400312

ERR400313

ERR400314

ERR400316

ERR400317

ERR400318

ERR400319

ERR400324

ERR400325

ERR400327

ERR400328

ERR400329

ERR400330

ERR400331

ERR400332

ERR400333

ERR400334

ERR400337

ERR400338

ERR400339

ERR400340

ERR400341

ERR400342

ERR400343

ERR400347

ERR400348

ERR400351

ERR400352

ERR400353

ERR400354

ERR400357

ERR400358

ERR400364

ERR400368

ERR400369

ERR400370

ERR400375

ERR400377

ERR400378

ERR400379

ERR400380

ERR400382

ERR400383

ERR400386

ERR400387

ERR400389

ERR400390

ERR400391

ERR400392

ERR400393

ERR400394

ERR400395

ERR400396

ERR400397

ERR400398

ERR400400

ERR400402

ERR400403

ERR400404

ERR400405

ERR400407

ERR400408

ERR400409

ERR400413

ERR400415

ERR400416

ERR400417

ERR400419

ERR400421

ERR400422

ERR400423

ERR400424

ERR400425

ERR400426

ERR400427

ERR400429

ERR400430

ERR400431

ERR400432

ERR400433

ERR400434

ERR400435

ERR400436

ERR400437

ERR400439

ERR400440

ERR400441

ERR400442

ERR400444

ERR400446

ERR400447

ERR400449

ERR400450

ERR400451

ERR400452

ERR400453

ERR400455

ERR400457

ERR400458

ERR400459

ERR400462

ERR400463

ERR400464

ERR400466

ERR400467

ERR400468

ERR400469

ERR400470

ERR400471

ERR400472

ERR400473

ERR400474

ERR400475

ERR400476

ERR400478

ERR400479

ERR400481

ERR400483

ERR400487

ERR400489

ERR400491

ERR400492

ERR400493

ERR400494

ERR400497

ERR400498

ERR400500

ERR400504

ERR400505

ERR400506

ERR400507

ERR400509

ERR400510

ERR400511

ERR400512

ERR400513

ERR400514

ERR400515

ERR400516

ERR400518

ERR400519

ERR400520

ERR400522

ERR400523

ERR400524

ERR400525

ERR400526

ERR400527

ERR400528

ERR400530

ERR400531

ERR400532

ERR400533

ERR400534

ERR400536

ERR400537

ERR400540

ERR400541

ERR400542

ERR400543

ERR400545

ERR400546

ERR400547

ERR400549

ERR400551

ERR400552

ERR400553

ERR400555

ERR400556

ERR400557

ERR502240

ERR502241

ERR502242

ERR502243

ERR502244

ERR502245

ERR502246

ERR502247

ERR502248

ERR502249

ERR502250

ERR502251

ERR502252

ERR502253

ERR502254

ERR502255

ERR502256

ERR502257

ERR502258

ERR502259

ERR502260

ERR502261

ERR502262

ERR502263

ERR502264

ERR502265

ERR502266

ERR502267

ERR502268

ERR502269

ERR502270

ERR502271

ERR502272

ERR502273

ERR502274

ERR502275

ERR502276

ERR502277

ERR502278

ERR502279

ERR502280

ERR502281

ERR502282

ERR502283

ERR502284

ERR502285

ERR502286

ERR502287

ERR502288

ERR502289

ERR502290

ERR502291

ERR502292

ERR502293

ERR502294

ERR502295

ERR502296

ERR502297

ERR502298

ERR502299

ERR502300

ERR502301

ERR502302

ERR502303

ERR502304

ERR502305

ERR502306

ERR502307

ERR502308

ERR502309

ERR502310

ERR502311

ERR502312

ERR502313

ERR502314

ERR502315

ERR502316

ERR502317

ERR502318

ERR502319

ERR502321

ERR502322

ERR502323

ERR502324

ERR502325

ERR502326

ERR502327

ERR502329

ERR502330

ERR502331

ERR502332

ERR502333

ERR502334

ERR502335

ERR502336

ERR502337

ERR502338

ERR502339

ERR502340

ERR502341

ERR502342

ERR502343

ERR502344

ERR502345

ERR502346

ERR502347

ERR502348

ERR502349

ERR502350

ERR502351

ERR502352

ERR502353

ERR502354

ERR502355

ERR502356

ERR502357

ERR502358

ERR502359

ERR502360

ERR502361

ERR502362

ERR502363

ERR502364

ERR502365

ERR502366

ERR502367

ERR502368

ERR502369

ERR502370

ERR502371

ERR502372

ERR502373

ERR502374

ERR502375

ERR502376

ERR502377

ERR502378

ERR502379

ERR502380

ERR502381

ERR502382

ERR502383

ERR502384

ERR502385

ERR502386

ERR502387

ERR502388

ERR502389

ERR502390

ERR502391

ERR502392

ERR502393

ERR502394

ERR502395

ERR502396

ERR502397

ERR502398

ERR502399

ERR502400

ERR502401

ERR502402

ERR502403

ERR502404

ERR502405

ERR502406

ERR502407

ERR502408

ERR502409

ERR502410

ERR502411

ERR502412

ERR502413

ERR502414

ERR502415

ERR502416

ERR502417

ERR502418

ERR502419

ERR502420

ERR502421

ERR502422

ERR502423

ERR502424

ERR502425

ERR502426

ERR502427

ERR502428

ERR502429

ERR517399

ERR517400

ERR550644

ERR550659

ERR550665

ERR550670

ERR550712

ERR550724

ERR550729

ERR550738

ERR550739

ERR550778

ERR550782

ERR550887

ERR550901

ERR550910

ERR550927

ERR550940

ERR550942

ERR550946

ERR550957

ERR550984

ERR551007

ERR551038

ERR551067

ERR551071

ERR551079

ERR551086

ERR551089

ERR551090

ERR551101

ERR551155

ERR551159

ERR551168

ERR551184

ERR551192

ERR551197

ERR551201

ERR551212

ERR551225

ERR551254

ERR551293

ERR551305

ERR551311

ERR551336

ERR551360

ERR551370

ERR551398

ERR551412

ERR551419

ERR551438

ERR551494

ERR551549

ERR551550

ERR551554

ERR551556

ERR551566

ERR551568

ERR551572

ERR551620

ERR551636

ERR551638

ERR551680

ERR551688

ERR551694

ERR551725

ERR551772

ERR551804

ERR551822

ERR551847

ERR551854

ERR551857

ERR551879

ERR551915

ERR551928

ERR551930

ERR551934

ERR551945

ERR551956

ERR551978

ERR551990

ERR551998

ERR552081

ERR552090

ERR552093

ERR552095

ERR552116

ERR552130

ERR552132

ERR552136

ERR552141

ERR552159

ERR552177

ERR552190

ERR552194

ERR552219

ERR552246

ERR552259

ERR552267

ERR552281

ERR552331

ERR552358

ERR552411

ERR552427

ERR552429

ERR552444

ERR552479

ERR552482

ERR552493

ERR552549

ERR552553

ERR552555

ERR552580

ERR552647

ERR552662

ERR552668

ERR552689

ERR552707

ERR552728

ERR552743

ERR552755

ERR552760

ERR552787

ERR552799

ERR552830

ERR552836

ERR552838

ERR552879

ERR552894

ERR552907

ERR552910

ERR552912

ERR552935

ERR552939

ERR552949

ERR552954

ERR552979

ERR553009

ERR553030

ERR553037

ERR553068

ERR553082

ERR553086

ERR553098

ERR553107

ERR553116

ERR553130

ERR553139

ERR553156

ERR553171

ERR553211

ERR553226

ERR553237

ERR553251

ERR553258

ERR553274

ERR553277

ERR553286

ERR553291

ERR553304

ERR553313

ERR553314

ERR553324

ERR553347

ERR553371

ERR553386

ERR568778

ERR568779

ERR600630

ERR600631

ERR600632

ERR600633

ERR600634

ERR600635

ERR600636

ERR600637

ERR600638

ERR600639

ERR600640

ERR600641

ERR600642

ERR600643

ERR600644

ERR600645

ERR600646

ERR600647

ERR600648

ERR600649

ERR600650

ERR600651

ERR600652

ERR600653

ERR600654

ERR600655

ERR600656

ERR600657

ERR600658

ERR600659

ERR600660

ERR600661

ERR600662

ERR600663

ERR600664

ERR600665

ERR600666

ERR600667

ERR600668

ERR600669

ERR600670

ERR600671

ERR600672

ERR600673

ERR600674

ERR600675

ERR600676

ERR619080

IDR1100019254

IDR1100020842

IDR1100023189

IDR1200001956

IDR1200002066

IDR1200003374

IDR1200008928

IDR1200010128

IDR1200022433

IDR1200022434

IDR1200023774

IDR1200028162

IDR1200030168

IDR1300005060

IDR1300010061

IDR1300015499

IDR1300016371

IDR1300017766

IDR1300018388

IDR1300019614

IDR1300021110

IDR1300022959

IDR1300023467

IDR1300033719

IDR1300038288

IDR1400001909

IDR1400002530

IDR1400002533

IDR1400003506

IDR1400004223

IDR1400006549

IDR1400007566

IDR1400008041

IDR1400009529

IDR1400010021

IDR1400011176

IDR1400014851

IDR1400016559

IDR1400026616

IDR1400026773

IDR1400028307

IDR1400029413

IDR1400029458

IDR1400032120

IDR1400032281

IDR1400032735

IDR1400033739

IDR1400033866

IDR1400034450

IDR1400036339

IDR1400036823

IDR1500000665

IDR1500003469

IDR1500005031

IDR1500006079

IDR1500006146

IDR1500007097

IDR1500007955

IDR1500008439

IDR1500009074

IDR1500009344

IDR1500009607

IDR1500010219

IDR1500011607

IDR1500045314

IDR1500045852

IDR1500046445

IDR1500047122

IDR1500048671

IDR1500048830

IDR1500048831

IDR1500049548

IDR1500049762

IDR1500049950

IDR1500050042

IDR1500050467

IDR1500051087

IDR1500051244

IDR1500051245

IDR1500051247

IDR1500051248

IDR1500051477

IDR1500051827

IDR1500051828

IDR1500052024

IDR1500052246

IDR1500052248

IDR1500052538

IDR1500052791

IDR1500052794

IDR1500052969

IDR1500053361

IDR1500053362

IDR1500053652

IDR1500053660

IDR1500054144

IDR1500054145

IDR1500054147

IDR1500054148

IDR1500054445

IDR1500054922

IDR1500055140

IDR1500055141

IDR1500055344

IDR1500055357

IDR1500055398

IDR1500055782

IDR1500056763

IDR1500056764

IDR1500056766

IDR1500056767

IDR1500057022

IDR1500057177

IDR1500057631

IDR1500057969

IDR1500057970

IDR1500058176

IDR1500058276

IDR1500058475

IDR1500059057

IDR1500059313

IDR1500059320

IDR1500059803

IDR1500059889

IDR1500060570

IDR1500061013

IDR1500061127

IDR1500061133

IDR1500061876

IDR1500061877

IDR1500061908

IDR1500061909

IDR1500061962

IDR1500062006

IDR1500062021

IDR1500062042

IDR1500062168

IDR1500062169

IDR1500062183

IDR1500062349

IDR1500062350

IDR1500062509

IDR1500062511

IDR1500062512

IDR1500062635

IDR1500062818

IDR1500062826

IDR1500063002

IDR1500063075

IDR1500063077

IDR1500063136

IDR1500063304

IDR1500063732

IDR1500064035

IDR1500064037

IDR1500064041

IDR1500064042

IDR1500065550

IDR1500065667

IDR1500065671

IDR1500065673

IDR1500065702

IDR1500065704

IDR1500066192

IDR1500066354

IDR1500066660

IDR1500066661

IDR1500066750

IDR1500066944

IDR1500066946

IDR1500067319

IDR1500067320

IDR1500067323

IDR1500067462

IDR1500067832

IDR1500067837

IDR1500067984

IDR1500068120

IDR1500068393

IDR1500068626

IDR1500068721

IDR1500068895

IDR1500068896

IDR1500069348

IDR1500069613

IDR1500069614

IDR1500069726

IDR1500069795

IDR1500069797

IDR1500070055

IDR1500070283

IDR1500070812

IDR1500070900

IDR1500070963

IDR1500070964

IDR1500071153

IDR1500071331

IDR1500071800

IDR1500071801

IDR1500071802

IDR1500072113

IDR1500072152

IDR1500072153

IDR1500072154

IDR1500072159

IDR1500072206

IDR1500072381

IDR1500072503

IDR1500072620

IDR1500072634

IDR1500072635

IDR1500072638

IDR1500072639

IDR1500072644

IDR1500072647

IDR1500072657

IDR1500072659

IDR1500072738

IDR1600000081

IDR1600000150

IDR1600000160

IDR1600000287

IDR1600000436

IDR1600000474

IDR1600000481

IDR1600000553

IDR1600000568

IDR1600000642

IDR1600000643

IDR1600000726

IDR1600000941

IDR1600001030

IDR1600001032

IDR1600001033

IDR1600001037

IDR1600001040

IDR1600001077

IDR1600001203

IDR1600001648

IDR1600001649

IDR1600001715

IDR1600001721

IDR1600001727

IDR1600001742

IDR1600001775

IDR1600001810

IDR1600001950

IDR1600001953

IDR1600002109

IDR1600002304

IDR1600002325

IDR1600002326

IDR1600002328

IDR1600003209

IDR1600003433

IDR1600003443

IDR1600003444

IDR1600003471

IDR1600003472

IDR1600003488

IDR1600003494

IDR1600003497

IDR1600003526

IDR1600003528

IDR1600003586

IDR1600003600

IDR1600003605

IDR1600003631

IDR1600003632

IDR1600003652

IDR1600003653

IDR1600003684

IDR1600003708

IDR1600003836

IDR1600003837

IDR1600004178

IDR1600004382

IDR1600004865

IDR1600004867

IDR1600005536

IDR1600005538

IDR1600005546

IDR1600005548

IDR1600005829

IDR1600006600

IDR1600006601

IDR1600006602

IDR1600006603

IDR1600006604

IDR1600006605

IDR1600006606

IDR1600006607

IDR1600006608

IDR1600006610

IDR1600006613

IDR1600006615

IDR1600006616

IDR1600006792

IDR1600007779

IDR1600007932

IDR1600007935

IDR1600007940

IDR1600007943

IDR1600007946

IDR1600007948

IDR1600007963

IDR1600007968

IDR1600007978

IDR1600007982

IDR1600008116

IDR1600008133

IDR1600008140

IDR1600008151

IDR1600008238

IDR1600009864

IDR1600009867

IDR1600010492

IDR1600010494

IDR1600010936

IDR1600010967

IDR1600010970

IDR1600011016

IDR1600011024

IDR1600011059

IDR1600011061

IDR1600011067

IDR1600011093

IDR1600011115

IDR1600011117

IDR1600011118

IDR1600011125

IDR1600011126

IDR1600011129

IDR1600011183

IDR1600011187

IDR1600011192

IDR1600011196

IDR1600011801

IDR1600012094

IDR1600012207

IDR1600012456

IDR1600012613

IDR1600012617

IDR1600012620

IDR1600012632

IDR1600012638

IDR1600012674

IDR1600012753

IDR1600012758

IDR1600012759

IDR1600012974

IDR1600014121

IDR1600014124

IDR1600014199

IDR1600014203

IDR1600014212

IDR1600014409

IDR1600015259

IDR1600015297

IDR1600015299

IDR1600015300

IDR1600015301

IDR1600015305

IDR1600015313

IDR1600015314

IDR1600015315

IDR1600015324

IDR1600015327

IDR1600015344

IDR1600015741

IDR1600016611

IDR1600016903

IDR1600016926

IDR1600016927

IDR1600016932

IDR1600016942

IDR1600016945

IDR1600016962

IDR1600016963

IDR1600016964

IDR1600017091

IDR1600017097

IDR1600017137

IDR1600017466

IDR1600017477

IDR1600017749

IDR1600017789

IDR1600017791

IDR1600017792

IDR1600017793

IDR1600017794

IDR1600017828

IDR1600017905

IDR1600017938

IDR1600018162

IDR1600018167

IDR1600018194

IDR1600018197

IDR1600018201

IDR1600018716

IDR1600018864

IDR1600018867

IDR1600018872

IDR1600018875

IDR1600018877

IDR1600018878

IDR1600018879

IDR1600018880

IDR1600018886

IDR1600019639

IDR1600019640

IDR1600019963

IDR1600019964

IDR1600019965

IDR1600019966

IDR1600019997

IDR1600020057

IDR1600020177

IDR1600020192

IDR1600020523

IDR1600020570

IDR1600020737

IDR1600020953

IDR1600020957

IDR1600021019

IDR1600021022

IDR1600021025

IDR1600021029

IDR1600021054

IDR1600021226

IDR1600021315

IDR1600021320

IDR1600021878

IDR1600022106

IDR1600022144

IDR1600022146

IDR1600022147

IDR1600022148

IDR1600022173

IDR1600022177

IDR1600022368

IDR1600022505

IDR1600022826

IDR1600022827

IDR1600022912

IDR1600023068

IDR1600023111

IDR1600023170

IDR1600023172

IDR1600023178

IDR1600023254

IDR1600023255

IDR1600023256

IDR1600023647

IDR1600023714

IDR1600023715

IDR1600023875

IDR1600023909

IDR1600023912

IDR1600023914

IDR1600023915

IDR1600023916

IDR1600023920

IDR1600023926

IDR1600024367

IDR1600024547

IDR1600025151

IDR1600025154

IDR1600025156

IDR1600025158

IDR1600025163

IDR1600025175

IDR1600025205

IDR1600025236

IDR1600025257

IDR1600025428

IDR1600025432

IDR1600025436

IDR1600025442

IDR1600026227

IDR1600026228

IDR1600026421

IDR1600026427

IDR1600026431

IDR1600026432

IDR1600026433

IDR1600026434

IDR1600026436

IDR1600026439

IDR1600026542

IDR1600026642

IDR1600026643

IDR1600026645

IDR1600026772

IDR1600027139

IDR1600027315

IDR1600027328

IDR1600027850

IDR1600027851

IDR1600027856

IDR1600027858

IDR1600027861

IDR1600027867

IDR1600027869

IDR1600027871

IDR1600027872

IDR1600027877

IDR1600027999

IDR1600028000

IDR1600028279

IDR1600028520

IDR1600028710

IDR1600028711

IDR1600028712

IDR1600029323

IDR1600029328

IDR1600029330

IDR1600029337

IDR1600029363

IDR1600029367

IDR1600029372

IDR1600029540

IDR1600029939

IDR1600030328

IDR1600030697

IDR1600030698

IDR1600030701

IDR1600030702

IDR1600030703

IDR1600030704

IDR1600030705

IDR1600030706

IDR1600030707

IDR1600030712

IDR1600030717

IDR1600030763

IDR1600031131

IDR1600031132

IDR1600031133

IDR1600031212

IDR1600031214

IDR1600031216

IDR1600031218

IDR1600031219

IDR1600031220

IDR1600031221

IDR1600031222

IDR1600031223

IDR1600031224

IDR1600031225

IDR1600031226

IDR1600031228

IDR1600031229

IDR1600031230

IDR1600031529

IDR1600031534

IDR1600031875

IDR1600032077

IDR1600032078

IDR1600032237

IDR1600032238

IDR1600032241

IDR1600032242

IDR1600032245

IDR1600032246

IDR1600032248

IDR1600032334

IDR1600032721

IDR1600033122

IDR1600033123

IDR1600033125

IDR1600033669

IDR1600033672

IDR1600033673

IDR1600033675

IDR1600033680

IDR1600033688

IDR1600033693

IDR1600034035

IDR1600034314

IDR1600034868

IDR1600034869

IDR1600034870

IDR1600035065

IDR1600035086

IDR1600035094

IDR1600035100

IDR1600035104

IDR1600035108

IDR1600035109

IDR1600035110

IDR1600035114

IDR1600035117

IDR1600035122

IDR1600035148

IDR1600035227

IDR1600035377

IDR1600035708

IDR1600035709

IDR1600035710

IDR1600035712

IDR1600035713

IDR1600035714

IDR1600035715

IDR1600035716

IDR1600035835

IDR1600035837

IDR1600036511

IDR1600036513

IDR1600037085

IDR1600037087

IDR1600037090

IDR1600037093

IDR1600037096

IDR1600037099

IDR1600037103

IDR1600037187

IDR1600037413

IDR1600037626

IDR1600037628

IDR1600037632

IDR1600037777

IDR1600037778

IDR1600037795

IDR1600037800

IDR1600037805

IDR1600037811

IDR1600037815

IDR1600038976

IDR1600039182

IDR1600039184

IDR1600039759

IDR1600039942

IDR1600039943

IDR1600039944

IDR1600039948

IDR1600039950

IDR1600039951

IDR1600039954

IDR1600039956

IDR1600039957

IDR1600039958

IDR1600039961

IDR1600039993

IDR1600040178

IDR1600040333

IDR1600040334

IDR1600040335

IDR1600040948

IDR1600040949

IDR1600040950

IDR1600040951

IDR1600040955

IDR1600040957

IDR1600040958

IDR1600040959

IDR1600040964

IDR1600040965

IDR1600040970

IDR1600041271

IDR1600041577

IDR1600041578

IDR1600041579

IDR1600041936

IDR1600041971

IDR1600041972

IDR1600041974

IDR1600041977

IDR1600041984

IDR1600041989

IDR1600041992

IDR1600041993

IDR1600041995

IDR1600041996

IDR1600041998

IDR1600042103

IDR1600042111

IDR1600042114

IDR1600042261

IDR1600042927

IDR1600042929

IDR1600042931

IDR1600044034

IDR1600044037

IDR1600044038

IDR1600044247

IDR1600044249

IDR1600044251

IDR1600044255

IDR1600044256

IDR1600044258

IDR1600044261

IDR1600044262

IDR1600044263

IDR1600044280

IDR1600044301

IDR1600044954

IDR1600044955

IDR1600045100

IDR1600045106

IDR1600045481

IDR1600045994

IDR1600046601

IDR1600046602

IDR1600046603

IDR1600046605

IDR1600046606

IDR1600046607

IDR1600046608

IDR1600046610

IDR1600046611

IDR1600046612

IDR1600046615

IDR1600046616

IDR1600046643

IDR1600046655

IDR1600047135

IDR1600047580

IDR1600047582

IDR1600047583

IDR1600047584

IDR1600047585

IDR1600047588

IDR1600047589

IDR1600047649

IDR1600048331

IDR1600048388

IDR1600048587

IDR1600048588

IDR1600048591

IDR1600048594

IDR1600048595

IDR1600048617

IDR1600048627

IDR1600048632

IDR1600048719

IDR1600048722

IDR1600049248

IDR1600049307

IDR1600049470

IDR1600049474

IDR1600049475

IDR1600049476

IDR1600049478

IDR1600049479

IDR1600049480

IDR1600049481

IDR1600049652

IDR1600049654

IDR1600049674

IDR1600049832

IDR1600050504

IDR1600050505

IDR1600050506

IDR1600050508

IDR1600050509

IDR1600050510

IDR1600050511

IDR1600050513

IDR1600050515

IDR1600050516

IDR1600050517

IDR1600051430

IDR1600051431

IDR1600051546

IDR1600051692

IDR1600051693

IDR1600051695

IDR1600051697

IDR1600051698

IDR1600052366

IDR1600052648

IDR1600052649

IDR1600052650

IDR1600052651

IDR1600052673

IDR1600052869

IDR1600053030

IDR1600053034

IDR1600053042

IDR1600053044

IDR1600053046

IDR1600053047

IDR1600053395

IDR1600053398

IDR1600053401

IDR1600053402

IDR1600053403

IDR1600053405

IDR1600053407

IDR1600053410

IDR1600053412

IDR1600053414

IDR1600053446

IDR1600053447

IDR1600053449

IDR1600054125

IDR1600054127

IDR1600054129

IDR1600054131

IDR1600054132

IDR1600054133

IDR1600054134

IDR1600054135

IDR1600054136

IDR1600054265

IDR1600055023

IDR1600055074

IDR1600055075

IDR1600055551

IDR1600055552

IDR1600055553

IDR1600055554

IDR1600055555

IDR1600055556

IDR1600055557

IDR1600055748

IDR1600055996

IDR1600055997

IDR1600056424

IDR1600056520

IDR1600056521

IDR1600056522

IDR1600056523

IDR1600056524

IDR1600056525

IDR1600056526

IDR1600057520

IDR1600057546

IDR1600057607

IDR1600057619

IDR1600057627

IDR1600057629

IDR1600057632

IDR1600057633

IDR1600057635

IDR1600057637

IDR1600057642

IDR1600057644

IDR1600057645

IDR1600057646

IDR1600057647

IDR1600057650

IDR1600058060

IDR1600058226

IDR1600058228

IDR1600058229

IDR1600058231

IDR1600058563

IDR1600058695

IDR1600058701

IDR1700000430

IDR1700000549

IDR1700000551

IDR1700000552

IDR1700000553

IDR1700000557

IDR1700000559

IDR1700000565

IDR1700000568

IDR1700000569

IDR1700000570

IDR1700000571

IDR1700000573

IDR1700000668

IDR1700000670

IDR1700001063

IDR1700001064

IDR1700001066

IDR1700001067

IDR1700001341

IDR1700001550

IDR1700001735

IDR1700001745

IDR1700002099

IDR1700002448

IDR1700002453

IDR1700002455

IDR1700002459

IDR1700002460

IDR1700002461

IDR1700002464

IDR1700002467

IDR1700002468

IDR1700002469

IDR1700002470

IDR1700002714

IDR1700003363

IDR1700003615

IDR1700003626

IDR1700003628

IDR1700003632

IDR1700003645

IDR1700003662

IDR1700004200

IDR1700005356

IDR1700005438

IDR1700005439

IDR1700005440

IDR1700005441

IDR1700005442

IDR1700005443

IDR1700005444

IDR1700005446

IDR1700005447

IDR1700005448

IDR1700005449

IDR1700005453

IDR1700006498

IDR1700006499

IDR1700007537

IDR1700007538

IDR1700007540

IDR1700007541

IDR1700007543

IDR1700007545

IDR1700007547

IDR1700007548

IDR1700007549

IDR1700007551

IDR1700007552

IDR1700008760

IDR1700008762

IDR1700008763

IDR1700008765

IDR1700008768

IDR1700008773

IDR1700008792

IDR1700008793

IDR1700008794

IDR1700008839

IDR1700008840

NLA000000560

NLA000000709

NLA000001061

NLA000001768

NLA000001969

NLA000016726

NLA000016764

NLA000016985

NLA000017603

NLA000017744

NLA000017848

NLA000017891

NLA000018190

NLA000100143

NLA000100450

NLA000100510

NLA000102124

NLA000200433

NLA000200442

NLA000201244

NLA000202008

NLA000300865

NLA000301029

NLA000301067

NLA000301074

NLA000301077

NLA000301128

NLA000301359

NLA000301652

NLA000301715

NLA000401210

NLA000401230

NLA000401745

NLA000701201

NLA000801694

NLA000801696

NLA009400322

NLA009402429

NLA009500011

NLA009500730

NLA009501246

NLA009501310

NLA009600108

NLA009600800

NLA009600875

NLA009601360

NLA009601659

NLA009601810

NLA009700296

NLA009700983

NLA009701444

NLA009701714

NLA009701938

NLA009702211

NLA009800926

NLA009801164

NLA009801500

NLA009801661

NLA009900180

NLA009900182

NLA009900478

NLA009900895

NLA009901267

NLA009901304

NLA009901354

Peru2901

Peru2902

Peru2903

Peru2904

Peru2905

Peru2906

Peru2907

Peru2908

Peru2909

Peru2910

Peru2911

Peru2912

Peru2913

Peru2914

Peru2915

Peru2916

Peru2917

Peru2918

Peru2919

Peru2920

Peru2921

Peru2922

Peru2923

Peru2924

Peru2925

Peru2926

Peru2927

Peru2928

Peru2931

Peru2932

Peru2933

Peru2935

Peru2937

Peru2938

Peru2939

Peru2940

Peru2941

Peru2942

Peru2943

Peru2944

Peru2945

Peru2946

Peru2947

Peru2948

Peru2949

Peru2950

Peru2951

Peru2952

Peru2953

Peru2954

Peru2955

Peru2956

Peru2957

Peru2959

Peru2960

Peru2961

Peru2962

Peru2963

Peru2964

Peru2965

Peru2966

Peru2967

Peru2968

Peru2969

Peru2970

Peru2971

Peru2972

Peru2973

Peru2974

Peru2976

Peru2977

Peru2978

Peru2979

Peru2980

Peru2981

Peru3001

Peru3002

Peru3003

Peru3004

Peru3005

Peru3006

Peru3007

Peru3008

Peru3009

Peru3010

Peru3011

Peru3012

Peru3013

Peru3014

Peru3015

Peru3016

Peru3017

Peru3018

Peru3019

Peru3020

Peru3021

Peru3022

Peru3023

Peru3024

Peru3025

Peru3026

Peru3027

Peru3029

Peru3030

Peru3031

Peru3032

Peru3033

Peru3037

Peru3038

Peru3039

Peru3040

Peru3041

Peru3042

Peru3043

Peru3044

Peru3045

Peru3046

Peru3047

Peru3048

Peru3049

Peru3052

Peru3054

Peru3055

Peru3056

Peru3057

Peru3058

Peru3059

Peru3060

Peru3061

Peru3062

Peru3063

Peru3064

Peru3065

Peru3066

Peru3067

Peru3068

Peru3069

Peru3070

Peru3253

Peru3255

Peru3257

Peru3258

Peru3259

Peru3260

Peru3261

Peru3262

Peru3263

Peru3264

Peru3265

Peru3266

Peru3267

Peru3268

Peru3269

Peru3271

Peru3272

Peru3274

Peru3275

Peru3276

Peru3277

Peru3278

Peru3279

Peru3280

Peru3281

Peru3283

Peru3284

Peru3285

Peru3286

Peru3287

Peru3288

Peru3289

Peru3290

Peru3291

Peru3292

Peru3293

Peru3294

Peru3295

Peru3296

Peru3297

Peru3298

Peru3299

Peru3301

Peru3302

Peru3303

Peru3304

Peru3305

Peru3306

Peru3307

Peru3308

Peru3309

Peru3310

Peru3311

Peru3312

Peru3313

Peru3314

Peru3315

Peru3316

Peru3317

Peru3318

Peru3319

Peru3320

Peru3321

Peru3322

Peru3323

Peru3324

Peru3326

Peru3327

Peru3329

Peru3330

Peru3331

Peru3332

Peru3333

Peru3334

Peru3335

Peru3338

Peru3339

Peru3340

Peru3342

Peru3344

Peru3345

Peru3347

Peru3348

Peru3349

Peru3350

Peru3351

Peru3352

Peru3354

Peru3355

Peru3356

Peru3357

Peru3358

Peru3359

Peru3360

Peru3361

Peru3362

Peru3363

Peru3364

Peru3365

Peru3366

Peru3367

Peru3368

Peru3369

Peru3370

Peru3371

Peru3372

Peru3373

Peru3375

Peru3376

Peru3377

Peru3379

Peru3381

Peru3382

Peru3383

Peru3384

Peru3386

Peru3389

Peru3390

Peru3392

Peru3393

Peru3396

Peru3397

Peru3398

Peru3399

Peru3400

Peru3401

Peru3402

Peru3403

Peru3404

Peru3405

Peru3406

Peru3407

Peru3408

Peru3410

Peru3411

Peru3412

Peru3413

Peru3415

Peru3416

Peru3417

Peru3418

Peru3419

Peru3420

Peru3421

Peru3422

Peru3423

Peru3424

Peru3425

Peru3426

Peru3427

Peru3428

Peru3430

Peru3432

Peru3433

Peru3435

Peru3528

Peru3965

Peru3966

Peru3967

Peru3968

Peru3969

Peru3970

Peru3971

Peru3972

Peru3973

Peru3974

Peru3975

Peru3976

Peru3977

Peru3978

Peru3979

Peru3980

Peru3981

Peru3982

Peru3983

Peru3984

Peru3985

Peru3986

Peru3987

Peru3988

Peru3989

Peru3991

Peru3992

Peru3993

Peru3994

Peru3995

Peru3996

Peru3997

Peru3998

Peru3999

Peru4000

Peru4001

Peru4002

Peru4003

Peru4004

Peru4005

Peru4007

Peru4008

Peru4009

Peru4010

Peru4011

Peru4012

Peru4013

Peru4015

Peru4016

Peru4017

Peru4019

Peru4020

Peru4021

Peru4023

Peru4024

Peru4025

Peru4026

Peru4029

Peru4030

Peru4032

Peru4033

Peru4034

Peru4035

Peru4036

Peru4037

Peru4038

Peru4039

Peru4040

Peru4041

Peru4042

Peru4043

Peru4044

Peru4045

Peru4046

Peru4047

Peru4048

Peru4049

Peru4050

Peru4051

Peru4052

Peru4053

Peru4054

Peru4055

Peru4056

Peru4057

Peru4058

Peru4059

Peru4060

Peru4061

Peru4062

Peru4063

Peru4064

Peru4065

Peru4066

Peru4067

Peru4068

Peru4069

Peru4070

Peru4071

Peru4072

Peru4073

Peru4074

Peru4075

Peru4076

Peru4077

Peru4078

Peru4079

Peru4080

Peru4081

Peru4082

Peru4083

Peru4084

Peru4085

Peru4086

Peru4087

Peru4088

Peru4089

Peru4090

Peru4091

Peru4092

Peru4093

Peru4094

Peru4096

Peru4097

Peru4098

Peru4099

Peru4100

Peru4101

Peru4102

Peru4103

Peru4104

Peru4105

Peru4106

Peru4107

Peru4108

Peru4109

Peru4110

Peru4111

Peru4112

Peru4113

Peru4114

Peru4116

Peru4117

Peru4118

Peru4120

Peru4121

Peru4122

Peru4123

Peru4124

Peru4125

Peru4126

Peru4127

Peru4128

Peru4129

Peru4130

Peru4131

Peru4132

Peru4133

Peru4134

Peru4135

Peru4136

Peru4137

Peru4138

Peru4139

Peru4140

Peru4141

Peru4142

Peru4143

Peru4144

Peru4146

Peru4147

Peru4148

Peru4150

Peru4151

Peru4152

Peru4153

Peru4154

Peru4301

Peru4302

Peru4303

Peru4304

Peru4305

Peru4306

Peru4307

Peru4308

Peru4309

Peru4311

Peru4312

Peru4313

Peru4314

Peru4315

Peru4316

Peru4317

Peru4318

Peru4319

Peru4320

Peru4321

Peru4322

Peru4323

Peru4324

Peru4454

Peru4455

Peru4456

Peru4457

Peru4458

Peru4459

Peru4460

Peru4461

Peru4462

Peru4463

Peru4464

Peru4465

Peru4466

Peru4467

Peru4468

Peru4469

Peru4470

Peru4471

Peru4473

Peru4474

Peru4475

Peru4476

Peru4477

Peru4478

Peru4479

Peru4480

Peru4481

Peru4482

Peru4483

Peru4484

Peru4485

Peru4486

Peru4487

Peru4488

Peru4489

Peru4490

Peru4491

Peru4492

Peru4493

Peru4494

Peru4495

Peru4496

Peru4497

Peru4498

Peru4499

Peru4515

Peru4516

Peru4517

Peru4518

Peru4519

Peru4520

Peru4521

Peru4522

Peru4523

Peru4524

Peru4525

Peru4526

Peru4527

Peru4528

Peru4529

Peru4530

Peru4531

Peru4532

Peru4533

Peru4534

Peru4535

Peru4536

Peru4537

Peru4538

Peru4539

Peru4540

Peru4541

Peru4542

Peru4543

Peru4544

Peru4545

Peru4546

Peru4547

Peru4548

Peru4549

Peru4550

Peru4551

Peru4552

Peru4553

Peru4554

Peru4555

Peru4556

Peru4557

Peru4558

Peru4559

Peru4560

Peru4561

Peru4562

Peru4563

Peru4564

Peru4565

Peru4566

Peru4567

Peru4568

Peru4569

Peru4570

Peru4571

Peru4572

Peru4573

Peru4574

Peru4575

Peru4576

Peru4577

Peru4578

Peru4580

Peru4582

Peru4583

Peru4584

Peru4585

Peru4586

Peru4587

Peru4588

Peru4589

Peru4590

Peru4591

Peru4592

Peru4593

Peru4595

Peru4596

Peru4597

Peru4598

Peru4599

Peru4600

Peru4601

Peru4602

Peru4603

Peru4604

Peru4605

Peru4606

Peru4607

Peru4608

Peru4609

Peru4610

Peru4611

Peru4612

Peru4613

Peru4614

Peru4615

Peru4616

Peru4617

Peru4618

Peru4619

Peru4620

Peru4621

Peru4622

Peru4623

Peru4625

Peru4626

Peru4627

Peru4630

Peru4631

Peru4632

Peru4634

Peru4635

Peru4636

Peru4646

Peru4647

Peru4649

Peru4650

Peru4651

Peru4691

Peru4692

Peru4693

Peru4694

Peru4695

Peru4696

Peru4697

Peru4698

Peru4699

Peru4700

Peru4701

Peru4702

Peru4703

Peru4704

Peru4705

Peru4706

Peru4707

Peru4708

Peru4709

Peru4710

Peru4711

Peru4712

Peru4713

Peru4714

Peru4715

Peru4716

Peru4718

Peru4719

Peru4720

Peru4721

Peru4722

Peru4723

Peru4724

Peru4725

Peru4726

Peru4727

Peru4728

Peru4731

Peru4732

Peru4733

Peru4734

Peru4735

Peru4736

Peru4737

Peru4738

Peru4739

Peru4740

Peru4741

Peru4897

Peru4898

Peru4899

Peru4900

Peru4901

Peru4902

Peru4903

Peru4904

Peru4905

Peru4906

Peru4907

Peru4908

Peru4909

Peru4910

Peru4911

Peru4912

Peru4913

Peru4914

Peru4915

Peru4916

Peru4917

Peru4918

Peru4919

Peru4920

Peru4921

Peru4922

Peru4923

Peru4924

Peru4925

Peru4926

Peru4927

Peru4928

Peru4929

Peru4930

Peru4931

Peru4932

Peru4933

Peru4934

Peru4936

Peru4937

Peru4938

Peru4939

Peru4940

Peru4941

Peru4942

Peru4943

Peru4944

Peru4945

Peru4946

Peru4947

Peru4948

Peru4949

Peru4950

Peru4952

Peru4953

Peru4954

Peru4955

Peru4956

Peru4957

Peru4958

Peru4959

Peru4960

Peru4961

Peru4962

Peru4963

Peru4964

Peru4966

Peru4967

Peru4968

Peru4969

Peru4970

Peru4972

Peru4973

Peru4974

Peru4975

Peru4976

Peru4977

Peru4978

Peru4979

Peru4980

Peru4981

Peru4982

Peru4983

Peru4984

Peru4985

Peru4986

Peru4987

Peru4988

Peru4989

Peru4990

Peru4991

Peru4992

Peru4993

Peru4995

Peru4996

Peru4997

Peru4998

Peru4999

Peru5000

Peru5001

Peru5002

Peru5003

Peru5004

Peru5006

Peru5007

Peru5008

Peru5009

Peru5011

Peru5012

Peru5013

Peru5014

Peru5015

Peru5016

Peru5017

Peru5019

Peru5020

Peru5021

Peru5022

Peru5023

Peru5024

Peru5025

Peru5026

Peru5027

Peru5028

Peru5029

Peru5030

Peru5031

Peru5032

Peru5033

Peru5034

Peru5035

Peru5036

Peru5037

Peru5038

Peru5070

Peru5071

Peru5073

Peru5074

Peru5075

Peru5076

Peru5077

Peru5078

Peru5079

Peru5080

Peru5082

Peru5083

Peru5084

Peru5085

Peru5086

Peru5087

Peru5088

Peru5089

Peru5090

Peru5091

Peru5092

Peru5093

Peru5094

Peru5095

Peru5096

Peru5097

Peru5099

Peru5100

Peru5101

Peru5102

Peru5103

Peru5104

Peru5105

Peru5106

Peru5107

Peru5108

Peru5109

Peru5110

Peru5111

Peru5112

Peru5113

Peru5114

Peru5115

Peru5116

Peru5117

Peru5118

Peru5119

Peru5120

Peru5121

Peru5122

Peru5123

Peru5124

Peru5125

Peru5126

Peru5127

Peru5128

Peru5129

Peru5130

Peru5131

Peru5132

Peru5133

Peru5141

Peru5142

Peru5143

Peru5144

Peru5146

Peru5147

Peru5148

Peru5149

Peru5150

Peru5151

Peru5152

Peru5153

Peru5154

Peru5155

Peru5156

Peru5408

Peru5409

Peru5410

Peru5411

Peru5412

Peru5413

Peru5415

Peru5416

Peru5417

Peru5421

Peru5422

Peru5423

Peru5424

Peru5425

Peru5426

Peru5427

Peru5429

Peru5430

Peru5432

Peru5433

Peru5435

Peru5436

Peru5437

Peru5438

Peru5439

Peru5440

Peru5441

Peru5442

Peru5447

Peru5449

Peru5450

Peru5451

Peru5452

Peru5454

Peru5455

SRR057510

SRR057595

SRR057610

SRR057619

SRR057734

SRR057768

SRR057770

SRR057771

SRR058116

SRR058369

SRR058370

SRR058371

SRR058372

SRR058373

SRR058377

SRR058399

SRR058417

SRR1003101

SRR1003102

SRR1003104

SRR1003106

SRR1003107

SRR1003108

SRR1003109

SRR1003110

SRR1003112

SRR1003114

SRR1003130

SRR1003131

SRR1003132

SRR1003133

SRR1010997

SRR1010998

SRR1011449

SRR1011451

SRR1011452

SRR1011453

SRR1011454

SRR1011455

SRR1011456

SRR1011457

SRR1011458

SRR1011459

SRR1011460

SRR1011461

SRR1011462

SRR1011463

SRR1011464

SRR1011465

SRR1011466

SRR1011467

SRR1011470

SRR1011472

SRR1011473

SRR1011474

SRR1011476

SRR1011478

SRR1011479

SRR1011481

SRR1011483

SRR1011485

SRR1011487

SRR1011490

SRR1011491

SRR1013529

SRR1013537

SRR1013538

SRR1013539

SRR1013540

SRR1013541

SRR1013542

SRR1013543

SRR1013544

SRR1013545

SRR1013546

SRR1013547

SRR1013548

SRR1013551

SRR1013552

SRR1013555

SRR1013556

SRR1013557

SRR1013558

SRR1013559

SRR1013560

SRR1013561

SRR1013562

SRR1013563

SRR1013564

SRR1013571

SRR1013572

SRR1013573

SRR1013574

SRR1013579

SRR1013580

SRR1013585

SRR1013586

SRR1013587

SRR1013588

SRR1013593

SRR1013594

SRR1013605

SRR1013606

SRR1013619

SRR1013620

SRR1013623

SRR1013624

SRR1013625

SRR1013626

SRR1013627

SRR1013628

SRR1013631

SRR1013632

SRR1013638

SRR1013639

SRR1013640

SRR1013641

SRR1013642

SRR1013643

SRR1013648

SRR1013649

SRR1013650

SRR1013651

SRR1013652

SRR1013653

SRR1013654

SRR1013655

SRR1013656

SRR1013657

SRR1013666

SRR1013667

SRR1013679

SRR1013680

SRR1019126

SRR1019128

SRR1019129

SRR1019131

SRR1019132

SRR1019133

SRR1019135

SRR1019136

SRR1019137

SRR1019139

SRR1019140

SRR1019141

SRR1019143

SRR1019144

SRR1019145

SRR1019146

SRR1019147

SRR1019148

SRR1019149

SRR1019150

SRR1019151

SRR1019152

SRR1019153

SRR1019154

SRR1019155

SRR1019156

SRR1019158

SRR1019159

SRR1019161

SRR1019162

SRR1019163

SRR1019166

SRR1019168

SRR1019170

SRR1019171

SRR1019172

SRR1019173

SRR1047984

SRR1047988

SRR1048835

SRR1048836

SRR1048839

SRR1048840

SRR1048841

SRR1048842

SRR1048843

SRR1048844

SRR1048845

SRR1048846

SRR1048847

SRR1048850

SRR1048851

SRR1048854

SRR1048855

SRR1048856

SRR1048857

SRR1048858

SRR1048859

SRR1048861

SRR1048862

SRR1048863

SRR1048864

SRR1048871

SRR1048872

SRR1048879

SRR1048880

SRR1048881

SRR1048882

SRR1048962

SRR1048963

SRR1048970

SRR1048971

SRR1048972

SRR1048973

SRR1048978

SRR1048979

SRR1048989

SRR1048990

SRR1048999

SRR1049000

SRR1049001

SRR1049002

SRR1049009

SRR1049010

SRR1049019

SRR1049020

SRR1049024

SRR1049025

SRR1049028

SRR1049029

SRR1049032

SRR1049033

SRR1049034

SRR1049035

SRR1049036

SRR1049037

SRR1049038

SRR1049039

SRR1049042

SRR1049043

SRR1049044

SRR1049045

SRR1049046

SRR1049047

SRR1049048

SRR1049049

SRR1049050

SRR1049051

SRR1049052

SRR1049053

SRR1049054

SRR1049055

SRR1049056

SRR1049057

SRR1049058

SRR1049059

SRR1049060

SRR1049061

SRR1049062

SRR1049063

SRR1049064

SRR1049065

SRR1049066

SRR1049067

SRR1049068

SRR1049069

SRR1049070

SRR1049071

SRR1049072

SRR1049073

SRR1049074

SRR1049075

SRR1049076

SRR1049077

SRR1049557

SRR1049558

SRR1049571

SRR1049572

SRR1049573

SRR1049574

SRR1049575

SRR1049576

SRR1049579

SRR1049580

SRR1049583

SRR1049584

SRR1049585

SRR1049586

SRR1049597

SRR1049598

SRR1049599

SRR1049600

SRR1049603

SRR1049604

SRR1049605

SRR1049606

SRR1049616

SRR1049617

SRR1049620

SRR1049621

SRR1049631

SRR1049632

SRR1049633

SRR1049634

SRR1049639

SRR1049640

SRR1049641

SRR1049642

SRR1049644

SRR1049645

SRR1049646

SRR1049647

SRR1049651

SRR1049652

SRR1049656

SRR1049657

SRR1049658

SRR1049659

SRR1049660

SRR1049661

SRR1049685

SRR1049686

SRR1049687

SRR1049688

SRR1049712

SRR1049713

SRR1049715

SRR1049716

SRR1049717

SRR1049718

SRR1049719

SRR1049720

SRR1049727

SRR1049728

SRR1049729

SRR1049730

SRR1049731

SRR1049732

SRR1049733

SRR1049734

SRR1049737

SRR1049738

SRR1049739

SRR1049740

SRR1049741

SRR1049742

SRR1049749

SRR1049750

SRR1049965

SRR1049966

SRR1049967

SRR1049968

SRR1049969

SRR1049970

SRR1049972

SRR1049973

SRR1062819

SRR1062821

SRR1062822

SRR1062823

SRR1062824

SRR1062825

SRR1062826

SRR1062827

SRR1062828

SRR1062829

SRR1062832

SRR1062833

SRR1062834

SRR1062835

SRR1062836

SRR1062837

SRR1062839

SRR1062840

SRR1062841

SRR1062842

SRR1062843

SRR1062844

SRR1062845

SRR1062846

SRR1062847

SRR1062848

SRR1062849

SRR1062850

SRR1062852

SRR1062853

SRR1062854

SRR1062855

SRR1062856

SRR1062858

SRR1062862

SRR1062866

SRR1062870

SRR1062872

SRR1062875

SRR1062878

SRR1062879

SRR1062881

SRR1062882

SRR1062885

SRR1062887

SRR1062888

SRR1062889

SRR1062890

SRR1062891

SRR1062893

SRR1062894

SRR1062896

SRR1062900

SRR1062901

SRR1062904

SRR1062905

SRR1062907

SRR1062908

SRR1062909

SRR1062910

SRR1062911

SRR1062912

SRR1062913

SRR1062914

SRR1062915

SRR1062916

SRR1062917

SRR1062921

SRR1062922

SRR1062923

SRR1062924

SRR1062929

SRR1062930

SRR1062931

SRR1062932

SRR1062934

SRR1062935

SRR1062936

SRR1062938

SRR1062939

SRR1062940

SRR1140569

SRR1140570

SRR1140571

SRR1140572

SRR1140573

SRR1140577

SRR1140578

SRR1140579

SRR1140580

SRR1140581

SRR1140582

SRR1140583

SRR1140587

SRR1140588

SRR1140589

SRR1140590

SRR1140591

SRR1140592

SRR1140593

SRR1140594

SRR1140596

SRR1140625

SRR1140626

SRR1140637

SRR1140638

SRR1140639

SRR1140640

SRR1140641

SRR1140656

SRR1140657

SRR1140658

SRR1140663

SRR1140664

SRR1140675

SRR1140676

SRR1140682

SRR1140683

SRR1140693

SRR1140694

SRR1140702

SRR1140711

SRR1140717

SRR1140718

SRR1140725

SRR1140737

SRR1140738

SRR1140739

SRR1140752

SRR1140898

SRR1140899

SRR1140900

SRR1140901

SRR1140905

SRR1140916

SRR1140922

SRR1140925

SRR1140926

SRR1140928

SRR1140929

SRR1140930

SRR1140931

SRR1140932

SRR1140934

SRR1140935

SRR1140936

SRR1140938

SRR1140940

SRR1140941

SRR1140943

SRR1140944

SRR1140945

SRR1140946

SRR1140947

SRR1140948

SRR1140949

SRR1140950

SRR1140951

SRR1140952

SRR1140953

SRR1140954

SRR1140955

SRR1140956

SRR1140958

SRR1140959

SRR1140960

SRR1140961

SRR1140964

SRR1140965

SRR1140966

SRR1144725

SRR1144737

SRR1144749

SRR1144757

SRR1144760

SRR1144766

SRR1144768

SRR1144772

SRR1144776

SRR1144780

SRR1144783

SRR1144787

SRR1144790

SRR1144796

SRR1144802

SRR1144805

SRR1144807

SRR1144813

SRR1144814

SRR1144817

SRR1144820

SRR1144821

SRR1145855

SRR1145859

SRR1146268

SRR1146277

SRR1146290

SRR1146293

SRR1146296

SRR1146309

SRR1146313

SRR1146316

SRR1146329

SRR1146339

SRR1146347

SRR1146373

SRR1146378

SRR1146384

SRR1146413

SRR1146414

SRR1146421

SRR1158874

SRR1158890

SRR1158898

SRR1158907

SRR1158923

SRR1158931

SRR1158939

SRR1158943

SRR1158946

SRR1158950

SRR1158990

SRR1158998

SRR1159002

SRR1159003

SRR1159005

SRR1159006

SRR1159029

SRR1159034

SRR1159038

SRR1159044

SRR1159052

SRR1159053

SRR1159075

SRR1159076

SRR1159083

SRR1159108

SRR1159121

SRR1159122

SRR1159126

SRR1159129

SRR1159150

SRR1159154

SRR1159167

SRR1159171

SRR1159175

SRR1159180

SRR1159204

SRR1159237

SRR1159245

SRR1159261

SRR1159279

SRR1159283

SRR1159290

SRR1159294

SRR1159298

SRR1159299

SRR1159301

SRR1159303

SRR1159308

SRR1159309

SRR1159310

SRR1159338

SRR1159346

SRR1159350

SRR1159351

SRR1159359

SRR1159360

SRR1159362

SRR1159366

SRR1159369

SRR1159370

SRR1159377

SRR1159384

SRR1159393

SRR1159521

SRR1159526

SRR1159661

SRR1159687

SRR1159700

SRR1159713

SRR1159721

SRR1159738

SRR1159760

SRR1159779

SRR1159790

SRR1159818

SRR1159861

SRR1159895

SRR1159954

SRR1159959

SRR1159977

SRR1159986

SRR1162468

SRR1162469

SRR1162470

SRR1162471

SRR1162473

SRR1162474

SRR1162475

SRR1162476

SRR1162478

SRR1162479

SRR1162480

SRR1162481

SRR1162483

SRR1162485

SRR1162494

SRR1162495

SRR1162498

SRR1162502

SRR1162504

SRR1162505

SRR1162508

SRR1162509

SRR1162513

SRR1162518

SRR1162521

SRR1162531

SRR1162532

SRR1162533

SRR1162534

SRR1162535

SRR1162536

SRR1162537

SRR1162541

SRR1162542

SRR1162689

SRR1162691

SRR1162694

SRR1162695

SRR1162699

SRR1162700

SRR1162701

SRR1162704

SRR1162705

SRR1162707

SRR1162708

SRR1162709

SRR1162710

SRR1162716

SRR1162720

SRR1162721

SRR1162725

SRR1162726

SRR1162727

SRR1162728

SRR1162731

SRR1162732

SRR1162737

SRR1162738

SRR1162739

SRR1162741

SRR1162745

SRR1162746

SRR1162747

SRR1162749

SRR1162750

SRR1162753

SRR1162754

SRR1162755

SRR1162758

SRR1162763

SRR1162764

SRR1162765

SRR1162770

SRR1162773

SRR1162774

SRR1162775

SRR1162778

SRR1162788

SRR1162789

SRR1162792

SRR1162793

SRR1162795

SRR1162796

SRR1162801

SRR1162802

SRR1162803

SRR1162806

SRR1162807

SRR1162810

SRR1162811

SRR1162812

SRR1162814

SRR1162815

SRR1162817

SRR1162818

SRR1162819

SRR1162822

SRR1162823

SRR1162825

SRR1162828

SRR1162829

SRR1162833

SRR1162834

SRR1162835

SRR1162836

SRR1162840

SRR1162842

SRR1162843

SRR1162845

SRR1162846

SRR1162847

SRR1162850

SRR1162851

SRR1162852

SRR1162853

SRR1162856

SRR1162860

SRR1162861

SRR1162862

SRR1162863

SRR1162866

SRR1162867

SRR1162868

SRR1162870

SRR1162871

SRR1162872

SRR1162873

SRR1162874

SRR1162875

SRR1162876

SRR1162877

SRR1162878

SRR1162879

SRR1162880

SRR1162881

SRR1162883

SRR1162884

SRR1162885

SRR1162886

SRR1162887

SRR1162888

SRR1162890

SRR1162891

SRR1162893

SRR1162894

SRR1162895

SRR1162897

SRR1162898

SRR1162899

SRR1162900

SRR1162947

SRR1162948

SRR1162952

SRR1162953

SRR1162954

SRR1162956

SRR1162957

SRR1162958

SRR1162959

SRR1162961

SRR1162962

SRR1162966

SRR1162967

SRR1162968

SRR1162969

SRR1162971

SRR1162972

SRR1162977

SRR1162980

SRR1162986

SRR1162991

SRR1162992

SRR1162995

SRR1162996

SRR1162997

SRR1162998

SRR1163001

SRR1163002

SRR1163003

SRR1163004

SRR1163006

SRR1163007

SRR1163008

SRR1163014

SRR1163015

SRR1163016

SRR1163017

SRR1163021

SRR1163022

SRR1163023

SRR1163024

SRR1163025

SRR1163029

SRR1163030

SRR1163031

SRR1163032

SRR1163037

SRR1163038

SRR1163039

SRR1163040

SRR1163041

SRR1163046

SRR1163047

SRR1163050

SRR1163051

SRR1163052

SRR1163073

SRR1163075

SRR1163076

SRR1163077

SRR1163078

SRR1163079

SRR1163080

SRR1163081

SRR1163085

SRR1163086

SRR1163087

SRR1163089

SRR1163090

SRR1163092

SRR1163093

SRR1163094

SRR1163096

SRR1163097

SRR1163101

SRR1163102

SRR1163103

SRR1163104

SRR1163106

SRR1163107

SRR1163108

SRR1163109

SRR1163110

SRR1163113

SRR1163114

SRR1163115

SRR1163116

SRR1163117

SRR1163121

SRR1163122

SRR1163123

SRR1163124

SRR1163126

SRR1163127

SRR1163128

SRR1163129

SRR1163135

SRR1163137

SRR1163139

SRR1163140

SRR1163142

SRR1163145

SRR1163146

SRR1163166

SRR1163167

SRR1163168

SRR1163171

SRR1163173

SRR1163174

SRR1163175

SRR1163176

SRR1163177

SRR1163178

SRR1163179

SRR1163184

SRR1163185

SRR1163189

SRR1163190

SRR1163194

SRR1163195

SRR1163196

SRR1163198

SRR1163199

SRR1163200

SRR1163201

SRR1163202

SRR1163203

SRR1163204

SRR1163206

SRR1163207

SRR1163208

SRR1163211

SRR1163212

SRR1163213

SRR1163215

SRR1163216

SRR1163218

SRR1163219

SRR1163222

SRR1163223

SRR1163229

SRR1163233

SRR1163234

SRR1163236

SRR1163239

SRR1163286

SRR1163287

SRR1163288

SRR1163290

SRR1163291

SRR1163293

SRR1163294

SRR1163296

SRR1163298

SRR1163299

SRR1163302

SRR1163303

SRR1163304

SRR1163305

SRR1163306

SRR1163309

SRR1163310

SRR1163313

SRR1163314

SRR1163315

SRR1163317

SRR1163318

SRR1163319

SRR1163320

SRR1163321

SRR1163325

SRR1163326

SRR1163327

SRR1163329

SRR1163330

SRR1163332

SRR1163336

SRR1163337

SRR1163338

SRR1163339

SRR1163341

SRR1163342

SRR1163344

SRR1163345

SRR1163347

SRR1163348

SRR1163349

SRR1163351

SRR1163353

SRR1163354

SRR1163355

SRR1163357

SRR1163358

SRR1163359

SRR1163361

SRR1163363

SRR1163365

SRR1163366

SRR1163367

SRR1163368

SRR1163369

SRR1163372

SRR1163373

SRR1163376

SRR1163379

SRR1163380

SRR1163381

SRR1163382

SRR1163385

SRR1163386

SRR1163387

SRR1163388

SRR1163392

SRR1163393

SRR1163394

SRR1163397

SRR1163398

SRR1163399

SRR1163400

SRR1163404

SRR1163405

SRR1163406

SRR1163407

SRR1163408

SRR1163411

SRR1163412

SRR1163413

SRR1163414

SRR1163415

SRR1163416

SRR1163417

SRR1163418

SRR1163419

SRR1163421

SRR1163422

SRR1163423

SRR1163424

SRR1163425

SRR1163429

SRR1163430

SRR1163431

SRR1163432

SRR1163447

SRR1163448

SRR1163452

SRR1163459

SRR1163462

SRR1163464

SRR1163465

SRR1163473

SRR1163478

SRR1163480

SRR1163483

SRR1163488

SRR1163490

SRR1163493

SRR1165210

SRR1165211

SRR1165212

SRR1165213

SRR1165215

SRR1165216

SRR1165217

SRR1165219

SRR1165220

SRR1165221

SRR1165222

SRR1165224

SRR1165225

SRR1165226

SRR1165227

SRR1165228

SRR1165230

SRR1165231

SRR1165232

SRR1165237

SRR1165240

SRR1165241

SRR1165242

SRR1165244

SRR1165245

SRR1165246

SRR1165249

SRR1165250

SRR1165253

SRR1165254

SRR1165256

SRR1165258

SRR1165259

SRR1165262

SRR1165263

SRR1165264

SRR1165266

SRR1165267

SRR1165270

SRR1165272

SRR1165274

SRR1165275

SRR1165276

SRR1165277

SRR1165278

SRR1165279

SRR1165280

SRR1165281

SRR1165290

SRR1165293

SRR1165294

SRR1165297

SRR1165298

SRR1165302

SRR1165305

SRR1165309

SRR1165310

SRR1165314

SRR1165318

SRR1165323

SRR1165326

SRR1165327

SRR1165330

SRR1165332

SRR1165335

SRR1165336

SRR1165339

SRR1165340

SRR1165366

SRR1165369

SRR1165372

SRR1165373

SRR1165376

SRR1165377

SRR1165379

SRR1165394

SRR1165398

SRR1165399

SRR1165403

SRR1165415

SRR1165418

SRR1165419

SRR1165426

SRR1165427

SRR1165431

SRR1165432

SRR1165437

SRR1165440

SRR1165444

SRR1165445

SRR1165446

SRR1165450

SRR1165453

SRR1165456

SRR1165457

SRR1165461

SRR1165467

SRR1165468

SRR1165473

SRR1165474

SRR1165477

SRR1165480

SRR1165490

SRR1165491

SRR1165495

SRR1165498

SRR1165499

SRR1165500

SRR1165502

SRR1165504

SRR1165508

SRR1165509

SRR1165512

SRR1165515

SRR1165519

SRR1165521

SRR1165523

SRR1165524

SRR1165525

SRR1165530

SRR1165532

SRR1165533

SRR1165535

SRR1165536

SRR1165537

SRR1165539

SRR1165544

SRR1165546

SRR1165549

SRR1165550

SRR1165553

SRR1165554

SRR1165557

SRR1165559

SRR1165564

SRR1165566

SRR1165573

SRR1165574

SRR1165577

SRR1165580

SRR1165582

SRR1165583

SRR1165585

SRR1165588

SRR1165590

SRR1165591

SRR1165592

SRR1165595

SRR1165597

SRR1165599

SRR1165600

SRR1165603

SRR1165604

SRR1165606

SRR1166082

SRR1166083

SRR1166084

SRR1166086

SRR1166087

SRR1166088

SRR1166089

SRR1166090

SRR1166091

SRR1166092

SRR1166093

SRR1166094

SRR1166095

SRR1166096

SRR1166097

SRR1166098

SRR1166099

SRR1166100

SRR1166101

SRR1166103

SRR1166104

SRR1166105

SRR1166106

SRR1166107

SRR1166108

SRR1166109

SRR1166111

SRR1166113

SRR1166115

SRR1166116

SRR1166117

SRR1166118

SRR1166120

SRR1166121

SRR1166122

SRR1166125

SRR1166126

SRR1166130

SRR1166131

SRR1166132

SRR1166133

SRR1166134

SRR1166135

SRR1166136

SRR1166137

SRR1166138

SRR1166142

SRR1166143

SRR1166144

SRR1166145

SRR1166146

SRR1166147

SRR1166151

SRR1166153

SRR1166154

SRR1166155

SRR1166157

SRR1166158

SRR1166160

SRR1166162

SRR1166163

SRR1166164

SRR1166166

SRR1166170

SRR1166171

SRR1166174

SRR1166175

SRR1166176

SRR1166177

SRR1166178

SRR1166179

SRR1166180

SRR1166187

SRR1166188

SRR1166189

SRR1166190

SRR1166193

SRR1166195

SRR1166201

SRR1166208

SRR1166220

SRR1166227

SRR1166228

SRR1166239

SRR1166253

SRR1166261

SRR1166267

SRR1166268

SRR1166276

SRR1166282

SRR1166283

SRR1166296

SRR1166297

SRR1166300

SRR1166301

SRR1166302

SRR1166303

SRR1166304

SRR1166305

SRR1166307

SRR1166312

SRR1166313

SRR1166315

SRR1166316

SRR1166318

SRR1166319

SRR1166321

SRR1166322

SRR1166324

SRR1166325

SRR1166327

SRR1166328

SRR1166329

SRR1166330

SRR1166331

SRR1166336

SRR1166337

SRR1166341

SRR1166707

SRR1166708

SRR1166709

SRR1166710

SRR1166711

SRR1166712

SRR1166713

SRR1166714

SRR1166715

SRR1166722

SRR1166804

SRR1166805

SRR1166807

SRR1166808

SRR1166809

SRR1166812

SRR1166813

SRR1166814

SRR1166815

SRR1166816

SRR1166817

SRR1166818

SRR1166819

SRR1166820

SRR1166822

SRR1166823

SRR1166825

SRR1166826

SRR1166827

SRR1166828

SRR1166829

SRR1166864

SRR1166865

SRR1166866

SRR1166867

SRR1166868

SRR1166869

SRR1166870

SRR1166871

SRR1166873

SRR1166874

SRR1166875

SRR1166877

SRR1166878

SRR1166879

SRR1166908

SRR1166909

SRR1166910

SRR1166911

SRR1166913

SRR1166914

SRR1166915

SRR1166916

SRR1166917

SRR1166918

SRR1166919

SRR1166920

SRR1166921

SRR1166922

SRR1166923

SRR1166925

SRR1166926

SRR1166927

SRR1166928

SRR1166929

SRR1166930

SRR1166931

SRR1166932

SRR1166933

SRR1166934

SRR1166935

SRR1166936

SRR1166937

SRR1166938

SRR1167058

SRR1167061

SRR1167067

SRR1167069

SRR1167073

SRR1167075

SRR1167079

SRR1167080

SRR1167081

SRR1167084

SRR1167087

SRR1167090

SRR1167097

SRR1167099

SRR1167100

SRR1167113

SRR1167114

SRR1167117

SRR1167120

SRR1167123

SRR1167124

SRR1167128

SRR1167130

SRR1167131

SRR1167134

SRR1167138

SRR1167144

SRR1167147

SRR1167151

SRR1167152

SRR1167166

SRR1167167

SRR1167168

SRR1167169

SRR1167170

SRR1167171

SRR1167172

SRR1167174

SRR1167175

SRR1167179

SRR1167180

SRR1167181

SRR1167182

SRR1167184

SRR1167185

SRR1167244

SRR1167245

SRR1167248

SRR1167249

SRR1167250

SRR1167251

SRR1167252

SRR1167253

SRR1167254

SRR1167259

SRR1167260

SRR1167261

SRR1167263

SRR1167265

SRR1167266

SRR1167267

SRR1167272

SRR1167275

SRR1167276

SRR1167278

SRR1167280

SRR1167281

SRR1167283

SRR1167285

SRR1167286

SRR1167287

SRR1167288

SRR1167289

SRR1167290

SRR1167292

SRR1167293

SRR1167294

SRR1167295

SRR1167296

SRR1167299

SRR1167300

SRR1167303

SRR1167304

SRR1167305

SRR1167306

SRR1167308

SRR1167309

SRR1167312

SRR1167313

SRR1167314

SRR1167315

SRR1167316

SRR1167317

SRR1167318

SRR1167319

SRR1167320

SRR1167322

SRR1167323

SRR1168954

SRR1168958

SRR1168959

SRR1168960

SRR1168961

SRR1168962

SRR1168963

SRR1168964

SRR1168965

SRR1168967

SRR1168969

SRR1168970

SRR1168971

SRR1168972

SRR1168973

SRR1168974

SRR1168975

SRR1168976

SRR1168977

SRR1168978

SRR1168979

SRR1168981

SRR1168982

SRR1168983

SRR1168984

SRR1168985

SRR1168987

SRR1168988

SRR1168989

SRR1168990

SRR1168992

SRR1168993

SRR1168994

SRR1168995

SRR1168997

SRR1168998

SRR1169000

SRR1169001

SRR1169004

SRR1169005

SRR1169006

SRR1169009

SRR1169010

SRR1169011

SRR1169013

SRR1169014

SRR1169015

SRR1169016

SRR1169018

SRR1169019

SRR1169020

SRR1169021

SRR1169022

SRR1169023

SRR1169025

SRR1169028

SRR1169029

SRR1169035

SRR1169036

SRR1169037

SRR1169039

SRR1169040

SRR1169041

SRR1169042

SRR1169043

SRR1169045

SRR1169047

SRR1169048

SRR1169049

SRR1169050

SRR1169051

SRR1169052

SRR1169053

SRR1169054

SRR1169055

SRR1169056

SRR1169060

SRR1169061

SRR1169062

SRR1169065

SRR1169066

SRR1169068

SRR1169070

SRR1169071

SRR1169072

SRR1169073

SRR1169074

SRR1169075

SRR1169076

SRR1169077

SRR1169078

SRR1169079

SRR1169080

SRR1169081

SRR1169082

SRR1169083

SRR1169084

SRR1169085

SRR1169087

SRR1169088

SRR1169089

SRR1169090

SRR1169092

SRR1169093

SRR1169094

SRR1169095

SRR1169096

SRR1169097

SRR1169099

SRR1169100

SRR1169101

SRR1169102

SRR1169105

SRR1169106

SRR1169108

SRR1169109

SRR1169110

SRR1169111

SRR1169112

SRR1169113

SRR1169115

SRR1169117

SRR1169120

SRR1169123

SRR1169124

SRR1169125

SRR1169127

SRR1169129

SRR1169130

SRR1169131

SRR1169133

SRR1169134

SRR1169135

SRR1169142

SRR1169146

SRR1169147

SRR1169148

SRR1169149

SRR1169151

SRR1169152

SRR1169153

SRR1169155

SRR1169157

SRR1169158

SRR1169159

SRR1169160

SRR1169162

SRR1169163

SRR1169164

SRR1169165

SRR1169166

SRR1169169

SRR1169170

SRR1169171

SRR1169172

SRR1169173

SRR1169175

SRR1169176

SRR1169177

SRR1169178

SRR1169179

SRR1169181

SRR1169182

SRR1169185

SRR1169188

SRR1169189

SRR1169190

SRR1169192

SRR1169193

SRR1169197

SRR1169199

SRR1169201

SRR1169202

SRR1169205

SRR1169206

SRR1169207

SRR1169208

SRR1169209

SRR1169211

SRR1169212

SRR1169213

SRR1169214

SRR1169217

SRR1169220

SRR1169221

SRR1169222

SRR1169223

SRR1169224

SRR1169226

SRR1169227

SRR1169228

SRR1169229

SRR1169240

SRR1169242

SRR1169243

SRR1169244

SRR1169245

SRR1169246

SRR1169250

SRR1169252

SRR1169253

SRR1169256

SRR1169262

SRR1169263

SRR1169264

SRR1169265

SRR1169270

SRR1169271

SRR1169275

SRR1169282

SRR1169283

SRR1169287

SRR1169288

SRR1169289

SRR1169292

SRR1169293

SRR1169295

SRR1169307

SRR1169308

SRR1169309

SRR1169310

SRR1169314

SRR1169315

SRR1169319

SRR1169323

SRR1169331

SRR1169458

SRR1169461

SRR1169462

SRR1169463

SRR1169465

SRR1169467

SRR1169470

SRR1169471

SRR1169472

SRR1169475

SRR1169476

SRR1169477

SRR1169482

SRR1169483

SRR1169484

SRR1169487

SRR1169488

SRR1169489

SRR1169490

SRR1169491

SRR1169494

SRR1169495

SRR1169497

SRR1169499

SRR1169500

SRR1169505

SRR1169508

SRR1169511

SRR1169512

SRR1169513

SRR1169517

SRR1169518

SRR1169521

SRR1169522

SRR1169527

SRR1169528

SRR1169529

SRR1169531

SRR1169533

SRR1169535

SRR1169536

SRR1169537

SRR1169538

SRR1169539

SRR1169542

SRR1169543

SRR1169544

SRR1169547

SRR1169549

SRR1169553

SRR1169554

SRR1169557

SRR1169559

SRR1169563

SRR1169564

SRR1169565

SRR1169568

SRR1169570

SRR1169573

SRR1169575

SRR1169576

SRR1169579

SRR1169580

SRR1169585

SRR1169586

SRR1169589

SRR1169590

SRR1169624

SRR1172022

SRR1172027

SRR1172033

SRR1172034

SRR1172035

SRR1172044

SRR1172054

SRR1172059

SRR1172060

SRR1172061

SRR1172064

SRR1172065

SRR1172068

SRR1172072

SRR1172074

SRR1172075

SRR1172079

SRR1172080

SRR1172081

SRR1172082

SRR1172085

SRR1172087

SRR1172089

SRR1172091

SRR1172094

SRR1172096

SRR1172100

SRR1172101

SRR1172102

SRR1172105

SRR1172106

SRR1172107

SRR1172108

SRR1172117

SRR1172118

SRR1172119

SRR1172121

SRR1172122

SRR1172123

SRR1172124

SRR1172125

SRR1172129

SRR1172130

SRR1172131

SRR1172133

SRR1172134

SRR1172136

SRR1172139

SRR1172140

SRR1172141

SRR1172144

SRR1172147

SRR1172150

SRR1172151

SRR1172152

SRR1172156

SRR1172157

SRR1172159

SRR1172163

SRR1172164

SRR1172165

SRR1172175

SRR1172176

SRR1172181

SRR1172182

SRR1172183

SRR1172184

SRR1172186

SRR1172188

SRR1172189

SRR1172200

SRR1172201

SRR1172202

SRR1172203

SRR1172207

SRR1172208

SRR1172209

SRR1172210

SRR1172220

SRR1172221

SRR1172225

SRR1172226

SRR1172227

SRR1172228

SRR1172231

SRR1172232

SRR1172233

SRR1172239

SRR1172240

SRR1172241

SRR1172242

SRR1172246

SRR1172247

SRR1172251

SRR1172252

SRR1172258

SRR1172259

SRR1172260

SRR1172261

SRR1172264

SRR1172265

SRR1172266

SRR1172272

SRR1172273

SRR1172274

SRR1172276

SRR1172280

SRR1172281

SRR1172286

SRR1172287

SRR1172288

SRR1172289

SRR1172292

SRR1172294

SRR1172298

SRR1172299

SRR1172300

SRR1172304

SRR1172305

SRR1172308

SRR1172309

SRR1172311

SRR1172313

SRR1172314

SRR1172315

SRR1172316

SRR1172320

SRR1172321

SRR1172323

SRR1172330

SRR1172335

SRR1172336

SRR1172341

SRR1172342

SRR1172345

SRR1172347

SRR1172348

SRR1172351

SRR1172352

SRR1172353

SRR1172358

SRR1172364

SRR1172366

SRR1172367

SRR1172387

SRR1172393

SRR1172405

SRR1172406

SRR1172410

SRR1172411

SRR1172412

SRR1172438

SRR1172440

SRR1172443

SRR1172449

SRR1172452

SRR1172455

SRR1172711

SRR1172715

SRR1172716

SRR1172718

SRR1172720

SRR1172721

SRR1172723

SRR1172724

SRR1172727

SRR1172736

SRR1172737

SRR1172738

SRR1172743

SRR1172744

SRR1172747

SRR1172748

SRR1172752

SRR1172773

SRR1172774

SRR1172776

SRR1172779

SRR1172780

SRR1172783

SRR1172786

SRR1172787

SRR1172788

SRR1172792

SRR1172793

SRR1172797

SRR1172799

SRR1172800

SRR1172801

SRR1172804

SRR1172813

SRR1172814

SRR1172818

SRR1172821

SRR1172822

SRR1172823

SRR1172825

SRR1172826

SRR1172827

SRR1172828

SRR1172829

SRR1172831

SRR1172834

SRR1172835

SRR1172836

SRR1172843

SRR1172845

SRR1172849

SRR1172852

SRR1172853

SRR1172854

SRR1172855

SRR1172856

SRR1172858

SRR1172860

SRR1172862

SRR1172863

SRR1172869

SRR1172870

SRR1172872

SRR1172875

SRR1172876

SRR1172879

SRR1172880

SRR1172881

SRR1172882

SRR1172886

SRR1172887

SRR1172888

SRR1172898

SRR1172899

SRR1172900

SRR1172901

SRR1172903

SRR1172905

SRR1172906

SRR1172907

SRR1172914

SRR1172923

SRR1172928

SRR1172930

SRR1172931

SRR1172935

SRR1172939

SRR1172942

SRR1172944

SRR1172950

SRR1172955

SRR1172959

SRR1172961

SRR1172965

SRR1172971

SRR1172977

SRR1172978

SRR1172979

SRR1172985

SRR1172986

SRR1172989

SRR1172990

SRR1172991

SRR1172992

SRR1172993

SRR1172999

SRR1173004

SRR1173005

SRR1173009

SRR1173010

SRR1173012

SRR1173015

SRR1173019

SRR1173021

SRR1173022

SRR1173023

SRR1173027

SRR1173028

SRR1173029

SRR1173031

SRR1173032

SRR1173033

SRR1173035

SRR1173038

SRR1173043

SRR1173044

SRR1173046

SRR1173054

SRR1173056

SRR1173060

SRR1173061

SRR1173068

SRR1173072

SRR1173076

SRR1173077

SRR1173078

SRR1173080

SRR1173082

SRR1173083

SRR1173084

SRR1173085

SRR1173087

SRR1173088

SRR1173092

SRR1173093

SRR1173095

SRR1173098

SRR1173104

SRR1173107

SRR1173110

SRR1173112

SRR1173116

SRR1173122

SRR1173123

SRR1173124

SRR1173127

SRR1173129

SRR1173130

SRR1173131

SRR1173132

SRR1173134

SRR1173135

SRR1173136

SRR1173137

SRR1173140

SRR1173142

SRR1173144

SRR1173145

SRR1173147

SRR1173149

SRR1173150

SRR1173152

SRR1173154

SRR1173155

SRR1173156

SRR1173157

SRR1173158

SRR1173159

SRR1173160

SRR1173161

SRR1173167

SRR1173168

SRR1173171

SRR1173172

SRR1173173

SRR1173175

SRR1173178

SRR1173179

SRR1173181

SRR1173182

SRR1173186

SRR1173187

SRR1173191

SRR1173192

SRR1173193

SRR1173198

SRR1173200

SRR1173202

SRR1173206

SRR1173207

SRR1173209

SRR1173211

SRR1173212

SRR1173215

SRR1173216

SRR1173217

SRR1173219

SRR1173220

SRR1173226

SRR1173248

SRR1173249

SRR1173251

SRR1173252

SRR1173253

SRR1173254

SRR1173257

SRR1173258

SRR1173260

SRR1173261

SRR1173264

SRR1173266

SRR1173267

SRR1173270

SRR1173271

SRR1173275

SRR1173276

SRR1173282

SRR1173283

SRR1173285

SRR1173286

SRR1173288

SRR1173289

SRR1173290

SRR1173294

SRR1173295

SRR1173296

SRR1173299

SRR1173300

SRR1173301

SRR1173305

SRR1173306

SRR1173307

SRR1173308

SRR1173312

SRR1173316

SRR1173317

SRR1173323

SRR1173325

SRR1173326

SRR1173327

SRR1173328

SRR1173329

SRR1173331

SRR1173332

SRR1173333

SRR1173336

SRR1173337

SRR1173338

SRR1173339

SRR1173346

SRR1173347

SRR1173349

SRR1173350

SRR1173351

SRR1173353

SRR1173356

SRR1173357

SRR1173358

SRR1173364

SRR1173366

SRR1173371

SRR1173374

SRR1173378

SRR1173381

SRR1173382

SRR1173386

SRR1173388

SRR1173389

SRR1173390

SRR1173391

SRR1173392

SRR1173393

SRR1173394

SRR1173395

SRR1173396

SRR1173397

SRR1173402

SRR1173403

SRR1173404

SRR1173405

SRR1173406

SRR1173407

SRR1173418

SRR1173419

SRR1173422

SRR1173423

SRR1173424

SRR1173425

SRR1173426

SRR1173431

SRR1173432

SRR1173436

SRR1173437

SRR1173440

SRR1173442

SRR1173443

SRR1173446

SRR1173448

SRR1173454

SRR1173455

SRR1173456

SRR1173483

SRR1173486

SRR1173487

SRR1173489

SRR1173490

SRR1173491

SRR1173493

SRR1173494

SRR1173499

SRR1173500

SRR1173503

SRR1173516

SRR1173517

SRR1173522

SRR1173526

SRR1173528

SRR1173531

SRR1173532

SRR1173537

SRR1173538

SRR1173542

SRR1173543

SRR1173546

SRR1173547

SRR1173548

SRR1173551

SRR1173552

SRR1173555

SRR1173560

SRR1173569

SRR1173572

SRR1173574

SRR1173575

SRR1173576

SRR1173580

SRR1173582

SRR1173584

SRR1173586

SRR1173595

SRR1173600

SRR1173607

SRR1173611

SRR1173612

SRR1173623

SRR1173624

SRR1173628

SRR1173635

SRR1173637

SRR1173638

SRR1173640

SRR1173643

SRR1173644

SRR1173648

SRR1173650

SRR1173655

SRR1173659

SRR1173660

SRR1173673

SRR1173674

SRR1173675

SRR1173677

SRR1173680

SRR1173683

SRR1173684

SRR1173693

SRR1173695

SRR1173696

SRR1173707

SRR1173718

SRR1173722

SRR1173723

SRR1173728

SRR1173730

SRR1173731

SRR1173735

SRR1173736

SRR1173737

SRR1173741

SRR1173742

SRR1173749

SRR1173750

SRR1173765

SRR1173769

SRR1173771

SRR1173774

SRR1173776

SRR1173782

SRR1173783

SRR1173784

SRR1173786

SRR1173787

SRR1173792

SRR1173793

SRR1173795

SRR1173801

SRR1173802

SRR1173803

SRR1173807

SRR1173808

SRR1173813

SRR1173814

SRR1173815

SRR1173820

SRR1173834

SRR1173835

SRR1173836

SRR1173837

SRR1173838

SRR1173839

SRR1173842

SRR1173846

SRR1173847

SRR1173849

SRR1173850

SRR1173851

SRR1173852

SRR1173855

SRR1173859

SRR1173860

SRR1173868

SRR1173869

SRR1173872

SRR1173875

SRR1173876

SRR1174278

SRR1174279

SRR1174280

SRR1174286

SRR1174292

SRR1174296

SRR1174305

SRR1174307

SRR1174308

SRR1174309

SRR1174323

SRR1174328

SRR1174822

SRR1174823

SRR1174825

SRR1174826

SRR1174827

SRR1174829

SRR1174830

SRR1174831

SRR1174832

SRR1174836

SRR1174838

SRR1174840

SRR1174841

SRR1174850

SRR1174857

SRR1174863

SRR1174894

SRR1174896

SRR1175023

SRR1175027

SRR1175028

SRR1175030

SRR1175032

SRR1175034

SRR1175035

SRR1175036

SRR1175038

SRR1175041

SRR1175043

SRR1175044

SRR1175045

SRR1175054

SRR1175055

SRR1175058

SRR1175061

SRR1175062

SRR1175063

SRR1175065

SRR1175071

SRR1175072

SRR1175078

SRR1175079

SRR1175082

SRR1175083

SRR1175088

SRR1175089

SRR1175091

SRR1175092

SRR1175095

SRR1175100

SRR1175108

SRR1175109

SRR1175113

SRR1175116

SRR1175117

SRR1175118

SRR1175123

SRR1175128

SRR1175134

SRR1175136

SRR1175141

SRR1175145

SRR1175146

SRR1175155

SRR1175156

SRR1175160

SRR1175169

SRR1175170

SRR1175174

SRR1175300

SRR1175301

SRR1175303

SRR1175305

SRR1175307

SRR1175325

SRR1175329

SRR1175330

SRR1175331

SRR1175337

SRR1175338

SRR1175342

SRR1175343

SRR1175346

SRR1175347

SRR1175350

SRR1175351

SRR1175354

SRR1175355

SRR1175358

SRR1175359

SRR1175439

SRR1175440

SRR1175469

SRR1175470

SRR1175478

SRR1175483

SRR1175497

SRR1175498

SRR1175510

SRR1175511

SRR1175516

SRR1175517

SRR1175518

SRR1175521

SRR1175522

SRR1175523

SRR1175524

SRR1180139

SRR1180142

SRR1180146

SRR1180152

SRR1180156

SRR1180157

SRR1180159

SRR1180161

SRR1180166

SRR1180167

SRR1180168

SRR1180173

SRR1180180

SRR1180181

SRR1180184

SRR1180185

SRR1180186

SRR1180189

SRR1180190

SRR1180195

SRR1180200

SRR1180201

SRR1180210

SRR1180212

SRR1180217

SRR1180218

SRR1180219

SRR1180227

SRR1180230

SRR1180231

SRR1180232

SRR1180233

SRR1180238

SRR1180244

SRR1180245

SRR1180246

SRR1180253

SRR1180254

SRR1180257

SRR1180258

SRR1180259

SRR1180262

SRR1180267

SRR1180268

SRR1180272

SRR1180273

SRR1180276

SRR1180280

SRR1180281

SRR1180282

SRR1180283

SRR1180284

SRR1180286

SRR1180287

SRR1180289

SRR1180293

SRR1180299

SRR1180301

SRR1180302

SRR1180308

SRR1180313

SRR1180314

SRR1180315

SRR1180316

SRR1180318

SRR1180320

SRR1180322

SRR1180327

SRR1180328

SRR1180329

SRR1180330

SRR1180336

SRR1180338

SRR1180339

SRR1180341

SRR1180343

SRR1180346

SRR1180349

SRR1180352

SRR1180353

SRR1180356

SRR1180357

SRR1180358

SRR1180359

SRR1180362

SRR1180364

SRR1180368

SRR1180369

SRR1180371

SRR1180372

SRR1180376

SRR1180378

SRR1180379

SRR1180384

SRR1180385

SRR1180387

SRR1180388

SRR1180392

SRR1180395

SRR1180396

SRR1180397

SRR1180398

SRR1180400

SRR1180402

SRR1180403

SRR1180404

SRR1180407

SRR1180415

SRR1180417

SRR1180418

SRR1180419

SRR1180421

SRR1180422

SRR1180424

SRR1180425

SRR1180427

SRR1180428

SRR1180429

SRR1180432

SRR1180433

SRR1180434

SRR1180435

SRR1180436

SRR1180437

SRR1180439

SRR1180441

SRR1180442

SRR1180443

SRR1180445

SRR1180447

SRR1180449

SRR1180458

SRR1180770

SRR1180921

SRR1180922

SRR1180923

SRR1180924

SRR1180925

SRR1180935

SRR1180936

SRR1180937

SRR1180938

SRR1180939

SRR1180940

SRR1180944

SRR1180945

SRR1180950

SRR1180951

SRR1180952

SRR1180957

SRR1180958

SRR1180959

SRR1180963

SRR1180964

SRR1180969

SRR1180970

SRR1180971

SRR1180975

SRR1180976

SRR1180983

SRR1180984

SRR1180989

SRR1180990

SRR1180991

SRR1180994

SRR1180995

SRR1180997

SRR1181002

SRR1181003

SRR1181007

SRR1181008

SRR1181013

SRR1181014

SRR1181018

SRR1181019

SRR1181024

SRR1181025

SRR1181026

SRR1181031

SRR1181032

SRR1181033

SRR1181034

SRR1181035

SRR1181036

SRR1181041

SRR1181042

SRR1181045

SRR1181046

SRR1181047

SRR1181048

SRR1181060

SRR1181061

SRR1181062

SRR1181069

SRR1181071

SRR1181072

SRR1181073

SRR1181074

SRR1181075

SRR1181076

SRR1181080

SRR1181081

SRR1181082

SRR1181083

SRR1181084

SRR1181086

SRR1181087

SRR1181088

SRR1181089

SRR1181093

SRR1181094

SRR1181095

SRR1181096

SRR1181097

SRR1181098

SRR1181099

SRR1181102

SRR1181103

SRR1181104

SRR1181105

SRR1181107

SRR1181108

SRR1181109

SRR1181110

SRR1181111

SRR1181114

SRR1181115

SRR1181116

SRR1181117

SRR1181118

SRR1181119

SRR1181122

SRR1181123

SRR1181124

SRR1181125

SRR1181126

SRR1181128

SRR1181129

SRR1181130

SRR1181131

SRR1181132

SRR1181133

SRR1181134

SRR1181135

SRR1181137

SRR1181138

SRR1181141

SRR1181142

SRR1181145

SRR1181149

SRR1181150

SRR1181151

SRR1181152

SRR1181153

SRR1181154

SRR1181155

SRR1181157

SRR1181158

SRR1181159

SRR1181160

SRR1181162

SRR1181163

SRR1181164

SRR1181165

SRR1181166

SRR1181167

SRR1181168

SRR1181169

SRR1181170

SRR1181171

SRR1181172

SRR1181173

SRR1181174

SRR1181196

SRR1181197

SRR1181198

SRR1181202

SRR1181203

SRR1181204

SRR1181205

SRR1181206

SRR1181207

SRR1181208

SRR1181209

SRR1181210

SRR1181212

SRR1181213

SRR1181216

SRR1181217

SRR1181218

SRR1181219

SRR1181220

SRR1181238

SRR1181241

SRR1181243

SRR1181247

SRR1181248

SRR1181249

SRR1181253

SRR1181254

SRR1181255

SRR1181257

SRR1181259

SRR1181260

SRR1181262

SRR1181278

SRR1181282

SRR1181284

SRR1181288

SRR1181290

SRR1181294

SRR1181300

SRR1181301

SRR1181304

SRR1181305

SRR1181306

SRR1181307

SRR1181308

SRR1181309

SRR1181310

SRR1181311

SRR1181312

SRR1181313

SRR1181315

SRR1181316

SRR1181318

SRR1181320

SRR1181321

SRR1181322

SRR1181324

SRR1181326

SRR1181327

SRR1181329

SRR1181330

SRR1181331

SRR1181350

SRR1181352

SRR1181353

SRR1181354

SRR1181355

SRR1181357

SRR1181367

SRR1181370

SRR1181375

SRR1181376

SRR1181384

SRR1181385

SRR1181391

SRR1181395

SRR1181396

SRR1181401

SRR1181404

SRR1181411

SRR1182970

SRR1182972

SRR1182981

SRR1182986

SRR1182987

SRR1183000

SRR1183001

SRR1183008

SRR1183012

SRR1183017

SRR1183028

SRR1183033

SRR1183035

SRR1183041

SRR1183042

SRR1183043

SRR1183048

SRR1183052

SRR1183053

SRR1183058

SRR1183066

SRR1183073

SRR1183074

SRR1183081

SRR1183082

SRR1183085

SRR1183087

SRR1183088

SRR1183089

SRR1183090

SRR1183095

SRR1183101

SRR1183110

SRR1183118

SRR1183119

SRR1183120

SRR1183124

SRR1183125

SRR1183131

SRR1183136

SRR1183143

SRR1183146

SRR1183171

SRR1183663

SRR1183959

SRR1183960

SRR1183961

SRR1183962

SRR1183963

SRR1183966

SRR1183967

SRR1183968

SRR1183969

SRR1184001

SRR1184002

SRR1184003

SRR1184004

SRR1184012

SRR1184014

SRR1184015

SRR1184018

SRR1184019

SRR1184020

SRR1184022

SRR1184026

SRR1184027

SRR1184034

SRR1184035

SRR1184036

SRR1184037

SRR1184038

SRR1184039

SRR1184040

SRR1184041

SRR1184113

SRR1184115

SRR1184118

SRR1184120

SRR1184121

SRR1184123

SRR1184125

SRR1184127

SRR1184292

SRR1184293

SRR1184295

SRR1184296

SRR1184297

SRR1184299

SRR1184300

SRR1184301

SRR1184303

SRR1184304

SRR1184305

SRR1184306

SRR1184307

SRR1184309

SRR1184310

SRR1184311

SRR1184313

SRR1184314

SRR1184315

SRR1184316

SRR1184317

SRR1184318

SRR1184319

SRR1184320

SRR1184321

SRR1184322

SRR1184323

SRR1184324

SRR1184325

SRR1184326

SRR1184327

SRR1184328

SRR1184330

SRR1184331

SRR1184332

SRR1184333

SRR1184334

SRR1184335

SRR1184336

SRR1184337

SRR1184338

SRR1184339

SRR1184340

SRR1184341

SRR1184342

SRR1184343

SRR1184344

SRR1184345

SRR1184347

SRR1184348

SRR1184351

SRR1184352

SRR1184353

SRR1184354

SRR1184355

SRR1184356

SRR1184357

SRR1184359

SRR1184360

SRR1184361

SRR1184362

SRR1184363

SRR1184364

SRR1184365

SRR1184366

SRR1184367

SRR1184368

SRR1184369

SRR1184370

SRR1184371

SRR1184373

SRR1184374

SRR1184375

SRR1184376

SRR1184377

SRR1184378

SRR1184382

SRR1184384

SRR1184385

SRR1184386

SRR1184387

SRR1184389

SRR1184394

SRR1196466

SRR1196467

SRR1196472

SRR1196473

SRR1196475

SRR1196477

SRR1196479

SRR1196480

SRR1196484

SRR1196485

SRR1196486

SRR1196487

SRR1196491

SRR1196492

SRR1196493

SRR1196496

SRR1196497

SRR1196498

SRR1196499

SRR1196503

SRR1196504

SRR1196505

SRR1196506

SRR1196510

SRR1196511

SRR1196512

SRR1196513

SRR1196515

SRR1196516

SRR1196517

SRR1196518

SRR1196522

SRR1196523

SRR1196524

SRR1196525

SRR1196527

SRR1196528

SRR1196529

SRR1196530

SRR1196533

SRR1196534

SRR1196535

SRR1196536

SRR1196539

SRR1196540

SRR1196541

SRR1196542

SRR1196544

SRR1196547

SRR1196551

SRR1196552

SRR1196553

SRR1196555

SRR1196556

SRR1196558

SRR1196563

SRR1196564

SRR1196565

SRR1196568

SRR1196569

SRR1196570

SRR1196572

SRR1196575

SRR1196576

SRR1196577

SRR1196580

SRR1196581

SRR1196586

SRR1196587

SRR1196588

SRR1196670

SRR1196671

SRR1196678

SRR1196685

SRR1196686

SRR1196702

SRR1196716

SRR1196723

SRR1196724

SRR1196740

SRR1196754

SRR1196769

SRR1196785

SRR1203930

SRR1206024

SRR2099924

SRR2099925

SRR2099926

SRR2099927

SRR2099928

SRR2099929

SRR2099931

SRR2099932

SRR2099934

SRR2099935

SRR2099937

SRR2099938

SRR2099939

SRR2099940

SRR2099941

SRR2099942

SRR2099943

SRR2099944

SRR2099945

SRR2099946

SRR2099947

SRR2099949

SRR2099950

SRR2099951

SRR2099955

SRR2099956

SRR2099957

SRR2099958

SRR2099959

SRR2099960

SRR2099961

SRR2099962

SRR2099964

SRR2099965

SRR2099966

SRR2099967

SRR2099968

SRR2099969

SRR2099970

SRR2099972

SRR2099974

SRR2099975

SRR2099976

SRR2099977

SRR2099979

SRR2099980

SRR2099981

SRR2099982

SRR2099983

SRR2099984

SRR2099985

SRR2099986

SRR2099988

SRR2099989

SRR2099990

SRR2099991

SRR2099992

SRR2099993

SRR2099994

SRR2099995

SRR2099996

SRR2099997

SRR2099998

SRR2099999

SRR2100000

SRR2100001

SRR2100002

SRR2100003

SRR2100004

SRR2100005

SRR2100006

SRR2100007

SRR2100008

SRR2100009

SRR2100010

SRR2100011

SRR2100012

SRR2100013

SRR2100014

SRR2100015

SRR2100016

SRR2100017

SRR2100018

SRR2100019

SRR2100020

SRR2100022

SRR2100023

SRR2100024

SRR2100025

SRR2100027

SRR2100028

SRR2100029

SRR2100030

SRR2100031

SRR2100032

SRR2100033

SRR2100034

SRR2100035

SRR2100036

SRR2100037

SRR2100038

SRR2100039

SRR2100040

SRR2100041

SRR2100042

SRR2100045

SRR2100046

SRR2100047

SRR2100048

SRR2100049

SRR2100050

SRR2100051

SRR2100052

SRR2100053

SRR2100054

SRR2100055

SRR2100056

SRR2100057

SRR2100058

SRR2100059

SRR2100061

SRR2100062

SRR2100063

SRR2100064

SRR2100065

SRR2100066

SRR2100069

SRR2100070

SRR2100071

SRR2100073

SRR2100074

SRR2100075

SRR2100076

SRR2100078

SRR2100079

SRR2100080

SRR2100081

SRR2100082

SRR2100083

SRR2100084

SRR2100085

SRR2100086

SRR2100087

SRR2100088

SRR2100089

SRR2100091

SRR2100092

SRR2100093

SRR2100095

SRR2100096

SRR2100097

SRR2100098

SRR2100099

SRR2100100

SRR2100101

SRR2100102

SRR2100103

SRR2100104

SRR2100105

SRR2100106

SRR2100107

SRR2100108

SRR2100109

SRR2100110

SRR2100111

SRR2100112

SRR2100113

SRR2100114

SRR2100115

SRR2100116

SRR2100117

SRR2100118

SRR2100119

SRR2100121

SRR2100122

SRR2100123

SRR2100124

SRR2100125

SRR2100126

SRR2100127

SRR2100128

SRR2100129

SRR2100130

SRR2100131

SRR2100132

SRR2100133

SRR2100134

SRR2100135

SRR2100136

SRR2100137

SRR2100138

SRR2100139

SRR2100140

SRR2100141

SRR2100142

SRR2100143

SRR2100144

SRR2100146

SRR2100147

SRR2100148

SRR2100149

SRR2100150

SRR2100151

SRR2100152

SRR2100153

SRR2100154

SRR2100155

SRR2100156

SRR2100157

SRR2100158

SRR2100159

SRR2100160

SRR2100161

SRR2100162

SRR2100164

SRR2100165

SRR2100166

SRR2100167

SRR2100168

SRR2100169

SRR2100170

SRR2100171

SRR2100172

SRR2100173

SRR2100174

SRR2100175

SRR2100176

SRR2100177

SRR2100178

SRR2100179

SRR2100180

SRR2100181

SRR2100182

SRR2100183

SRR2100184

SRR2100185

SRR2100186

SRR2100187

SRR2100188

SRR2100189

SRR2100191

SRR2100192

SRR2100193

SRR2100194

SRR2100195

SRR2100196

SRR2100197

SRR2100198

SRR2100199

SRR2100200

SRR2100201

SRR2100202

SRR2100203

SRR2100204

SRR2100205

SRR2100206

SRR2100208

SRR2100209

SRR2100210

SRR2100211

SRR2100212

SRR2100213

SRR2100214

SRR2100215

SRR2100216

SRR2100217

SRR2100218

SRR2100219

SRR2100220

SRR2100221

SRR2100222

SRR2100223

SRR2100224

SRR2100225

SRR2100226

SRR2100227

SRR2100228

SRR2100229

SRR2100230

SRR2100231

SRR2100232

SRR2100233

SRR2100234

SRR2100235

SRR2100236

SRR2100237

SRR2100238

SRR2100239

SRR2100240

SRR2100241

SRR2100242

SRR2100243

SRR2100244

SRR2100245

SRR2100246

SRR2100247

SRR2100248

SRR2100249

SRR2100250

SRR2100251

SRR2100252

SRR2100253

SRR2100254

SRR2100255

SRR2100256

SRR2100257

SRR2100258

SRR2100259

SRR2100260

SRR2100261

SRR2100262

SRR2100263

SRR2100264

SRR2100265

SRR2100266

SRR2100267

SRR2100268

SRR2100269

SRR2100270

SRR2100272

SRR2100273

SRR2100274

SRR2100275

SRR2100276

SRR2100277

SRR2100278

SRR2100279

SRR2100280

SRR2100281

SRR2100282

SRR2100283

SRR2100284

SRR2100285

SRR2100286

SRR2100287

SRR2100289

SRR2100290

SRR2100291

SRR2100292

SRR2100293

SRR2100294

SRR2100296

SRR2100297

SRR2100298

SRR2100299

SRR2100300

SRR2100301

SRR2100302

SRR2100303

SRR2100304

SRR2100305

SRR2100306

SRR2100307

SRR2100308

SRR2100309

SRR2100310

SRR2100311

SRR2100313

SRR2100314

SRR2100315

SRR2100316

SRR2100317

SRR2100318

SRR2100319

SRR2100320

SRR2100321

SRR2100323

SRR2100326

SRR2100328

SRR2100330

SRR2100331

SRR2100332

SRR2100333

SRR2100334

SRR2100335

SRR2100336

SRR2100337

SRR2100338

SRR2100339

SRR2100340

SRR2100341

SRR2100342

SRR2100343

SRR2100344

SRR2100345

SRR2100346

SRR2100347

SRR2100349

SRR2100350

SRR2100351

SRR2100352

SRR2100353

SRR2100354

SRR2100356

SRR2100357

SRR2100359

SRR2100360

SRR2100361

SRR2100362

SRR2100363

SRR2100364

SRR2100365

SRR2100366

SRR2100367

SRR2100368

SRR2100369

SRR2100370

SRR2100371

SRR2100372

SRR2100373

SRR2100374

SRR2100375

SRR2100376

SRR2100377

SRR2100378

SRR2100379

SRR2100380

SRR2100381

SRR2100382

SRR2100383

SRR2100384

SRR2100385

SRR2100386

SRR2100387

SRR2100388

SRR2100389

SRR2100390

SRR2100391

SRR2100392

SRR2100393

SRR2100394

SRR2100395

SRR2100396

SRR2100397

SRR2100399

SRR2100401

SRR2100402

SRR2100403

SRR2100404

SRR2100405

SRR2100406

SRR2100408

SRR2100409

SRR2100410

SRR2100411

SRR2100412

SRR2100414

SRR2100415

SRR2100418

SRR2100419

SRR2100420

SRR2100422

SRR2100423

SRR2100424

SRR2100425

SRR2100426

SRR2100427

SRR2100429

SRR2100430

SRR2100431

SRR2100432

SRR2100433

SRR2100436

SRR2100437

SRR2100439

SRR2100441

SRR2100442

SRR2100443

SRR2100444

SRR2100445

SRR2100446

SRR2100447

SRR2100448

SRR2100449

SRR2100450

SRR2100451

SRR2100452

SRR2100453

SRR2100454

SRR2100456

SRR2100457

SRR2100458

SRR2100459

SRR2100460

SRR2100461

SRR2100462

SRR2100463

SRR2100464

SRR2100465

SRR2100466

SRR2100467

SRR2100468

SRR2100469

SRR2100470

SRR2100471

SRR2100472

SRR2100473

SRR2100474

SRR2100475

SRR2100476

SRR2100477

SRR2100478

SRR2100479

SRR2100480

SRR2100481

SRR2100482

SRR2100483

SRR2100484

SRR2100485

SRR2100486

SRR2100487

SRR2100488

SRR2100489

SRR2100490

SRR2100491

SRR2100492

SRR2100493

SRR2100494

SRR2100495

SRR2100496

SRR2100497

SRR2100498

SRR2100499

SRR2100500

SRR2100501

SRR2100502

SRR2100503

SRR2100504

SRR2100505

SRR2100506

SRR2100507

SRR2100508

SRR2100509

SRR2100510

SRR2100511

SRR2100512

SRR2100513

SRR2100514

SRR2100515

SRR2100516

SRR2100517

SRR2100518

SRR2100519

SRR2100520

SRR2100521

SRR2100522

SRR2100523

SRR2100524

SRR2100525

SRR2100526

SRR2100527

SRR2100528

SRR2100529

SRR2100530

SRR2100531

SRR2100532

SRR2100533

SRR2100534

SRR2100535

SRR2100536

SRR2100537

SRR2100538

SRR2100539

SRR2100540

SRR2100541

SRR2100542

SRR2100543

SRR2100544

SRR2100545

SRR2100546

SRR2100547

SRR2100548

SRR2100549

SRR2100550

SRR2100551

SRR2100552

SRR2100553

SRR2100554

SRR2100555

SRR2100556

SRR2100557

SRR2100558

SRR2100559

SRR2100560

SRR2100561

SRR2100562

SRR2100563

SRR2100564

SRR2100565

SRR2100566

SRR2100567

SRR2100568

SRR2100569

SRR2100570

SRR2100571

SRR2100572

SRR2100573

SRR2100574

SRR2100575

SRR2100576

SRR2100577

SRR2100578

SRR2100579

SRR2100580

SRR2100581

SRR2100582

SRR2100583

SRR2100584

SRR2100585

SRR2100586

SRR2100587

SRR2100588

SRR2100589

SRR2100590

SRR2100591

SRR2100592

SRR2100593

SRR2100594

SRR2100595

SRR2100596

SRR2100597

SRR2100598

SRR2100599

SRR2100600

SRR2100601

SRR2100602

SRR2100603

SRR2100604

SRR2100605

SRR2100606

SRR2100607

SRR2100608

SRR2100609

SRR2100610

SRR2100611

SRR2100613

SRR2100614

SRR2100615

SRR2100616

SRR2100617

SRR2100618

SRR2100619

SRR2100620

SRR2100621

SRR2100622

SRR2100623

SRR2100624

SRR2100625

SRR2100626

SRR2100627

SRR2100628

SRR2100629

SRR2100630

SRR2100631

SRR2100632

SRR2100633

SRR2100634

SRR2100635

SRR2100636

SRR2100637

SRR2100638

SRR2100639

SRR2100640

SRR2100641

SRR2100642

SRR2100643

SRR2100644

SRR2100645

SRR2100646

SRR2100647

SRR2100648

SRR2100649

SRR2100650

SRR2100651

SRR2100652

SRR2100653

SRR2100654

SRR2100655

SRR2100656

SRR2100657

SRR2100658

SRR2100659

SRR2100660

SRR2100661

SRR2100662

SRR2100663

SRR2100664

SRR2100665

SRR2100666

SRR2100667

SRR2100668

SRR2100669

SRR2100670

SRR2100671

SRR2100672

SRR2100673

SRR2100674

SRR2100675

SRR2100676

SRR2100677

SRR2100678

SRR2100679

SRR2100680

SRR2100681

SRR2100682

SRR2100683

SRR2100684

SRR2100685

SRR2100686

SRR2100687

SRR2100688

SRR2100689

SRR2100690

SRR2100691

SRR2100692

SRR2100693

SRR2100694

SRR2100695

SRR2100696

SRR2100697

SRR2100698

SRR2100699

SRR2100700

SRR2100701

SRR2100702

SRR2100703

SRR2100704

SRR2100705

SRR2100706

SRR2100707

SRR2100708

SRR2100709

SRR2100710

SRR2100711

SRR2100712

SRR2100713

SRR2100714

SRR2100715

SRR2100716

SRR2100717

SRR2100718

SRR2100719

SRR2100720

SRR2100721

SRR2100722

SRR2100723

SRR2100724

SRR2100725

SRR2100726

SRR2100727

SRR2100728

SRR2100729

SRR2100730

SRR2100731

SRR2100732

SRR2100733

SRR2100734

SRR2100735

SRR2100736

SRR2100737

SRR2100738

SRR2100739

SRR2100741

SRR2100743

SRR2100744

SRR2100745

SRR2100746

SRR2100747

SRR2100748

SRR2100749

SRR2100750

SRR2100751

SRR2100752

SRR2100753

SRR2100754

SRR2100756

SRR2100757

SRR2100758

SRR2100759

SRR2100760

SRR2100761

SRR2100762

SRR2100763

SRR2100765

SRR2100766

SRR2100767

SRR2100768

SRR2100769

SRR2100770

SRR2100772

SRR2100773

SRR2100774

SRR2100775

SRR2100776

SRR2100777

SRR2100778

SRR2100779

SRR2100780

SRR2100781

SRR2100782

SRR2100783

SRR2100784

SRR2100785

SRR2100786

SRR2100787

SRR2100788

SRR2100789

SRR2100790

SRR2100791

SRR2100792

SRR2100793

SRR2100794

SRR2100795

SRR2100796

SRR2100798

SRR2100799

SRR2100800

SRR2100802

SRR2100803

SRR2100804

SRR2100805

SRR2100806

SRR2100807

SRR2100808

SRR2100809

SRR2100810

SRR2100812

SRR2100813

SRR2100814

SRR2100815

SRR2100816

SRR2100817

SRR2100818

SRR2100819

SRR2100820

SRR2100821

SRR2100822

SRR2100823

SRR2100824

SRR2100825

SRR2100829

SRR2100831

SRR2100832

SRR2100833

SRR2100834

SRR2100835

SRR2100836

SRR2100837

SRR2100838

SRR2100839

SRR2100840

SRR2100841

SRR2100842

SRR2100843

SRR2100844

SRR2100847

SRR2100848

SRR2100850

SRR2100852

SRR2100853

SRR2100854

SRR2100855

SRR2100856

SRR2100857

SRR2100858

SRR2100859

SRR2100860

SRR2100861

SRR2100862

SRR2100863

SRR2100864

SRR2100865

SRR2100866

SRR2100867

SRR2100868

SRR2100869

SRR2100870

SRR2100871

SRR2100873

SRR2100874

SRR2100875

SRR2100877

SRR2100878

SRR2100879

SRR2100881

SRR2100882

SRR2100883

SRR2100884

SRR2100885

SRR2100886

SRR2100887

SRR2100888

SRR2100889

SRR2100890

SRR2100891

SRR2100893

SRR2100894

SRR2100895

SRR2100899

SRR2100900

SRR2100901

SRR2100902

SRR2100903

SRR2100904

SRR2100905

SRR2100906

SRR2100907

SRR2100908

SRR2100909

SRR2100910

SRR2100911

SRR2100912

SRR2100913

SRR2100914

SRR2100915

SRR2100916

SRR2100917

SRR2100919

SRR2100920

SRR2100921

SRR2100922

SRR2100923

SRR2100924

SRR2100925

SRR2100926

SRR2100927

SRR2100928

SRR2100929

SRR2100930

SRR2100931

SRR2100932

SRR2100933

SRR2100934

SRR2100935

SRR2100936

SRR2100937

SRR2100938

SRR2100939

SRR2100940

SRR2100941

SRR2100942

SRR2100943

SRR2100944

SRR2100945

SRR2100946

SRR2100949

SRR2100950

SRR2100951

SRR2100952

SRR2100953

SRR2100954

SRR2100955

SRR2100956

SRR2100957

SRR2100958

SRR2100959

SRR2100960

SRR2100961

SRR2100962

SRR2100963

SRR2100964

SRR2100965

SRR2100966

SRR2100967

SRR2100968

SRR2100969

SRR2100970

SRR2100971

SRR2100972

SRR2100973

SRR2100974

SRR2100975

SRR2100976

SRR2100977

SRR2100978

SRR2100979

SRR2100980

SRR2100981

SRR2100982

SRR2100983

SRR2100984

SRR2100985

SRR2100986

SRR2100987

SRR2100988

SRR2100989

SRR2100990

SRR2100991

SRR2100992

SRR2100993

SRR2100994

SRR2100995

SRR2100996

SRR2100997

SRR2100998

SRR2100999

SRR2101000

SRR2101001

SRR2101002

SRR2101003

SRR2101004

SRR2101005

SRR2101006

SRR2101007

SRR2101008

SRR2101009

SRR2101010

SRR2101011

SRR2101012

SRR2101014

SRR2101015

SRR2101016

SRR2101017

SRR2101018

SRR2101019

SRR2101020

SRR2101021

SRR2101022

SRR2101023

SRR2101024

SRR2101025

SRR2101026

SRR2101027

SRR2101028

SRR2101029

SRR2101030

SRR2101031

SRR2101032

SRR2101033

SRR2101034

SRR2101035

SRR2101036

SRR2101037

SRR2101038

SRR2101039

SRR2101040

SRR2101041

SRR2101042

SRR2101044

SRR2101045

SRR2101046

SRR2101047

SRR2101048

SRR2101050

SRR2101051

SRR2101053

SRR2101054

SRR2101055

SRR2101056

SRR2101057

SRR2101058

SRR2101059

SRR2101060

SRR2101061

SRR2101062

SRR2101063

SRR2101064

SRR2101065

SRR2101066

SRR2101067

SRR2101068

SRR2101070

SRR2101071

SRR2101072

SRR2101073

SRR2101074

SRR2101075

SRR2101076

SRR2101077

SRR2101078

SRR2101079

SRR2101080

SRR2101081

SRR2101082

SRR2101083

SRR2101084

SRR2101085

SRR2101086

SRR2101088

SRR2101089

SRR2101097

SRR2101098

SRR2101099

SRR2101113

SRR2101116

SRR2101117

SRR2101118

SRR2101119

SRR2101122

SRR2101123

SRR2101124

SRR2101125

SRR2101130

SRR2101131

SRR2101134

SRR2101140

SRR2101141

SRR2101143

SRR2101154

SRR2101159

SRR2101175

SRR2101179

SRR2101193

SRR2101205

SRR2101207

SRR2101211

SRR2101212

SRR2101226

SRR2101244

SRR2101249

SRR2101250

SRR2101251

SRR2101254

SRR2101256

SRR2101257

SRR2101258

SRR2101259

SRR2101260

SRR2101261

SRR2101262

SRR2101263

SRR2101264

SRR2101265

SRR2101266

SRR2101267

SRR2101268

SRR2101269

SRR2101270

SRR2101271

SRR2101272

SRR2101273

SRR2101274

SRR2101275

SRR2101276

SRR2101277

SRR2101278

SRR2101279

SRR2101280

SRR2101281

SRR2101282

SRR2101283

SRR2101284

SRR2101285

SRR2101286

SRR2101288

SRR2101289

SRR2101290

SRR2101291

SRR2101292

SRR2101293

SRR2101294

SRR2101295

SRR2101296

SRR2101297

SRR2101298

SRR2101299

SRR2101300

SRR2101301

SRR2101302

SRR2101303

SRR2101304

SRR2101306

SRR2101307

SRR2101308

SRR2101309

SRR2101310

SRR2101313

SRR2101314

SRR2101315

SRR2101316

SRR2101317

SRR2101319

SRR2101320

SRR2101321

SRR2101322

SRR2101323

SRR2101324

SRR2101325

SRR2101326

SRR2101327

SRR2101328

SRR2101329

SRR2101330

SRR2101331

SRR2101332

SRR2101333

SRR2101334

SRR2101336

SRR2101337

SRR2101338

SRR2101339

SRR2101340

SRR2101341

SRR2101342

SRR2101343

SRR2101345

SRR2101346

SRR2101347

SRR2101348

SRR2101349

SRR2101350

SRR2101351

SRR2101352

SRR2101355

SRR2101356

SRR2101357

SRR2101358

SRR2101359

SRR2101361

SRR2101362

SRR2101363

SRR2101366

SRR2101368

SRR2101370

SRR2101371

SRR2101373

SRR2101374

SRR2101375

SRR2101376

SRR2101377

SRR2101378

SRR2101379

SRR2101382

SRR2101383

SRR2101385

SRR2101386

SRR2101389

SRR2101390

SRR2101394

SRR2101397

SRR2101399

SRR2101401

SRR2101403

SRR2101406

SRR2101407

SRR2101410

SRR2101411

SRR2101412

SRR2101413

SRR2101416

SRR2101418

SRR2101419

SRR2101420

SRR2101422

SRR2101424

SRR2101426

SRR2101427

SRR2101428

SRR2101429

SRR2101430

SRR2101431

SRR2101432

SRR2101434

SRR2101439

SRR2101441

SRR2101444

SRR2101445

SRR2101446

SRR2101447

SRR2101449

SRR2101452

SRR2101456

SRR2101458

SRR2101461

SRR2101463

SRR2101464

SRR2101466

SRR2101467

SRR2101468

SRR2101469

SRR2101470

SRR2101472

SRR2101473

SRR2101474

SRR2101475

SRR2101477

SRR2101479

SRR2101481

SRR2101483

SRR2101484

SRR2101485

SRR2101488

SRR2101489

SRR2101493

SRR2101495

SRR2101496

SRR2101497

SRR2101499

SRR2101500

SRR2101503

SRR2101504

SRR2101507

SRR2101508

SRR2101509

SRR2101514

SRR2101515

SRR2101516

SRR2101519

SRR2101522

SRR2101523

SRR2101526

SRR2101527

SRR2101528

SRR2101530

SRR2101533

SRR2101536

SRR2101537

SRR2101538

SRR2101541

SRR2101542

SRR2101543

SRR2101545

SRR2101547

SRR2101548

SRR2101549

SRR2101553

SRR2101554

SRR2101556

SRR2101557

SRR2101558

SRR2101559

SRR2101560

SRR2101562

SRR2101563

SRR2101564

SRR2101566

SRR2101567

SRR2101568

SRR2101570

SRR2101574

SRR2101575

SRR2101576

SRR2101578

SRR2101579

SRR2101580

SRR2101581

SRR2101583

SRR2101584

SRR2101585

SRR2101587

SRR2101588

SRR2101591

SRR2101592

SRR2101593

SRR2101594

SRR2101596

SRR2101597

SRR2101598

SRR2101599

SRR2101602

SRR2101606

SRR2101607

SRR2101608

SRR2101609

SRR2101610

SRR2101611

SRR2101612

SRR2101613

SRR2101614

SRR2101615

SRR2101616

SRR2101618

SRR2101619

SRR2101621

SRR2101622

SRR2101623

SRR2101624

SRR2101629

SRR2101630

SRR2101631

SRR2101633

SRR2101634

SRR2101635

SRR2101638

SRR2101640

SRR2101641

SRR2101642

SRR2101644

SRR2101645

SRR2101646

SRR2101648

SRR2101652

SRR2101654

SRR2101655

SRR2101657

SRR2101660

SRR2101664

SRR2101667

SRR2101670

SRR2101672

SRR2101673

SRR2101674

SRR2101675

SRR2101676

SRR2101679

SRR2101680

SRR2101682

SRR2101688

SRR2101690

SRR2101692

SRR2101694

SRR2101696

SRR2101699

SRR2101700

SRR2101701

SRR2101704

SRR2101706

SRR2101707

SRR2101708

SRR2101710

SRR2101714

SRR2101715

SRR2101716

SRR2101717

SRR2101718

SRR2101719

SRR2101720

SRR2101722

SRR2101723

SRR2101724

SRR2101725

SRR2101727

SRR2101729

SRR2101731

SRR2101733

SRR2101734

SRR2101735

SRR2101736

SRR2101737

SRR2101738

SRR2101743

SRR2101744

SRR2101745

SRR2101746

SRR2101747

SRR2101748

SRR2101749

SRR2101750

SRR2101751

SRR2101752

SRR2101753

SRR2101754

SRR2101755

SRR2101757

SRR2101760

SRR2101761

SRR2101762

SRR2101763

SRR2101765

SRR2101766

SRR2101768

SRR2101771

SRR2101772

SRR2101775

SRR2101776

SRR2101777

SRR2101780

SRR2101781

SRR2101782

SRR2101784

SRR2101785

SRR2101788

SRR2101790

SRR2101792

SRR2101794

SRR2101797

SRR2101798

SRR2101799

SRR2101800

SRR2101801

SRR2101805

SRR2101807

SRR2101808

SRR2101809

SRR2101811

SRR2101813

SRR2101815

SRR2101817

SRR2101818

SRR2101819

SRR2101820

SRR2101821

SRR2101822

SRR2101823

SRR2101824

SRR2101826

SRR2101828

SRR671719

SRR671720

SRR671721

SRR671722

SRR671723

SRR671724

SRR671725

SRR671726

SRR671727

SRR671728

SRR671729

SRR671730

SRR671731

SRR671732

SRR671733

SRR671734

SRR671735

SRR671736

SRR671737

SRR671738

SRR671739

SRR671740

SRR671741

SRR671742

SRR671743

SRR671744

SRR671745

SRR671746

SRR671747

SRR671748

SRR671749

SRR671750

SRR671751

SRR671752

SRR671753

SRR671754

SRR671755

SRR671756

SRR671757

SRR671758

SRR671759

SRR671760

SRR671761

SRR671762

SRR671763

SRR671764

SRR671765

SRR671766

SRR671767

SRR671768

SRR671769

SRR671770

SRR671771

SRR671772

SRR671773

SRR671774

SRR671776

SRR671777

SRR671779

SRR671780

SRR671781

SRR671782

SRR671783

SRR671784

SRR671785

SRR671786

SRR671787

SRR671788

SRR671789

SRR671790

SRR671791

SRR671792

SRR671793

SRR671794

SRR671795

SRR671796

SRR671797

SRR671798

SRR671799

SRR671800

SRR671801

SRR671802

SRR671803

SRR671804

SRR671805

SRR671806

SRR671807

SRR671808

SRR671809

SRR671810

SRR671811

SRR671812

SRR671813

SRR671814

SRR671815

SRR671816

SRR671817

SRR671818

SRR671819

SRR671820

SRR671821

SRR671822

SRR671823

SRR671824

SRR671825

SRR671826

SRR671827

SRR671828

SRR671829

SRR671830

SRR671831

SRR671832

SRR671833

SRR671834

SRR671835

SRR671836

SRR671837

SRR671838

SRR671839

SRR671840

SRR671841

SRR671842

SRR671843

SRR671844

SRR671845

SRR671846

SRR671847

SRR671848

SRR671849

SRR671850

SRR671851

SRR671852

SRR671853

SRR671854

SRR671855

SRR671856

SRR671857

SRR671858

SRR671859

SRR671860

SRR671861

SRR671862

SRR671863

SRR671864

SRR671865

SRR671866

SRR671867

SRR671868

SRR671869

SRR671870

SRR671871

SRR671872

SRR671873

SRR671874

SRR671875

SRR671876

SRR671877

SRR671878

SRR671879

SRR832977

SRR832978

SRR832982

SRR832984

SRR832986

SRR832988

SRR832991

SRR832992

SRR832994

SRR832995

SRR832997

SRR832998

SRR832999

SRR833000

SRR833001

SRR833003

SRR833007

SRR833008

SRR833010

SRR833011

SRR833013

SRR833015

SRR833016

SRR833017

SRR833018

SRR833019

SRR833020

SRR833021

SRR833023

SRR833024

SRR833026

SRR833029

SRR833031

SRR833033

SRR833034

SRR833037

SRR833039

SRR833040

SRR833043

SRR833044

SRR833046

SRR833047

SRR833048

SRR833053

SRR833055

SRR833056

SRR833057

SRR833058

SRR833059

SRR833062

SRR833063

SRR833064

SRR833065

SRR833067

SRR833068

SRR833071

SRR833073

SRR833074

SRR833075

SRR833077

SRR833078

SRR833079

SRR833080

SRR833081

SRR833085

SRR833086

SRR833087

SRR833091

SRR833092

SRR833094

SRR833095

SRR833097

SRR833099

SRR833100

SRR833105

SRR833106

SRR833110

SRR833111

SRR833112

SRR833113

SRR833115

SRR833116

SRR833117

SRR833119

SRR833120

SRR833122

SRR833124

SRR833126

SRR833127

SRR833129

SRR833130

SRR833131

SRR833133

SRR833134

SRR833136

SRR833137

SRR833140

SRR833141

SRR833142

SRR833143

SRR833144

SRR833145

SRR833146

SRR833147

SRR833148

SRR833149

SRR833151

SRR833154

SRR833157

SRR833162

SRR833163

SRR833164

SRR833167

SRR833168

SRR833169

SRR833170

SRR833171

SRR833172

SRR833173

SRR833174

SRR833175

SRR833176

SRR833177

SRR833178

SRR833179

SRR833184

SRR833185

SRR833186

SRR833188

SRR833190

SRR833191

SRR833192

SRR833193

SRR833194

SRR833195

SRR833196

SRR847775

SRR847776

SRR847777

SRR847778

SRR847779

SRR847780

SRR847781

SRR847782

SRR847783

SRR847784

SRR847785

SRR847787

SRR847788

SRR847789

SRR847790

SRR847791

SRR847792

SRR847793

SRR847794

SRR847795

SRR847796

SRR847797

SRR847798

SRR847799

SRR847800

SRR847803

SRR924205

SRR924206

SRR924207

SRR924209

SRR924210

SRR924211

SRR924212

SRR924213

SRR924214

SRR924216

SRR924217

SRR924218

SRR924219

SRR924220

SRR924221

SRR924222

SRR924223

SRR924224

SRR924225

SRR924226

SRR924227

SRR924228

SRR924230

SRR924231

SRR924233

SRR924234

SRR924237

SRR924692

SRR924693

SRR924694

SRR924697

SRR924698

SRR924699

SRR924700

SRR924701

SRR924703

SRR924704

SRR924705

SRR924706

SRR924707

SRR924708

SRR924710

SRR924711

SRR924712

SRR924713

SRR924714

SRR924715

SRR924716

SRR924718

SRR924719

SRR958198

SRR958201

SRR958204

SRR958207

SRR958210

SRR958216

SRR958221

SRR958224

SRR958225

SRR958228

SRR958231

SRR958237

SRR958480

SRR958483

SRR958488

SRR958489

SRR958492

SRR958495

SRR958501

SRR958504

SRR998576

SRR998579

SRR998580

SRR998583

SRR998588

SRR998591

SRR998599

SRR998607

SRR998608

SRR998616

SRR998620

SRR998632

SRR998640

SRR998647

SRR998652

SRR998667

SRR998671

SRR998672

SRR998683

SRR998703

SRR998707

SRR998715

SRR998716

SRR998720

SRR998724

SRR998731

SRR998736

SRR998748

SRR998752

SRR998756

SRR998760

SRR998763

SRR998767

SRR998768

SRR998787

SRR998791

SRR998792

SRR998796

SRR998807

SRR998809

SRR998812

SRR998816

SRR998821

SRR998829

SRR998836

SRR998837

SRR998840

TDR103S116L002

TDR105S128L002

TDR107S140L002

TDR10S168L002

TDR117S151L002

TDR118S163L002

TDR11S87L002

TDR121S175L002

TDR124S94L002

TDR125S105L002

TDR127S117L002

TDR128S129L002

TDR129S132L002

TDR12S99L002

TDR135S141L002

TDR139S152L002

TDR13S110L002

TDR145S164L002

TDR147S176L002

TDR14S122L002

TDR154S95L002

TDR159S106L002

TDR160S118L002

TDR162S130L002

TDR165combined

TDR166S153L002

TDR169S165L002

TDR170S177L002

TDR171S96L002

TDR173S107L002

TDR176S119L002

TDR177S131L002

TDR178S143L002

TDR179S154L002

TDR188S166L002

TDR190S178L002

TDR192S97L002

TDR195S108L002

TDR196S120L002

TDR1S86L002

TDR201S144L002

TDR203S155L002

TDR205S167L002

TDR207S186L002

TDR209S187L002

TDR20S134L002

TDR210S188L002

TDR212S189L002

TDR213S190L002

TDR214S191L002

TDR215S192L002

TDR216S193L002

TDR217S194L002

TDR218S195L002

TDR21S146L002

TDR220S197L002

TDR226S198L002

TDR227S199L002

TDR228S200L002

TDR229S201L002

TDR230S202L002

TDR231S203L002

TDR232S204L002

TDR233S205L002

TDR234S206L002

TDR235S207L002

TDR23S157L002

TDR24S169L002

TDR25S88L002

TDR26S100L002

TDR27S111L002

TDR28S123L002

TDR2S98L002

TDR30S135L002

TDR31S147L002

TDR32S158L002

TDR33S170L002

TDR34S89L002

TDR35S101L002

TDR36S112L002

TDR37combined

TDR39S124L002

TDR40S136L002

TDR44S148L002

TDR46S159L002

TDR47S171L002

TDR48S90L002

TDR49S210L002

TDR4S109L002

TDR50S113L002

TDR51S125L002

TDR52S211L002

TDR53combined

TDR54S149L002

TDR55S160L002

TDR57S91L002

TDR58S102L002

TDR59S114L002

TDR5S121L002

TDR60S126L002

TDR61S138L002

TDR62S212L002

TDR63S161L002

TDR64S173L002

TDR66S92L002

TDR67S103L002

TDR68S115L002

TDR69S127L002

TDR6S133L002

TDR71S139L002

TDR73S150L002

TDR76S162L002

TDR83S174L002

TDR84S93L002

TDR8S145L002

TDR9S156L002

**Supplementary Data 2**: Genetic differences between closely related strains

| Strain1 | Strain2 | SNP difference (PE/PPE and DR regions excluded) | SNP difference (PE/PPE regions included, DR regions excluded) | SNP and indel difference (PE/PPE regions included, DR regions excluded) |
| --- | --- | --- | --- | --- |
| M07 | M38 | 3 | 3 | 5 |
| M07 | M03 | 1 | 1 | 3 |
| M07 | M15 | 5 | 6 | 8 |
| M07 | M22 | 2 | 3 | 3 |
| M08 | M13 | 4 | 7 | 14 |
| M08 | M16 | 3 | 7 | 16 |
| M08 | M47 | 5 | 8 | 15 |
| M08 | M04 | 5 | 7 | 16 |
| M08 | M05 | 1 | 2 | 3 |
| M08 | M25 | 4 | 7 | 15 |
| M08 | M21 | 4 | 7 | 14 |
| M08 | M20 | 4 | 9 | 20 |
| M09 | M10 | 2 | 7 | 10 |
| M09 | M13 | 5 | 5 | 7 |
| M09 | M14 | 0 | 2 | 3 |
| M09 | M16 | 4 | 5 | 7 |
| M09 | M01 | 0 | 1 | 3 |
| M09 | M17 | 3 | 5 | 8 |
| M09 | M25 | 5 | 7 | 10 |
| M09 | M06 | 2 | 2 | 3 |
| M09 | M21 | 5 | 5 | 8 |
| M09 | M20 | 3 | 7 | 13 |
| M09 | M19 | 5 | 6 | 10 |
| M09 | M30 | 3 | 7 | 13 |
| M34 | M41 | 5 | 5 | 8 |
| M02 | M11 | 2 | 3 | 4 |
| M02 | M26 | 3 | 8 | 11 |
| M02 | M37 | 4 | 6 | 10 |
| M02 | M42 | 1 | 2 | 4 |
| M02 | M24 | 3 | 5 | 8 |
| M10 | M13 | 5 | 10 | 11 |
| M10 | M14 | 2 | 7 | 9 |
| M10 | M16 | 4 | 10 | 15 |
| M10 | M01 | 2 | 6 | 7 |
| M10 | M17 | 3 | 8 | 9 |
| M10 | M25 | 5 | 10 | 12 |
| M10 | M06 | 0 | 5 | 5 |
| M10 | M21 | 5 | 10 | 15 |
| M10 | M20 | 5 | 12 | 19 |
| M10 | M19 | 3 | 7 | 12 |
| M10 | M30 | 3 | 10 | 15 |
| M11 | M26 | 5 | 11 | 12 |
| M11 | M42 | 3 | 3 | 6 |
| M11 | M24 | 5 | 8 | 9 |
| M26 | M42 | 4 | 10 | 14 |
| M12 | M23 | 1 | 3 | 4 |
| M13 | M14 | 5 | 7 | 8 |
| M13 | M16 | 1 | 2 | 3 |
| M13 | M01 | 5 | 6 | 8 |
| M13 | M47 | 3 | 5 | 9 |
| M13 | M04 | 3 | 4 | 5 |
| M13 | M05 | 5 | 7 | 13 |
| M13 | M25 | 2 | 4 | 8 |
| M13 | M06 | 5 | 5 | 6 |
| M13 | M21 | 2 | 2 | 7 |
| M13 | M20 | 2 | 6 | 12 |
| M13 | M31 | 4 | 7 | 15 |
| M13 | M56 | 4 | 7 | 12 |
| M13 | M29 | 3 | 5 | 8 |
| M14 | M16 | 4 | 7 | 10 |
| M14 | M01 | 0 | 1 | 2 |
| M14 | M17 | 3 | 3 | 4 |
| M14 | M25 | 5 | 5 | 8 |
| M14 | M06 | 2 | 4 | 4 |
| M14 | M21 | 5 | 7 | 12 |
| M14 | M20 | 3 | 5 | 7 |
| M14 | M19 | 5 | 6 | 9 |
| M14 | M30 | 3 | 7 | 12 |
| M37 | M42 | 5 | 8 | 13 |
| M38 | M03 | 2 | 2 | 4 |
| M38 | M22 | 3 | 4 | 5 |
| M42 | M24 | 2 | 5 | 8 |
| M03 | M22 | 1 | 2 | 4 |
| M16 | M01 | 4 | 6 | 10 |
| M16 | M47 | 2 | 5 | 8 |
| M16 | M04 | 2 | 4 | 4 |
| M16 | M17 | 5 | 8 | 12 |
| M16 | M05 | 4 | 7 | 15 |
| M16 | M25 | 1 | 4 | 8 |
| M16 | M06 | 4 | 5 | 7 |
| M16 | M21 | 1 | 2 | 5 |
| M16 | M20 | 1 | 6 | 8 |
| M16 | M31 | 3 | 7 | 11 |
| M16 | M30 | 5 | 10 | 18 |
| M16 | M56 | 3 | 7 | 14 |
| M16 | M29 | 4 | 7 | 11 |
| M01 | M17 | 3 | 4 | 6 |
| M01 | M25 | 5 | 6 | 9 |
| M01 | M06 | 2 | 3 | 4 |
| M01 | M21 | 5 | 6 | 10 |
| M01 | M20 | 3 | 6 | 12 |
| M01 | M19 | 5 | 5 | 9 |
| M01 | M30 | 3 | 6 | 10 |
| M47 | M04 | 0 | 1 | 5 |
| M47 | M25 | 3 | 3 | 8 |
| M47 | M21 | 3 | 5 | 5 |
| M47 | M20 | 3 | 5 | 5 |
| M47 | M31 | 5 | 8 | 16 |
| M47 | M56 | 5 | 8 | 13 |
| M04 | M25 | 3 | 4 | 8 |
| M04 | M21 | 3 | 4 | 10 |
| M04 | M20 | 3 | 6 | 8 |
| M04 | M31 | 5 | 7 | 11 |
| M04 | M56 | 5 | 7 | 14 |
| M17 | M25 | 4 | 4 | 10 |
| M17 | M06 | 3 | 5 | 5 |
| M17 | M19 | 4 | 5 | 9 |
| M17 | M30 | 2 | 6 | 12 |
| M05 | M25 | 5 | 9 | 18 |
| M05 | M21 | 5 | 7 | 15 |
| M05 | M20 | 3 | 9 | 19 |
| M25 | M06 | 5 | 7 | 11 |
| M25 | M21 | 2 | 4 | 8 |
| M25 | M20 | 2 | 4 | 7 |
| M25 | M31 | 4 | 7 | 18 |
| M25 | M56 | 4 | 7 | 15 |
| M25 | M29 | 5 | 5 | 8 |
| M06 | M21 | 5 | 5 | 9 |
| M06 | M20 | 5 | 9 | 14 |
| M06 | M19 | 3 | 4 | 9 |
| M06 | M30 | 3 | 7 | 12 |
| M18 | M28 | 0 | 3 | 4 |
| M18 | M27 | 5 | 7 | 20 |
| M21 | M20 | 2 | 6 | 12 |
| M21 | M31 | 4 | 7 | 19 |
| M21 | M56 | 4 | 7 | 14 |
| M21 | M29 | 5 | 7 | 17 |
| M20 | M31 | 4 | 5 | 10 |
| M20 | M56 | 4 | 5 | 9 |
| M20 | M29 | 5 | 7 | 11 |
| M28 | M27 | 5 | 6 | 18 |
| M31 | M30 | 4 | 5 | 6 |
| M31 | M56 | 2 | 2 | 3 |
| M31 | M29 | 3 | 6 | 8 |
| M19 | M30 | 2 | 5 | 7 |
| M19 | M29 | 5 | 6 | 12 |
| M30 | M56 | 4 | 5 | 7 |
| M30 | M29 | 5 | 9 | 15 |
| M56 | M29 | 3 | 6 | 11 |

**Supplementary Data 3**

| Sample Accession Number | Assigned Sample Number | NCBI BioProject Number |
| --- | --- | --- |
| M0000057_1 | M01 | PRJNA526078 |
| M0000149_6 | M02 | PRJNA343736 |
| M0000349_2 | M03 | PRJNA343736 |
| M0000658_6 | M04 | PRJNA343736 |
| M0000691_7 | M05 | PRJNA343736 |
| M0000988_7 | M06 | PRJNA343736 |
| M0001017_4 | M07 | PRJNA343736 |
| M0001030_7 | M08 | PRJNA343736 |
| M0001205_5 | M09 | PRJNA343736 |
| M0001507_4 | M10 | PRJNA526078 |
| M0001573_6 | M11 | PRJNA526078 |
| M0001711_2 | M12 | PRJNA526078 |
| M0002097_5 | M13 | PRJNA526078 |
| M0002241_9 | M14 | PRJNA526078 |
| M0003655_9 | M15 | PRJNA526078 |
| M0005379_4 | M16 | PRJNA526078 |
| M0006866_9 | M17 | PRJNA526078 |
| M0009586_0 | M18 | PRJNA343736 |
| M0016200_9 | M19 | PRJNA343736 |
| M0021663_1 | M20 | PRJNA343736 |
| M0023894_0 | M21 | PRJNA343736 |
| M0000512_5 | M22 | PRJNA526078 |
| M0000786_5 | M23 | PRJNA526078 |
| M0000886_3 | M24 | PRJNA526078 |
| M0000939_0 | M25 | PRJNA526078 |
| M0001694_0 | M26 | PRJNA526078 |
| M0010396_1 | M27 | PRJNA343736 |
| M0011593_2 | M28 | PRJNA343736 |
| M0017196_8 | M29 | PRJNA343736 |
| M0022383_5 | M30 | PRJNA343736 |
| M0023766_0 | M31 | PRJNA343736 |
| M0001073_7 | M32 | PRJNA526078 |
| M0001255_0 | M33 | PRJNA526078 |
| M0001269_1 | M34 | PRJNA343736 |
| M0001428_3 | M35 | PRJNA526078 |
| M0002011_6 | M36 | PRJNA526078 |
| M0000258_5 | M37 | PRJNA526078 |
| M0002609_7 | M38 | PRJNA526078 |
| M0002752_5 | M39 | PRJNA526078 |
| M0002805_1 | M40 | PRJNA526078 |
| M0003241_8 | M41 | PRJNA526078 |
| M0003296_2 | M42 | PRJNA526078 |
| M0003308_5 | M43 | PRJNA526078 |
| M0005068_3 | M44 | PRJNA526078 |
| M0005397_6 | M45 | PRJNA526078 |
| M0005625_0 | M46 | PRJNA526078 |
| M0000652_9 | M47 | PRJNA526078 |
| M0000730_3 | M48 | PRJNA343736 |
| M0000872_3 | M49 | PRJNA526078 |
| M0021514_6 | M50 | PRJNA343736 |
| M0014878_4 | M51 | PRJNA343736 |
| M0021872_8 | M52 | PRJNA343736 |
| M0010874_7 | M53 | PRJNA343736 |
| M0014667_1 | M54 | PRJNA343736 |
| M0018274_2 | M55 | PRJNA343736 |
| M0019418_4 | M56 | PRJNA343736 |
| M0010598_2 | M57 | PRJNA343736 |
| M0005982_5 | M58 | PRJNA343736 |
| M0012861_2 | M59 | PRJNA343736 |
| M0017310_5 | M60 | PRJNA343736 |
| M0024214_0 | M61 | PRJNA343736 |
